# Piperine analogs arrest *c-myc* gene leading to downregulation of transcription for targeting cancer

**DOI:** 10.1038/s41598-021-01529-3

**Published:** 2021-11-25

**Authors:** Nirali Pandya, Amit Kumar

**Affiliations:** Department of Biosciences and Biomedical Engineering, Indian Institute of Technology Indore, Simrol, Indore, Madhya Pradesh 453552 India

**Keywords:** Cancer therapy, Biochemistry, Biological techniques, Biophysics, Chemical biology

## Abstract

G-quadruplex (G4) structures are considered a promising therapeutic target in cancer. Since Ayurveda, Piperine has been known for its medicinal properties. Piperine shows anticancer properties by stabilizing the G4 motif present upstream of the *c-myc* gene. This gene belongs to a group of proto-oncogenes, and its aberrant transcription drives tumorigenesis. The transcriptional regulation of the *c-myc* gene is an interesting approach for anticancer drug design. The present study employed a chemical similarity approach to identify Piperine similar compounds and analyzed their interaction with cancer-associated G-quadruplex motifs. Among all Piperine analogs, PIP-2 exhibited strong selectivity, specificity, and affinity towards *c-myc* G4 DNA as elaborated through biophysical studies such as fluorescence emission, isothermal calorimetry, and circular dichroism. Moreover, our biophysical observations are supported by molecular dynamics analysis and cellular-based studies. Our study showed that PIP-2 showed higher toxicity against the A549 lung cancer cell line but lower toxicity towards normal HEK 293 cells, indicating increased efficacy of the drug at the cellular level. Biological evaluation assays such as TFP reporter assay, quantitative real-time PCR (qRT- PCR), and western blotting suggest that the Piperine analog-2 (PIP-2) stabilizes the G-quadruplex motif located at the promoter site of *c-myc* oncogene and downregulates its expression. In conclusion, Piperine analog PIP-2 may be used as anticancer therapeutics as it affects the *c-myc* oncogene expression via G-quadruplex mediated mechanism.

## Introduction

Cancer is considered as the second most common reason of death worldwide^1^. According to the World Health Organization (WHO) report, 10.0 million cancer deaths were caused due to cancer in 2020^2^. It is estimated that in low and middle-income countries, around 70% of deaths occur due to cancer^2^. Therefore, scientists focus on the production of novel anti-cancer drugs development with an innovative mechanisms of actions. In this regard, precision medication could be an interesting therapeutic approach in which the treatment is provided according to specific genetic profile, types of cancer, and its stages^3–5^. In this area, researchers have found promising results, and new studies are being conducted to identify new drugs and targets^6,7^. However, critical issues like tumor heterogeneity and low benefit/cost ratio are unresolved yet^8^. Also, there is a possibility that the identified drug may not be active for the other cancer form or that it will be harmful to patients. To overcome this limitation, a promising approach could be to identify specific molecular targets common to all tumor cells and forms.

Numerous drugs have been identified that directly target the DNA through intercalation or groove mode of binding and hamper the cellular mechanisms (DNA replication, transcription, and translation). The canonical (duplex DNA), and the non-canonical DNA structures, including triplex DNA, Z-DNA, and G4 DNA structures, are promising targets for anticancer therapeutics^9–12^. The presence of different kind of polymorphism in the non-canonical DNA structures plays an important role in regulating the different cellular functions of human genome^13^. The G-quadruplex DNA is a four-strand DNA structure held together with Hoogsteen hydrogen bonding. In the human genome, 376,000 sequences have the ability to form a G-quadruplex structure, according to bioinformatics research^14–16^. Various oncogene’s promoter (*c-myc, c-kit, k-ras,* and *bcl-2*) regions and the end of the human telomeric region are rich in G-quadruplex motifs^12,17–19^. Moreover, the G4 forming sequences are also present in the regulatory region of the bacteria and viruses such as *Mycobacterium tuberculosis*, *Streptococcus pneumonia*, *Salmonella enterica*, *Vibrio Cholera*, *Nipha virus, Adeno Virus, and SARS-CoV-2* regulate the expression of the motif harboring genes^20–26^. Recent reports suggest that several RNA sequences also have G-quadruplex motifs, indicating their involvement in regulating their maturation via alternative splicing and translation and may be augmented to design drugs against various diseases^27,28^.

The human *c-myc* gene is a well-known proto-oncogene, and it produces the Myc protein, which plays a significant role in cell development, differentiation, and apoptosis^29^. The overexpression of the *c-myc* gene has been reported in an ample number of cancers, including breast, lungs, cervix, colon, osteosarcoma, colon, and glioblastoma, as it leads to inhibition of feasible cellular proliferation and differentiation^30,31^.

The *c-myc* gene amplification abnormality is manifested in the cancer genome as a genetic alteration^32–35^. *c-myc* is reported to play a crucial role in chemoresistance as it modulates the oxidation stress and strengthens the energy generation pathways^36^. Due to their critical role in cancer, there are multiple anti-*c-myc* therapies that results in the inhibition of transcription and translation activity of *c-myc*^37^. One of these strategies involves focusing on the *c-myc* regulatory promoter region, which contains nuclease hypersensitive element III1, which regulates ~ 85% of the gene’s transcription^38–40^. These regions contain 27 base pair guanine-rich sequences, which can fold into a G4 structure and act as a transcription repression element^38,41–43^. The NMR-based structural data of *c-myc* has shown that these G-rich sequences form polymorphic structures in potassium salt, and its major components are arranged in 1:2:1 loop form^41,44^. This G-quadruplex motif, when stabilized by various G4 specific ligands, plays a crucial role in the downregulation of *c-myc* protein. Since *c-myc* is reported to be highly overexpressed in a large number of cancers, specific drugs promoting the G4 formation at the promoter site of this oncogene may reinforce anti-cancer activity by accomplishing multi-target methods^45,46^.

In the past decade, several G-quadruplex ligands representing different structural classes have been reported to bind and stabilize a variety of G4 structures of proto-oncogene and inhibit its transcription process^45,47–56^. Thus, these molecules are corroborated as promising anticancer agents. In light of the aforementioned facts, two G4 binding ligands have succeeded in progressing into clinical trial. Among them, Quarfloxin, which is used to treat neuroendocrine/carcinoid tumours, is in a Phase II clinical trial, while CX-5461, which is used to treat breast cancer, is in a Phase I trial (Canadian trial, NCT02719977)^57,58^. Owing to its systemic toxicity and poor bioavailability, no G4 stabilizer has been approved as a medication. Thus, effective G-quadruplex specific ligands are investigated by a scientific community that may strongly interact with the genomic G4 DNA and can discriminate the duplex DNA as it contains chemical similarity with G4 structure in the cell. These effective strategies reduce the off-target effect and help to overcome toxicity. In general, it is considered that the following structural characteristics should be present in a small molecule to stabilize the G4 structure: (a) a planar structure of a molecule helps to fit in G-tetrad (b) a heteronuclear ring structure that can interact with the bases of DNA (c) cationic center of small molecules/ligand that can interact with the phosphate backbone of nucleic acid^59,60^. In this context, researchers are engaged to discover the novel and potent small molecules containing flexible charged moieties that recognize G4 structure and strongly interactvia stacking or groove mode^61^. In particular, numerous small molecules such as anilinoquinazoline, quindoline, carbazole, acridone, phenanthroline, curcumin, and their analogs have been identified, which bind and stabilize the *c-myc* G4 motif. As a result, the transcription and translation activity of *c-myc* gene get downregulated^54,62–65^. Additionally, water-soluble dyes have also shown strong interaction with *c-myc* G4 as fluorescence probes^66,67^. Thus, targeting the specific G4 structure with the help of small molecules can lead to designing a new anti-cancer drug. Moreover, the sensing of G4 in the cellular study could aid in deciphering its function and understanding the influence of small molecules on the transcriptional and translation process.

**Figure 1:**
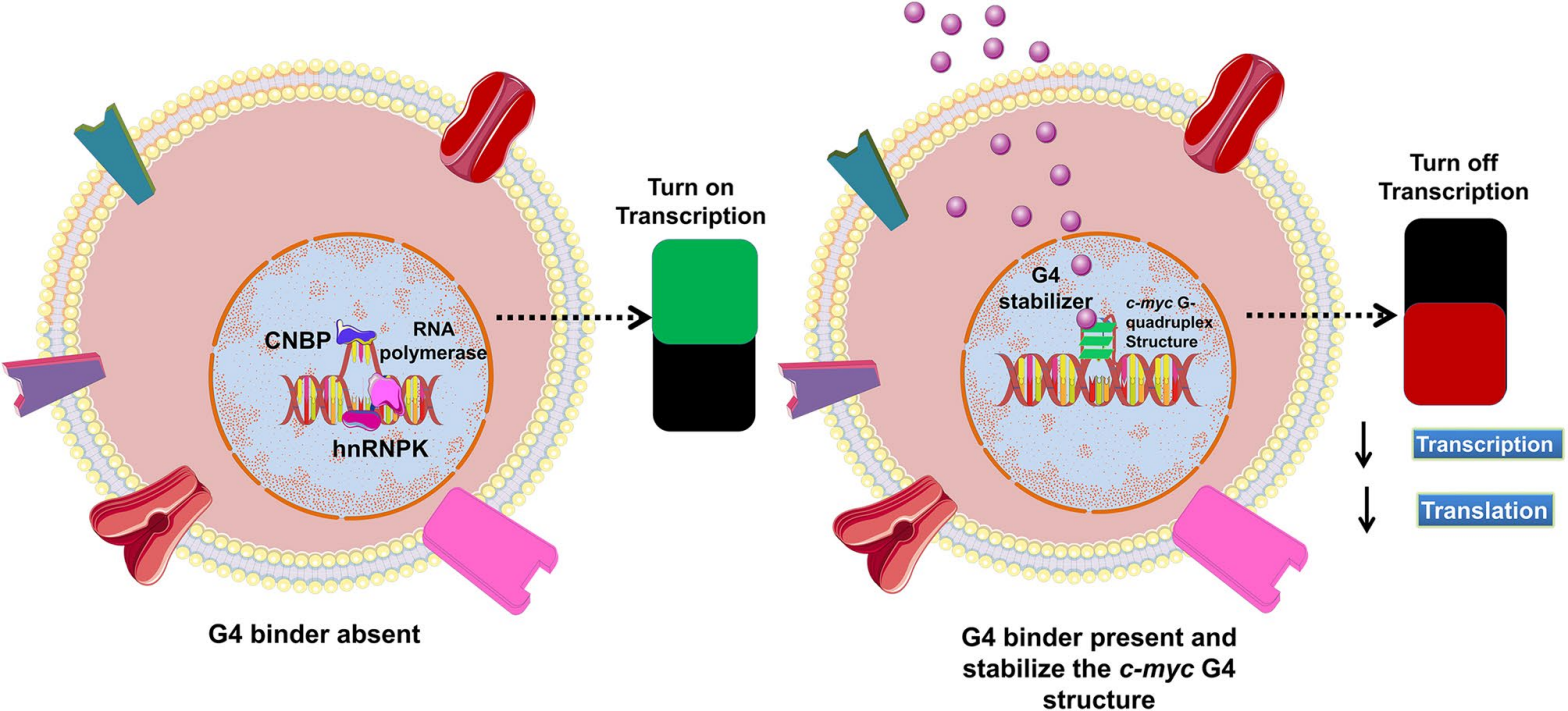
Schematic representation related to the influence of *c-myc* gene expression in the absence and presence of G4 binder.

Piperine also known as, 1-[5-[1,3-benzodioxol-5-yl]-1-oxo-2,4-pentadienyl] piperidine, belong to a nitrogenous natural plant alkaloid, and is obtained from black pepper (Piper nigrum) of the cinnamamides group^68^. It consists of important physiological and phytochemical^69^, anti-microbial^70^, anti-fungal^70^, anti-oxidant^71^, anti-apoptotic^72^, anti-depressant^73^, anti-oxidant^71^, and anti-inflammatory properties^72^. Piperine has a methylenedioxyphenyl (MDP) ring, a side chain with conjugated double bonds, and a piperidine moiety bound to a side chain by a carbonyl amide linkage^74^. The planer structure of Piperine provides π planer core that could give a suitable configuration to interact with the quadruplex structure with increased affinity and selectivity through π–π interactions^75^. Piperine interacts with and neutralizes the toxic effect of extended CGG repeats, which is essential in the treatment of neurological disorders like Fragile X Associated Tremor/Ataxia Syndrome^76^. Moreover, this natural molecule is also used for the treatment of leishmaniasis, malaria, and leukemia^77^. In an earlier report, our group has successfully proven that Piperine stabilizes the G4 present in the promoter region of *c-myc* oncogene via intercalating interaction and downregulates its transcription^45^. Indeed, the presence of functional aromatic core with multiple and diverse pendant groups on its aromatic core as well its chemical accessibility allowed modulating its interaction with the G4 motif^45^. In the current study, we focused on Piperine, among the plethora of scrutinizing G-quadruplex specific ligands^19,78,79^. It was speculated to be the most promising drug due to its good pharmacokinetics and have found various Piperine analogs that showed higher binding affinity towards G-quadruplexes as compared to the parent molecule. To investigate the effect of substitution of the Piperine core and the chemical nature of the substitutes on the interaction with G-quadruplex structure, we have screened the NCI library by using a chemical similarity approach taking Piperine as a query molecule. This approach of exploring compound with similar shape and chemistry helps to identify new small molecules with better pharmacodynamics and pharmacokinetics properties. We selected 12 Piperine analogs based on the Piperine core scaffold. The Piperine analogs used in this study contain two benzene rings except PIP-1 and along with alkyne, amino, sulfonamide hydroxyl, nitro and methyl groups in its structure. Further, we investigated the influence of these Piperine analogs on the binding property of *c-myc* G4 DNA by exploiting different biophysical techniques such as fluorescence spectroscopy, Isothermal Titration Calorimetry (ITC), Circular Dichroism (CD), gel retardation, and PCR stop assay. After spectroscopic analysis, it was verified that PIP-2 was a more prominent Piperine analog over other analogs. Therefore, we have selected the PIP-2 molecule for further in vivo studies. We carried out different cellular assay (MTT, mTFP reporter assay, wound healing assay, qRT-PCR, and western blotting) in which PIP-2 exhibited remarkable activities to stabilize the *c-myc* G4 DNA structure, accumulated in the nucleus, and downregulates the transcription and translation of *c-myc* gene and protein in human lung cancer cells (A549). In summary, our results illustrated here the interesting correlation that can be used to develop a new, more selective and active drug against the G4 motif of *c-myc* oncogene which regulate its expression by stabilizing its structure (Fig. 1).

## Material and methods

### Lead content and cell lines

Sigma-Aldrich Chemicals Ltd provided all reagents and solvents, including NaCl, KCl, KH_2_PO_4_, K_2_HPO_4_, NaH_2_PO_4_, Na_2_HPO_4_, EDTA (HPLC Grade), dimethyl sulphoxide (DMSO), and Piperine molecule. The National Cancer Institute in the United States provided Piperine analogs. Piperine and Piperine analogs stock solutions were prepared in DMSO and stored at the appropriate temperature.

Sigma Aldrich Chemicals Ltd., USA, also provided calf thymus DNA (CT duplex DNA) and other G-quadruplex DNA. The concentration of CT duplex DNA solution was determined spectrophotometrically after it was prepared in sodium phosphate buffer. Oligomers were dissolved in a 10 mM potassium phosphate buffer containing a 50 mM KCl at pH 7 for G-quadruplex formation analysis (*c-myc, bcl-2, tel22,* and *c-kit21*). Unless otherwise specified, all biophysical experiments were carried out in the buffer, as previously described. To allow gradual cooling, the oligonucleotides were heated at 90 °C for 5 min before being incubated at room temperature overnight. The *c-myc* G4 DNA oligonucleotides were combined with their respective reverse complementary oligonucleotides to form duplex DNA. The sequences of G-quadruplex DNA used in this study are mentioned in Table S1.

HeLa (Cervical cancer cells), A549 (Lung cancer cells), DU (Prostate cancer cells), A431 (Skin cancer cells), MCF-7 (Breast cancer cells), and HEK293 (human embryonic kidney cells) were purchased from National Centre for Cell Science (NCCS), Pune, India. Life Technologies (Gaithersburg, MD, USA) provided Dulbecco’s updated Eagle’s medium (DMEM) and other cell culture chemicals like 3-(4,5-dimethylthiazol-2-yl)-2,5-diphenyltetrazolium Bromide. Thermo Fisher supplied anti-*c-myc* and anti-*GAPDH* antibodies. Life Technologies provided Lipofectamine 2000, OptiMEM (Invitrogen Pvt. Ltd.), and ProLong Gold Antifade Reagent with DAPI, Hoechst 33342 (H), and Propidium iodide (PI) (USA). Thermofisher Scientific provided Ethidium Bromide (EtBr) and Acridine orange (AO) (A1301) for the cell culture experiments.

### Chemical similarity screening

The chemical similarity search was performed earlier reported^80^. By using ROCS (version 3.2.0.4, OpenEye Scientific Software)^81^ (https://www.eyesopen.com/rocs) and OMEGA (version 2.5)^82^. The molecules were ranked based on Tanimoto Combo scores according to their potential hit. Discovery studio 4.0^83^ software was employed for the energy minimization of the ligands by applying MMFF94 force field. The chemical structures of Piperine analogs were drawn by using chem sketch software^84^.

### Steady-state fluorescence binding assay

The fluorescence binding assay was performed on Synergy H1 multi-mode plate reader. The serial dilution of 10 µM of all G4 DNAs, CT duplex DNA, *c-myc* mutant and *c-myc* duplex DNA was performed in 2 nM Piperine analogs solution with no DNA added in the last well that served as a control. The fluorescence of the ligand was measured after it was excited with a particular wavelength (Supplementary Table S3). All data set’s changes in emission fluorescence were normalized using the only ligand-containing regulation. Using Sigma Plot 14.0^85^ software (Systat Software Inc., San Jose, California, USA) (http://www.sigmaplot.co.uk/downloads/download.php), the two-site saturation binding mode was used to plot the curve between the change in fluorescence (∆F) and concentration of G-quadruplex DNA/CT duplex DNA according to the following equation, which accounts for two receptor binding sites with two different affinities—K_d_^1^ and K_d_^2^:

1 $$f = \frac{{B_{max}^{1} \times abs\left( x \right)}}{{k_{d}^{1} + abs\left( x \right)}} + \frac{{B_{max}^{2} \times abs\left( x \right)}}{{k_{d}^{2} + abs\left( x \right)}}$$

where B_max_ is the maximum number of binding sites and K_d_ is the equilibrium binding constant.

The experiment was performed in triplicate and the average value was taken for binding affinity analysis.

### Isothermal titration calorimetry (ITC)

Isothermal titration calorimetry (ITC) experiments were employed by using MicroCal iTC200 Isothermal titration calorimeter (GE Healthcare, Biosciences Ltd., Sweden). Before performing the experiment, DNA samples prepared in a 10 mM potassium phosphate buffer containing 50 mM KCl at pH 7.0 were degassed for 3–5 min to remove air bubbles. In each titration, 1.78 µl of ligand solution containing 1 mM of Piperine analogs were injected into 15–20 µM of G-quadruplex DNA, *c-myc* G4 mutant, *c-myc* duplex and CT duplex DNA solution with the help of an automated syringe. Then, each ligand was titrated in buffer alone to evaluate the heat of dilution produced by the ligand. The heat produced due to the binding of ligand with DNA from each injection was calculated after correction with the heat of dilution. The subtracted heat values were plotted against the D/N molar ratios that give the binding isotherms. The raw data was analyzed using MicroCal Origin software and fitted using a two-mode fitting model to calculate the association constant (Ka), change in entropy (∆S), and change in enthalpy (∆H), and the change in Gibb’s free energy (∆G) was measured using the following equation.

2 $$[\Delta {\text{G}} = - {\text{RT}}\ln \;({\text{Ka}})]$$

The ITC experiments performed between Piperine analogs and DNA (G-quadruplex and control DNA) were replicated twice, with a measurement difference of less than 10%.

### Fluorescence emission spectroscopy

Fluorescence emission spectroscopy was performed for PIP-2 analog and *c-myc* G4 DNA by using Flurolog Horiba Scientific Spectrofluorimeter. The quartz cuvette of 1 cm path length was used to perform the experiment. By progressive addition of *c-myc* G4 DNA at a constant concentration (5 µM) of PIP-2 ligand, emission spectra in the wavelength range of 390–600 nm with different D/N ratios (D/N ratio 0.10–5.0) were obtained.

### Circular dichroism (CD) melting and CD spectra

CD experiments were done on J-815 Spectropolarimeter (JASCO) equipped with a Peltier junction thermal controller. For CD spectral analysis, the spectra of *c-myc* G-quadruplexes DNA were taken in 10 mM potassium phosphate buffer (pH 7.0) in the presence of 50 mM KCl. Further, the Piperine analogs were titrated from D/N 0 to 5, and the spectra was recorded. For melting analysis, the G-quadruplex DNA (10 µM) dissolved in 10 mM potassium phosphate buffer (pH 7.0) containing 50 mM KCl were thermally denatured from 25 to 95 °C with a rate of 1 °C/min at 265 nm. In individual experiments, the different Piperine analogs were then titrated in the *c-myc* G4 DNA solution up to D/N 5.0. To further perform the melting analysis of PIP-2 analog with other G-quadruplex DNAs (*bcl-2*, *tel22*, and *c-kit*), DNA sample solution prepared in the 10 mM potassium phosphate buffer (pH 7.0) containing 50 mM KCl and were thermally denatured from 25 to 95 °C with a rate of 1 °C/min at their respective wavelength (265 nm for *bcl-2* and *c-kit*, & 290 nm for *tel22*). The resulting graphs were plotted by using Sigma Plot 14.0 software (Systat Software Inc., San Jose, California USA), and the changes in melting temperature (∆Tm) were calculated by using the following equation:

3 $$\Delta {\text{T}}_{{\text{m}}} = {\text{T}}_{{{\text{m}}({\text{DNA}} + {\text{ligand}})}} {-}{\text{T}}_{{{\text{m}}({\text{DNA}})}}$$

### Gel retardation assay

The *c-myc* G4 DNA and its mutant was prepared in potassium buffer (10 mM, pH 7.0) containing 50 mM KCl for the gel retardation experiment. For 30 min at 25 °C, 10 µM *c-myc* G4 DNA was incubated with different Piperine analogs ranging in concentration from 0.00 to 100 µM. The incubation results were then resolved on a 15% native polyacrylamide gel [29:1 acrylamide/bicarbonate]. The EtBr (Ethidium bromide) solution was used as a staining solution for the native page Gel and images were analyzed with the help of Image Quant LAS (GE health care, Bioscience Ltd, Sweden).

### PCR stop assay

PCR stop assay was performed using the 250 pmol *c-myc* G4 DNA, *c-myc* G4 mutant DNA and its reverse complementary primer sequences in 30 µl reaction volume. The other components present in the PCR reaction mixture were 1× buffer, 50 mM MgCl_2_, 1 mM dNTPs, and 5U *Taq* polymerase. The reaction mixture was incubated with various concentrations (0.00–100 µM) of Piperine analogs for 15 min at room temperature. The PCR (35 cycles) was performed in Mastercycler Nexus Gradient (Eppendorf) by employing the following condition: 94 °C for 30 s, 60 °C for 30 s, 72 °C for 30 s, and 72 °C for 2 min. The final PCR product was resolved on 3% agarose gel, and the images were analyzed.

### Molecular docking and dynamics simulation study

For the molecular dynamic study, the *c-myc* G4 structure was used as an initial model (PDB code: 2A5R)^86^. To perform the docking study, necessary changes like the addition or the replacement of residues were carried out in Discovery studio^83^. The docking analysis of Piperine analogs with *c-myc* G4 DNA was performed in Autodock 4.0^87^ using the Genetic algorithm and the previously published protocol^55^. The docking study of lead PIP-2 with other G-quadruplex structures such as *bcl-2* (PDB code: 2F8U)^88^, *tel22* (PDB code: 4G0F)^89^, and *c-kit21* (PDB code: 2KYP)^90^ were also carried out by using Autodock 4.0^87^. During docking, Gasteier chargers were added on *c-myc* G4 DNA as well as other G-quadruplex DNA. The Piperine analogs were able to explore the entire conformational space by creating a grid box that covered the *c-myc* G4 DNA. The results were studied based on the minimum binding energy and hydrogen bond formed between *c-myc* G4 DNA and Piperine analogs. To further investigate the binding of Piperine analogs with *c-myc* G4 DNA, the results of the first docking were used as feedback for the second docking without changing the parameters. The same methods were used for the docking between lead PIP-2 with other G-quadruplex DNA. PyMOL^91^ and Discovery studio 4.0^83^ was used to interpret the results and formation of images. For molecular dynamics simulation (M.D.) studies, the best performance of docked complex (PIP-2 and *c-myc* G4 DNA) was used as an input. The M.D. simulation was carried out on NAMD in a water sphere using NAMD (Nanoscale Molecular Dynamics) standard molecular dynamics tool (https://www.ks.uiuc.edu/Research/namd/)^92^. By using the CHARMM force fields for DNA-ligand complex and apo *c-myc* DNA, the Visual Molecular Dynamics (VMD) tool v.1.9.3^93^ was used to generate the necessary structure files (.psf). The CHARMM-GUI server was used to build the ligand topology and parameter files. A 100-ns production run was completed, and the resulting trajectory file (DCD) was used to calculate the RMSD of the apo *c-myc* and *c-myc*-PIP-2 complex by employing CPPTRAJ^94^. The other parameters such as Root Mean square deviation (RMSF), Radius of gyration (Rg), Interaction energy, Number of hydrogen bonds, and Solvent accessibility surface area (SASA) were analyzed by using NAMD VMD plugin for apo *c-myc* and *c-myc*-PIP-2 complex. The images which represent the docking and DNA-ligand DNA interaction were created by using Pymol^91^, Discovery Studio 4.0^83^ and LIGPLOT^95^.

### Free energy surface (FES) landscape calculations

The movement of molecules may be discussed in terms of free energy. This includes movement including folding or aggregation. The dynamic behaviour of apo *c-myc* and *c-myc-* PIP-2 complex for the generation of free energy landscape was calculated by using appropriate sampling method. The RMSD and Rg of apo *c-myc* and *c-myc*-PIP-2 complex were selected as two reaction coordinates for the generation of dimensional energy landscape map. The following equation was used to calculate the energy landscape along these two reactions coordinates.

4 $$\Delta G\left( R \right) = - k_{{\text{B}}} T\left[ {\ln \;{\text{P}}\left( {\text{R}} \right) - \ln \;{\text{P}}_{\max } } \right]$$

here k_B_ stands for the Boltzmann constant, T stands for the simulated temperature, and P stands for the normalized joint probability distribution. For the lowest free energy minimum, the free energy difference (ΔG) is set to zero, and the probability distribution (P) of the molecular system is represented by the function ƒ(P, R), where P is the probability of the system occurring along with the coordinate R, and P_max_ is its maximum. The two order parameters show where the free energy lies on the free energy surface (FES).

### MTT assay

HEK cells and cancers cells such as DU, HeLa, A549, A431, and MCF-7 cells were seeded in 96-well plates (7.0 × 10^5^ cells per/well). The cells were grown at 37 °C in complete DMEM media in a humidified atmosphere containing 5% CO_2_. The cells were exposed to different concentrations of Piperine analogs from 0.00 to 200 µM in triplicates for 24 h. After a 24-h incubation period, each well received 20 mL of 5 mg/mL methylthiazolyl tetrazolium (MTT) solution. The cells were then incubated for another 4 h. After that, the cells in each well were treated with 200 µl dimethyl sulfoxide (DMSO), and the optical density (OD) was measured at 590 nm. Both experiments were carried out in triplicate and in parallel, and the cell viability was calculated using the mean OD values of the triplicate plotted against the ligand concentration.

### Cellular intake and localization of the Piperine analog (PIP-2) inside the cells

At a density of 6 × 10^4^ cells/mL, A549 cells were grown on glass coverslips. PIP-2 (100 µM) was added to the cells and incubated at 37 °C for 4 h. A549 cells were washed in PBS after being incubated and stained with the nuclear staining dye Thiazole orange (TO). After that, the cells were washed in PBS, and paraformaldehyde was used to fix them (PFA). Before using the mounting media, the coverslips were washed once again. Images were taken for all the samples by using the confocal fluorescence microscope.

### Morphological examination of the PIP-2 treated A549 cells

To investigate the induction of apoptosis in A549 cells, the cells were plated in 6-well plates and treated with different concentrations of PIP-2 for 24 h. After being treated with PIP-2, the cells were washed in PBS and fixed for 15 min at room temperature with paraformaldehyde (PFA). The cells were then washed twice in PBS before being stained with the fluorescent dye 4′,6-diamidino-2-phenylindole (DAPI). Images were taken through confocal microscopy, and quantification was done with Image J software.

### Apoptosis analysis in PIP-2 treated A549 cells by Hoechst 33342/propidium iodide (PI) and acridine orange/ethidium bromide staining

The Hoechst 33342 and PI double staining was performed as described earlier^54^. In 6-well plates, 2 × 10^5^ A549 cells were seeded per well. The culture medium was replaced with a new medium containing various concentrations of PIP-2, and the cells were incubated for another 24 h. Afterward, the cells were washed in PBS and stained with Hoechst 33342 (H) and propidium iodide (PI) or acridine orange and ethidium bromide, respectively. Images were taken through confocal microscopy, and quantification was done with Image J software.

### Cell Scrape and colony formation assay

A549 cells were seeded in six well plates. After 24 h, a linear scrape was made in the monolayer using a cell scraper. The wounded areas were observed, and images were taken using phase-contrast microscopy after scratching. The cells were then treated with different concentrations of PIP-2 and incubated for 24 and 48 h. The area of the wound in the treated and untreated cells was quantified using the Image J software. 1 × 10^3^ cells/well were seeded into a six-well plate to study the impact of PIP-2 on colony formation in A549 and HEK293 cells. The cells were treated with PIP-2 and incubated for 24 h once the colonies were visible. The cells were washed in chilled PBS and fixed in a solution containing Methanol and acetic acid in 3:1 ratio. The cells were incubated in the fixing solution for 5 min. Afterwards, fixing reagents were removed and cells stained with 0.5% crystal violet solution (in Methanol) for 15 min. Then, the cells were washed with water and imaging were performed.

### Construction of *c-myc* and *c-myc* mutant G4 m-TFP based plasmid and perform m-TFP based reporter assay

The mTFP based assay was performed by using the previous protocol^54^. The PCR based mutagenesis approach was used to clone the *c-myc* G4 DNA and *c-myc* G4 mutant in mTFP-pCAG plasmid. To construct the clone, overlapping forward and reverse primers complimentary to the upstream of mTFP (monomeric teal fluorescent protein) protein in the mTFP-pCAG plasmid were used (a gift from Dr. Debasis Nayak, IIT Indore). The forward primer contained the 5′ overhanged *c-myc* or *c-myc* G4 mutant motif. To amplify the PCR product, the following thermal cycle conditions were used: initial denaturation at 95 °C for 5 min, annealing at 78 °C for 45 s, and extension at 72 °C for 10 min. The annealing and extension steps were repeated 30 times. DpnI cleaved the host plasmid, and the digested substance was converted into *E. coli* DH5α strains. After that, a Midi-prep isolation package (Himedia pvt. Ltd.) was used to purify the plasmid. Human embryonic kidney (HEK 293) cells were grown and seeded in 6 well culture plates until they reached 60–75% confluency. Following the manufacturer's protocol, the cells were transfected with the pCAG-*c-myc*-mTFP and pCAG-*c-myc*-mutant-mTFP plasmids using Lipofectamine 3000 (Invitrogen Pvt. Ltd.). PIP-2 was used to treat the transfected cells, and mTFP expression was measured using fluorescence microscopy.

### ROS generation analysis

A549 cells were seeded in a 6-well tissue culture plate and treated with PIP-2 to see whether it induced apoptosis or changed the redox status. After a 24 h incubation period, the cells were washed twice with PBS and stained for 30 min with 10 µM 2′–7′ dichlorofluorescin diacetate (DCFH-DA, Sigma-Aldrich) dye. Methanol was used to fix the cells, and fluorescence microscopy was used to image them. The quantification was done with Image J software.

### Reverse transcription polymerase chain reaction

The RT-PCR experiments were performed by using the same protocols^54^. 1 × 10^6^ of different cancer cells (HeLa, A549, A431, MCF-7, DU) cells were sub-cultured onto 6 well microtiter plates and treated with PIP-2 for 24 h and quantitative RT-PCR analysis were carried out to study the expression of *c-myc* gene. Similarly, A549 cells are also seeded in 6 well plate and treated with PIP-2 for 24 h and the expression of other oncogenes such as *bcl-2* and *c-kit* were analyzed by using quantitative RT-PCR. Complete RNA was extracted from treated and untreated cells using the Trizol method (Invitrogen) according to the manufacturer's instructions. cDNA was extracted from 1 g of RNA from both untreated and treated samples. Following that, RT-PCR was performed for 20 µl reactions using iTaq Universal supermix (BIO-RAD) SYBR Green (BIO-RAD) with the following thermal cycling conditions: initial denaturation at 92 °C for 5 min, denaturation for 30 s, annealing at 55 °C for 30 s, extension at 72 °C for 60 s for 30 cycles, and a final extension at 72 °C for 5 min followed by 4 ℃ for infinity. For the relative quantification of *c-myc* gene expression in different cancer cells and various oncogenes expression in A549 sample compared to the untreated control w.r.t. the housekeeping gene, *β-actin*, the comparative cycle threshold method (∆∆C_.T._) was used. The forward and reverse primer sequences used in the qRT-PCR were stated in the Supplementary Information Table S1.

### Western Blotting

PIP-2 concentrations of 3 µM, 6 µM, and 12 µM were used to treat A549 cells for 24 h. Using chilled RIPA cell lysis buffer, treated and untreated cells were lysed. The lysate of the cells was centrifuged for 15 min at 13,000 rpm at 4 °C. The BRADFORD method was used to quantify proteins in the cell lysate. A 70 g protein sample was isolated on a 12% SDS PAGE gel with a verticle separator. In a transfer buffer containing 25 mM Tris, 190 mM glycine, and 20% methanol, the gel was transferred onto a PVDF blot. Anti-*c-myc* (diluted to 1:1000 in TBST buffer) and anti-*GAPDH* (endogenous loading control diluted to 1:2000 in TBST buffer) antibodies were then incubated overnight on the blot membrane (from Thermo Fisher Scientific). The blot was then incubated for 2 h at 4 °C with an anti-mouse secondary antibody (Thermo Fisher) linked to Horseradish peroxidase diluted to 1:10,000 in TBST, and the image was analyzed using chemiluminescence in Image Quant LAS 4000. (GE Healthcare). Image J was used to examine relative band intensities.

### Quantification and statistical analysis

Prism (GraphPad) 7.0 (https://www.graphpad.com/), origin 7.0, and the sigma plot 14.0^85^ (http://www.sigmaplot.co.uk/downloads/download.php) were used for statistical analysis. A statistically relevant value of *p < 0.05, **p < 0.01, and ***p < 0.001was used. The standard deviation is represented by all error bars (S.D.). For quantitative details, the statistical parameters are mentioned in the figure legends. Image J was used to perform the strength quantification^96^.

## Result and discussion

### Chemical similarity search for Piperine similar analogs

**Figure 2:**
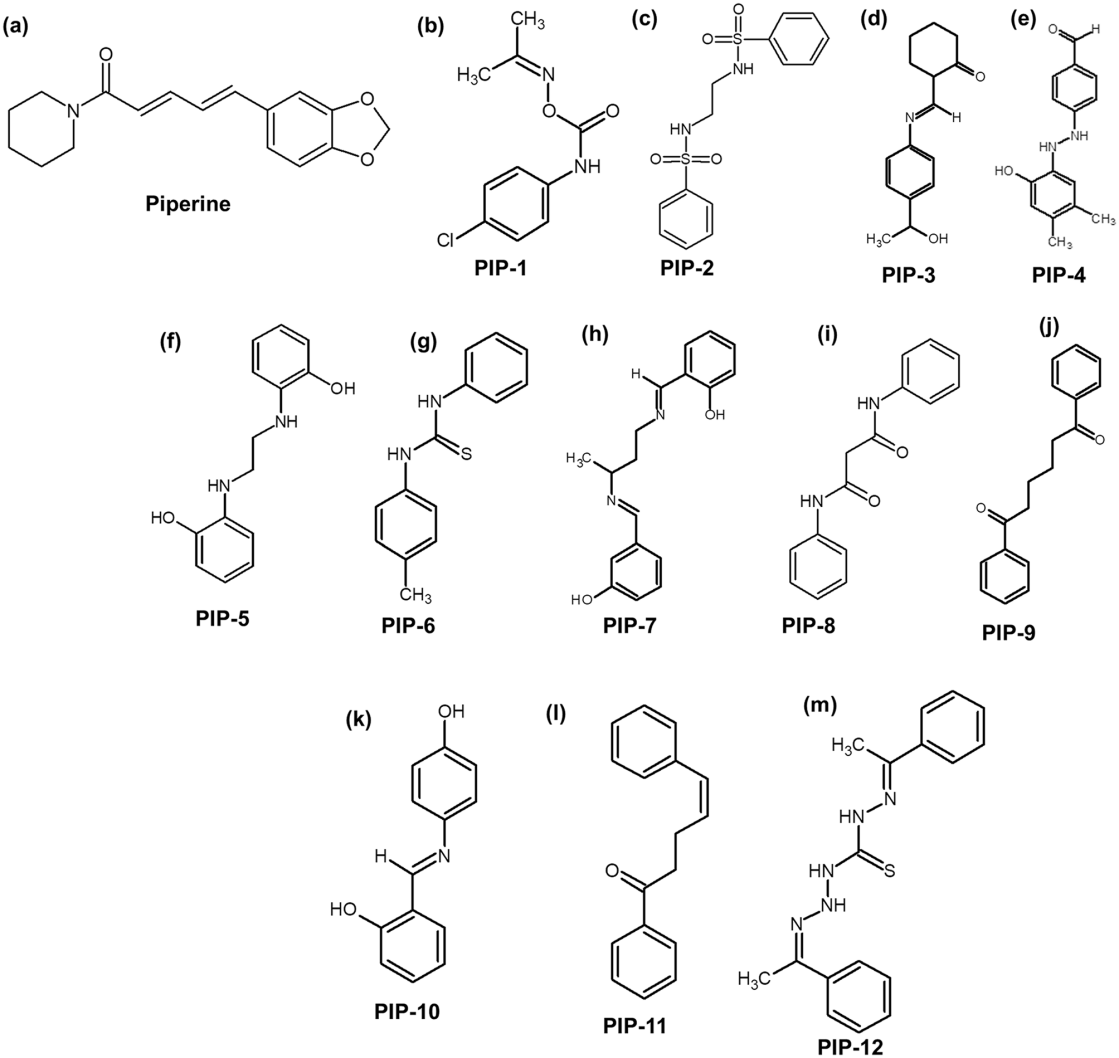
Chemical structure of Piperine (**a**) and Piperine analog molecules (**b**–**m**).

The 3D shape of the small molecule is a prominent molecular feature that determines its functions and activities^97,98^. The in silico approaches provide a robust platform for shape-based screening of small molecules for drug designing as it reduces the cost of drug discovery and development^97^. Molecules of similar shapes are not only provide the same binding pocket but also exhibit similar biological functions. In this report, we employed an approach to identify chemical similarity and selected small molecules similar to Piperine in the National Cancer Institute (NCI) compound library. The NCI database consists of a wide array of more than 250,000 small molecules, with different chemical shapes and stereochemistry information. To compare the 3D shape between the two molecules, the Rapid Overlay of Chemical Structures (ROCS)^99^ (https://www.eyesopen.com/rocs) software was used. For screening, we selected the molecules with complete stereochemistry specifications and 3D coordinates. There are two different matrices used for the evaluation of the chemical similarity search (1) Shape Tanimoto coefficient ^100^ (3D similarities) and (2) color core ^101^ (analyze the 3D alignment of hydrogen acceptor, donor, hydrophobic group, anions, cations, and rings). The results of similarity between two molecules, whether it belongs to shape or color core lies between the Tanimoto coefficient of 0–1 where 0 states for no similarity, and 1 represents the complete similarity. A group of the top 12 (Fig. 2 and Supplementary Table S2) molecules based on similarity with the Piperine molecule^45^ were selected and obtained from the NCI library. These molecules were screened for their binding affinity and selectivity against G-quadruplex DNA (*c-myc*, *bcl-2*, *tel22,* and *c-kit21*) located in the promoter region of various oncogenes.

### Steady-state fluorescence binding assay employed for the primary screening of Piperine analogs with *c-myc* G4 DNA

**Figure 3:**
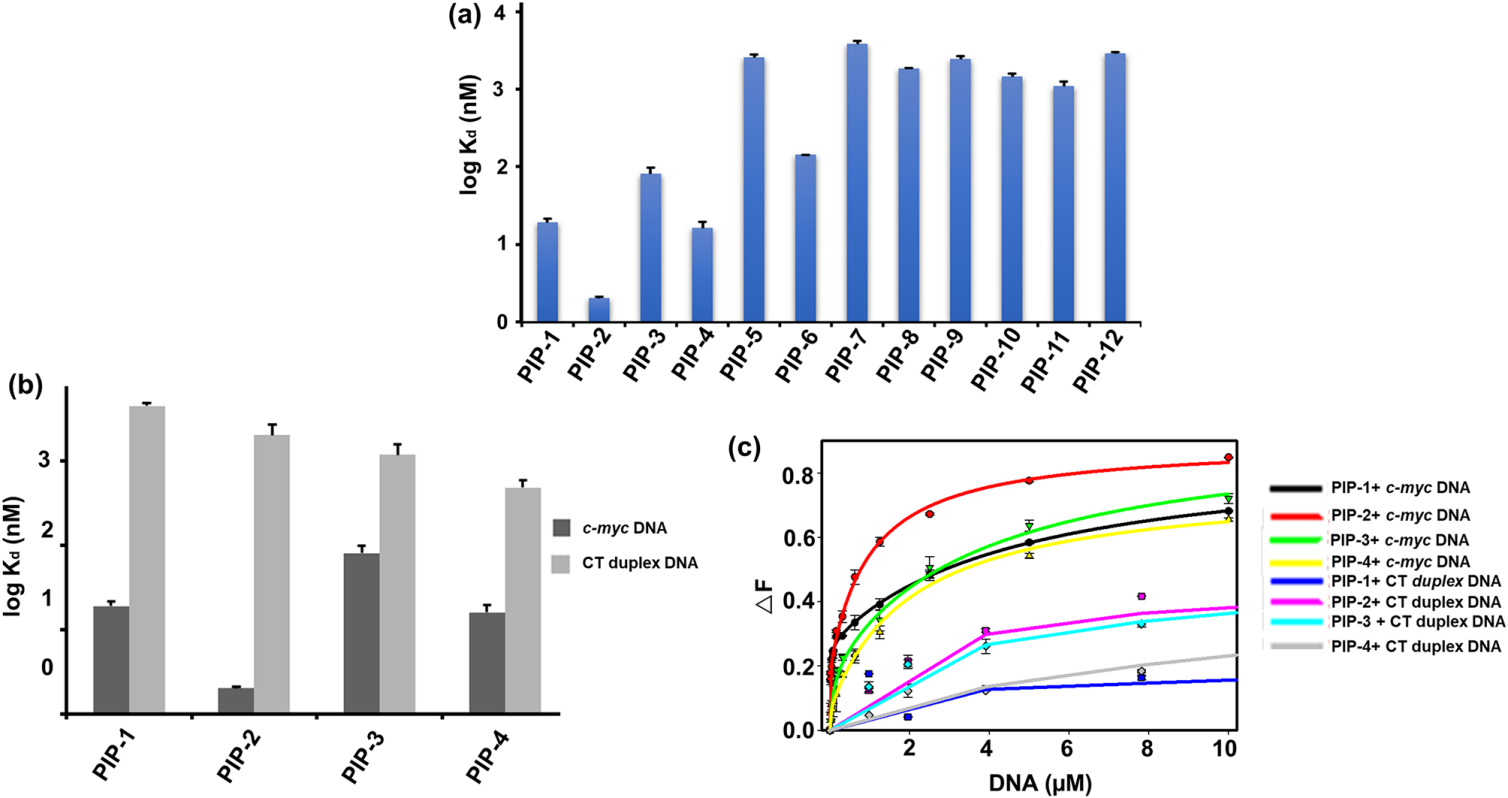
Screening of Piperine analogs with *c-myc* G4 DNA via steady-state fluorescence titration study. (**a**) The bar diagram represents the binding constant values of Piperine analogs (2 nM) with *c-myc* G4 DNA. (**b**) The comparative bar diagram depicts the K_d_ values of lead Piperine analogs with *c-myc* G4 and CT duplex DNA (Control). (**c**) A combined binding plot showed the change in the fluorescence intensity upon binding Piperine analogs with *c-myc* G4 DNA and CT duplex DNA.

c-Myc is a transcription factor that performs critical regulatory functions in many areas of transformation^102–104^. In Burkitt’s lymphoma tumor, the *c-myc* gene translocated in the heavy- and light-chain immunoglobulin genes and implicated in primary oncogenic functions. It is more generally known as a downstream “early-response” gene that reacts to the activation of a wide variety of signaling pathways^105^. In the last several years, researchers have identified small molecules of natural and synthetic origin which stabilize the *c-myc* G4 structure^54,106,107^. As mentioned earlier, our group has previously identified that Piperine exert its anti-cancer activity by stabilizing the *c-myc* G4 motif^45^. In this line, to identify the potent *c-myc* G4 binder Piperine analog, first, we performed a fluorescence binding assay to analyze the binding affinity of Piperine analogs with *c-myc* G4 DNA (Fig. 3a, Supplementary Table S4a). The emission maxima of all Piperine analogs were evaluated in their unbound form (Supplementary Table S3). The addition of G4 DNA in the ligand solution causes a change in the fluorescence intensity due to the binding of a ligand with *c-myc* G4 DNA and the formation of the ligand-G-quadruplex complex. In the interaction with the *c-myc* G4 structure, the Piperine molecule exhibited two-mode saturation binding model with stoichiometries 1:2 for DNA/Piperine^45^. Moreover, Piperine is also reported to interact with (CGG) repeats that also form G-quadruplex in 1:2 ratio^76^. Therefore, the binding curve plotted between change in the fluorescence intensity (∆F) and DNA concentration used for the experiment were also best fitted using the global two-mode saturation binding model^108^. Several small molecules belonging to these class of compounds also showed same kind of binding mode by forming stacking interaction with one or both the outer quadrates of G-quadruplex structure^108,109^. In the case of 1:2 stoichiometries, two molecules of Piperine analog intercalate into the duplex DNA and interact with the target G-quadruplex DNA with two sites either stacking or groove mode of binding. Thus, the presence of a higher number of the binding site in G4 structure with respect to duplex DNA, leads to the highest binding stoichiometries obtained for the duplex/Piperine analog systems, which may be correlated with the formation of unspecific electrostatic interaction and provide the higher affinity for G4 structure compared to duplex DNA. Interestingly, the dissociation constant (K_d_) value obtained for Piperine analogs PIP-1, PIP-2, PIP-3, and PIP-4 with *c-myc* G4 DNA suggested the higher binding affinity than other analogs (Supplementary Figs. S1, Figs. S4 and Table S4a). The *c-myc* gene inhibitor ligands should have a higher selectivity for the *c-myc* G4 motif over duplex DNA. The good G4 binder must be able to identify the G4 structure even in the presence of a large amount of duplex DNA in the cell nucleus. The ligand competes to interact with G4 structure over duplex DNA, thereby reducing the inhibitory function of the *c-myc* gene. Therefore, we proceeded further for the fluorescence binding experiment of all four lead Piperine analogs with CT duplex DNA. All the analogs showed ~ 100 fold lesser binding affinity with CT duplex DNA than *c-myc* G4 DNA that strengthening these molecules’ selectivity towards *c-myc* G4 DNA (Fig. 3b, Supplementary Fig. S2 and Table S4c). We have also analyzed the binding affinity of the lead Piperine analogs with *c-myc* mutant (unable to form G4 structure) and *c-myc* duplex DNA (equivalent to the same length of *c-myc* G-quadruplex), that showed the poor binding affinity of lead Piperine analogs towards these oligonucleotides as compared to the *c-myc* G4 DNA (Supplementary Fig. S3, and Table S4c). These results suggested the strong selectivity and affinity of Piperine analogs towards *c-myc* G4 DNA over used different control DNA (Figs. 3c and S6). Next, we have also carried out binding experiments of all the four lead Piperine analogs with other biologically relevant G4 structures (*bcl-2*, *c-kit21,* and *tel22*) to understand the effect of different topologies and loop sequences on the interaction with the lead Piperine analogs as the *bcl-2, c-kit21* and *tel22* form parallel and hybrid topology respectively^110–112^. The higher dissociation constant (K_d_) values for these G4 DNAs as compared to *c-myc* G4, indicated a higher binding affinity of Piperine analogs towards the *c-myc* G4 motif (Supplementary Fig. S4 and Table S4b). Previously, our group reported that Piperine exhibited a strong affinity and selectivity for *c-myc* G4 DNA than other G-quadruplexes^45^. Interestingly, in this study, we have found that PIP-2 molecule showed comparable K_d_ value to the Piperine molecule upon on interaction with *c-myc* G-quadruplex structure^45^. The higher binding affinity of Piperine and its analogs towards *c-myc* G4 compared to other G4 (*bcl-2*, *c-myc* and *tel22*) DNA might be due to the difference in the sequence, folding pattern, sequence strand direction, size of the loop, and topology of G4 DNA^113^. Thus, variation in the affinity of Piperine analogs with *c-myc* G4 DNA might be due to the selectivity of compounds towards its topology over other cancer causing G-quadruplex sequences.

The K_d_ values for the four best Piperine analogs with the *c-myc* G4 DNA were as follows; PIP-2 (K_d_^1^ = 0.002 ± 0.001 µM) > PIP-1 (K_d_^1^ = 0.010 ± 0.003 µM) > PIP-3 (K_d_^1^ = 0.08 ± 0.007 µM) > PIP-4 (K_d_^1^ = 0.016 ± 0.005 µM). The following trend implies that the different substitutes present on the aromatic ring and the side chain of the Piperine analogs could contribute to different binding orientations and environments to the *c-myc* G4 DNA structure. The highest fluorescence induction in PIP-2 might be possible due to the suppression of the ligand's free rotation and vibratory motion when it interacts with the pocket of the *c-myc* G4 DNA structure. The PIP-2 ligand structure contains two benzyl rings connected with the sulfonamide group that could interact with the *c-myc* G4 through hydrogen bonding. Several previously available scientific reports suggested that sulfonamides have contributed to mediate anti-tumor activity through unique therapeutics mechanisms^114,115^. On the other hand, PIP-1 and PIP-3 molecules have an amino side chain that might interact with the G-quadrate to increase electrostatic and hydrogen bonding interaction^116^. Piperine analog PIP-4 has two –CH_3_ groups on its aromatic ring structure, which might decrease the binding affinity with *c-myc* G4 DNA due to increased steric hindrance.

As a result of our primary screening, we have identified four lead molecules with a high affinity for *c-myc* G4 DNA. Further, we have performed the secondary screening of Piperine and its lead analogs with *c-myc* G4 DNA through an isothermal titration calorimeter (ITC).

### Isothermal titration calorimetry (ITC) assay of Piperine and its analogs with *c-myc* G4 DNA

**Figure 4:**
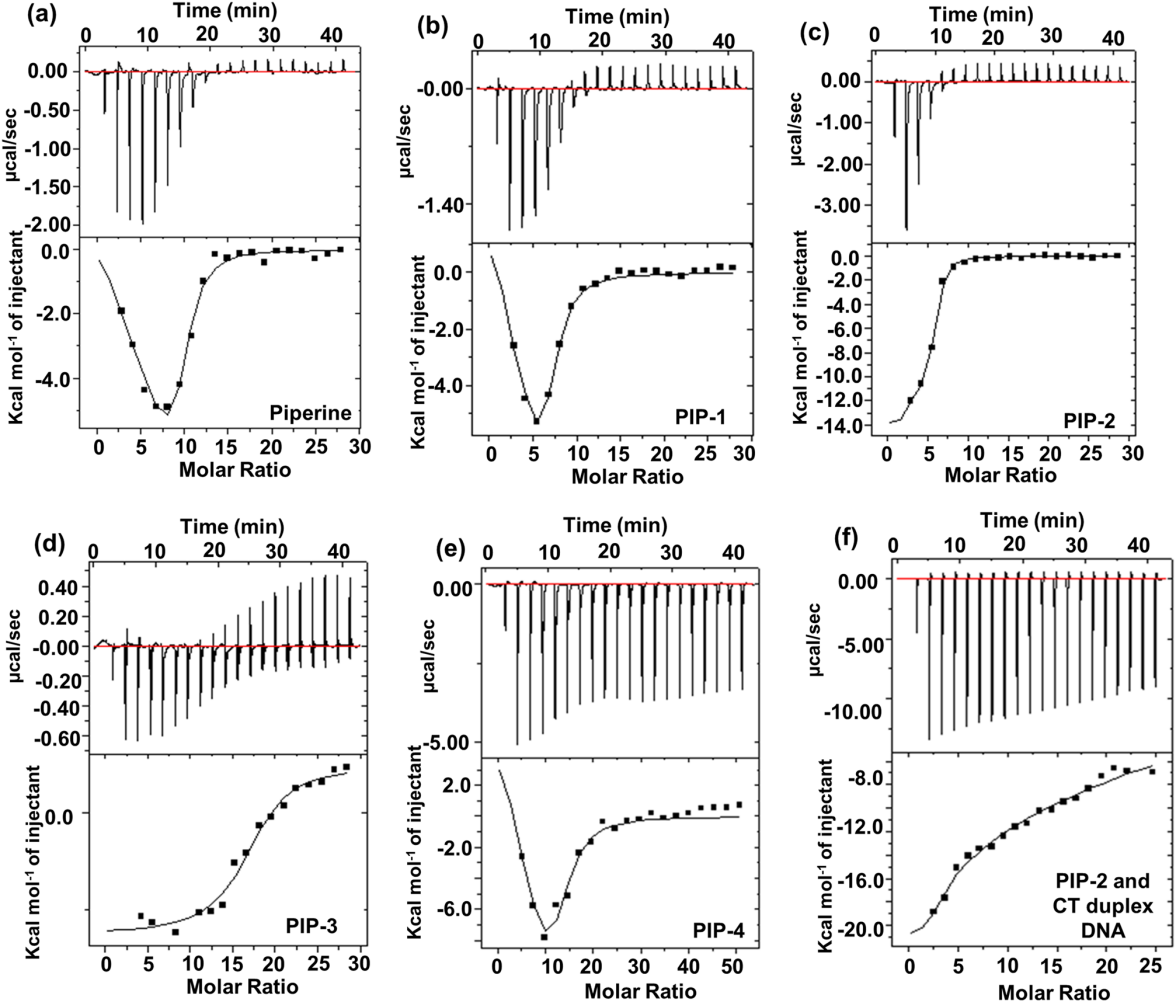
The ITC thermogram of Piperine analogs with *c-myc* G4 DNA (**a**–**e**) The upper panel of the ITC profile shows the isothermal titration thermogram of *c-myc* G4 DNA (in the cell) in (10 mM potassium phosphate buffer containing 50 mM KCl at 25 °C) with various Piperine analogs (**a**) Piperine (**b**) PIP-1 (**c**) PIP-2 (**d**) PIP-3 (**e**) PIP-4 and (**f**) PIP-2 with CT-duplex DNA. The lower panel represents the integrated heat plot of the isothermal calorimetric titration graph depicted in the upper panel. The solid line in the ITC profile represents the best two-modes binding site fitting model.

Isothermal titration calorimetry (ITC) is a sophisticated technique for determining binding affinity and non-covalent complex thermodynamics. We have employed ITC for the interaction analysis of Piperine and its analogs with the *c-myc* G4 DNA as a secondary screening (Fig. 4a–e). The ITC thermograms were obtained by the titration of Piperine and Piperine analogs with *c-myc* G4 DNA. Each peak area was related to a single injection and corresponded to the heat generated during the injection. To further calculate the heat of dilution, we injected Piperine and Piperine analogs solution in a cell without *c-myc* G4 DNA. The ITC thermogram at the lower panel depicts the corrected heat, which is plotted against the molar ratio (ligand/*c-myc* G4 DNA). The corrected thermograms for Piperine and Piperine analogs with *c-myc* G4 DNA were best fitted in two binding site model as we have also employed to fit the fluorescence binding data. To better understand the interaction between ligands and *c-myc*, different thermodynamic parameters such as association constant (Ka), entropy change (∆S), enthalpy change (∆H), Gibbs free energy change (∆G), and stoichiometry (N) were examined (Supplementary Table S5a). Two forms of binding events were observed when Piperine and its analogs interacted with *c-myc* G4 DNA. First, at a lower saturation level, the binding of Piperine and its analogs with *c-myc* G4 was exothermic. In contrast, in the second event, as increase degree of saturation, the observed binding events were endothermic. During initial injection, the low level of saturation binding event occurs between Piperine and its analogs with *c-myc* G4, which indicates the interaction is enthalpically driven due to van der Waals forces^117^. Afterward, at a higher level of saturation, the endothermic binding events arise, which is entropically favorable and contributed via electrostatic and hydrophobic interaction^118,119^. The negative change in the enthalpy (∆H) for all the ligands-G-quadruplex interactions (Piperine ∆H_1_ = − 1.06 × 10^6^ ± 0.06 cal/mol, PIP-1 ∆H_1_ = − 2.67 × 10^5^ ± 0.08 cal/mol, PIP-2 ∆H_1_ = − 1.38 × 10^4^ ± 0.02 cal/mol, PIP-3 ∆H_1_ = − 1.25 × 10^5^ ± 0.3 cal/mol, PIP-4 ∆H_1_ = − 1.98 × 10^5^ ± 0.2 cal/mol) **(**Supplementary Table S5a) indicated the feasible reaction^120^. The negative change in free energy (∆G) throughout the reactions is attributed to its spontaneity and thermodynamically feasibility^53^. Furthermore, the evaluated association constant obtained for Piperine and Piperine analogs with *c-myc* G4 DNA were in the order of PIP-2 (K_a_^1^ = 3.81 × 10^7^ ± 0.01 M^−1^) > Piperine (K_a_^1^ = 4.56 × 10^5^ ± 0.03 M^−1^) > PIP-1 (K_a_^1^ = 3.34 × 10^5^ ± 0.4 M^−1^) > PIP-4 (K_a_^1^ = 2.46 × 10^5^ ± 0.2 M^−1^) > PIP-3 (K_a_^1^ = 1.10 × 10^5^ ± 0.01 M^−1^) (Supplementary Table S5a). Moreover, negative exothermic peaks pattern in ITC thermograph indicates the non-covalent interaction of Piperine and its analogs with *c-myc* G4 DNA. Among all the Piperine analogs, PIP-2 exhibits the highest association constant on interaction with *c-myc* G4 DNA, even higher than the parent molecule, Piperine. Several pioneering studies have stated that a higher association constant strongly relates to the high affinity between the small molecule and its target^121^. Apart of that, the negative change in enthalpy due to PIP-2 and *c-myc* G4 DNA interaction suggested a favorable thermodynamic reaction with strong affinity and conformational changes. Besides, we have also performed the ITC analysis of PIP-2 analog with CT duplex DNA (K_a_^1^ = 1.36 × 10^3^ ± 0.02 M^−1^), which showed ~ 10^4^ fold lower binding affinity than *c-myc* G4 DNA (Fig. 4f and Supplementary Table S5b). Furthermore, the binding affinity of PIP-2 with *c-myc* duplex and *c-myc* mutant G4 DNA was also examined, and it was observed that PIP-2 exhibits ~ 10^4^ fold higher affinity with the *c-myc* G4 DNA than both these controls (Supplementary Fig. S7 and Table S5b).

The binding data from both fluorescence and ITC experiment indicate that *c-myc* G4 DNA contains two binding sites with a single DNA motif via 1:2 binding ratio. The obtained K_d_^1^ represents the preferential binding site. On the other hand, K_d_^2^ represents the secondary binding site. Hence, the results obtained from the secondary screening through ITC encourage us to proceed further to access the specificity of these potential lead Piperine analogs with *c-myc* G4 DNA.

### Study of interaction of Piperine analog PIP-2 with *c-myc* G4 DNA using fluorescence emission spectroscopy

Our preliminary screening found that Piperine analog PIP-2 showed a higher binding affinity with *c-myc* G4 DNA. Therefore, to further insights the interaction of PIP-2 with *c-myc* DNA were evaluated by using fluorescence emission spectroscopy. The concentration of Piperine analogs PIP-2 (5 µM) was kept constant throughout the experiment. The highest emission peak of Piperine analog PIP-2 was observed at 400 nm upon excitation at 350 nm. The incremental addition of *c-myc* G4 DNA causes a significant enhancement in the emission peak (hyperchromic shift) of PIP-2. This increase in the emission spectra indicated the conjugation of PIP-2 with the *c-myc* G4 DNA, thereby highlighting the ligand's strong interaction with the G-quadruplex DNA and indicates the protection of ligand by the quenching effect of a polar solvent (Supplementary Fig. S8)^122,123^. The deactivation of a ligand in its excited state is due to the low rotational energy barrier of the aromatic moiety in water. The interior hydrophobic environments of *c-myc* G4 DNA support to recover of the fluorescence of Piperine analog PIP-2^122,123^. The titration with *c-myc* G-quadruplex DNA (0.5–0.01 equiv.) in the PIP-2 solution enhances the fluorescence intensity ~ 2–4-fold with no shift ensure the strong interaction between PIP-2 and *c-myc* DNA.

### Assessment of structural stability of Piperine analogs and *c-myc* G4 DNA complex using circular dichroism (CD) melting experiment

**Figure 5:**
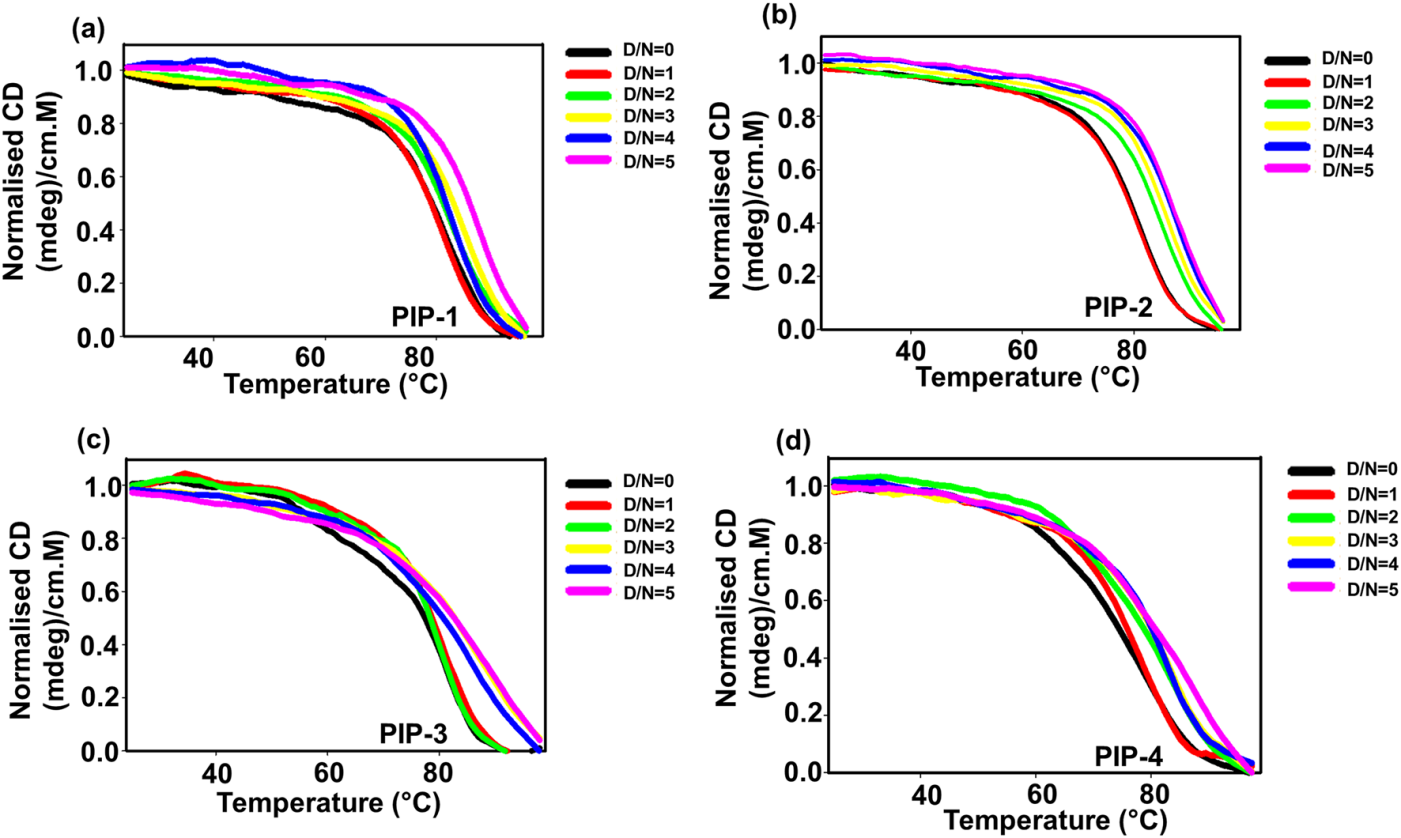
CD melting graph of Piperine analogs with *c-myc* G4 DNA (**a**–**d**) Representative CD melting graphs showing the change in melting temperature of *c-myc* G4 DNA (10 µM) in the absence (D/N = 0) and presence of different Piperine analogs (D/N = 5) (**a**) PIP-1, (**b**) PIP-2, (**c**) PIP-3 and (**d**) PIP-4.

Circular dichroism **(**CD) melting assay were performed to analyze the stabilizing ability of Piperine analogs on the oncogenic promoter of *c-myc* G4 DNA to affirm the role of these analogs in stabilizing the G4 conformation. The stabilization potential of Piperine analogs were observed by monitoring the change in the melting temperature in the absence and presence of ligand. The thermal profile of *c-myc* G4 DNA was obtained at 265 nm, and the resulting sigmoidal curve represented the G4 formation. Initially, in the absence of ligand, the CD melting profile of *c-myc* G4 shows a maximum transition of 76 °C at the D/N ratio of 0.0. On gradual addition of Piperine analogs in 10 µM *c-myc* G4 DNA solutions till D/N ratio 5.0, an increase in the T_m_ of the *c-myc* G4 was observed. Among all the Piperine analogs, PIP-2 showed an increase in the melting temperature (T_m_) up to ~ 14 °C at D/N ratio of 5.0 (Fig. 5b). In contrast, PIP-1, PIP-3, and PIP-4 enhance the transition of melting temperature up to ~ 9 °C, ~ 7 °C and ~ 6 °C, respectively, after interaction with *c-myc* G4 DNA (Fig. 5a,c,d) (Supplementary Fig. S9). Thus, PIP-2 provides better stabilization potential upon interaction with *c-myc* G4 DNA. The melting temperature (T_m_) for the *c-myc-*PIP-2 complex is also higher than the *c-myc*-Piperine complex, highlighting the stronger affinity and stabilizing potential of PIP-2 compared to its parent molecule^45^. The formation of the ligand-*c-myc* G-quadruplex complex increases the stability of the quadruplex attributed due to the non-covalent interaction. The PIP-2 molecule causes a maximum increase in the T_m_ for *c-myc* G4 DNA suggesting the strong stabilization and kinetics upon ligand-quadruplex complex formation. Furthermore, we have also employed CD melting assay to investigate the stabilizing property of PIP-2 with different G4 DNAs such as *bcl-2*, *tel22*, and *c-kit21*, as they contain different sequences and topologies (Supplementary Figs. S9 and S10). The titration was performed by progressive addition PIP-2 in other G4 DNAs solution until the saturation has been achieved (achieved at D/N = 2.0). However, we did not find a significant increase in the melting temperature for these G-quadruplexes DNA upon binding with PIP-2 analog, which indicates the poor binding ability of PIP-2 with other G4 DNA structures (ΔTm ≤ 2.5 °C) (Supplementary Figs. S9 and S10). The highest increase in the stability of *c-myc* G4 DNA upon the interaction of PIP-2 may arise due to the different sequence-specific binding property, topology, conformation, size, and length of the loop compared to other G-quadruplex motifs. Thus, our CD melting results were coherent the fluorescence binding and ITC findings, demonstrating the selectivity of the Piperine analog PIP-2 for *c-myc* G4 DNA with a maximum increase in melting temperature.

### Determination of the secondary conformation of *c-myc* G4 DNA after the formation of a complex with different Piperine analogs

**Figure 6:**
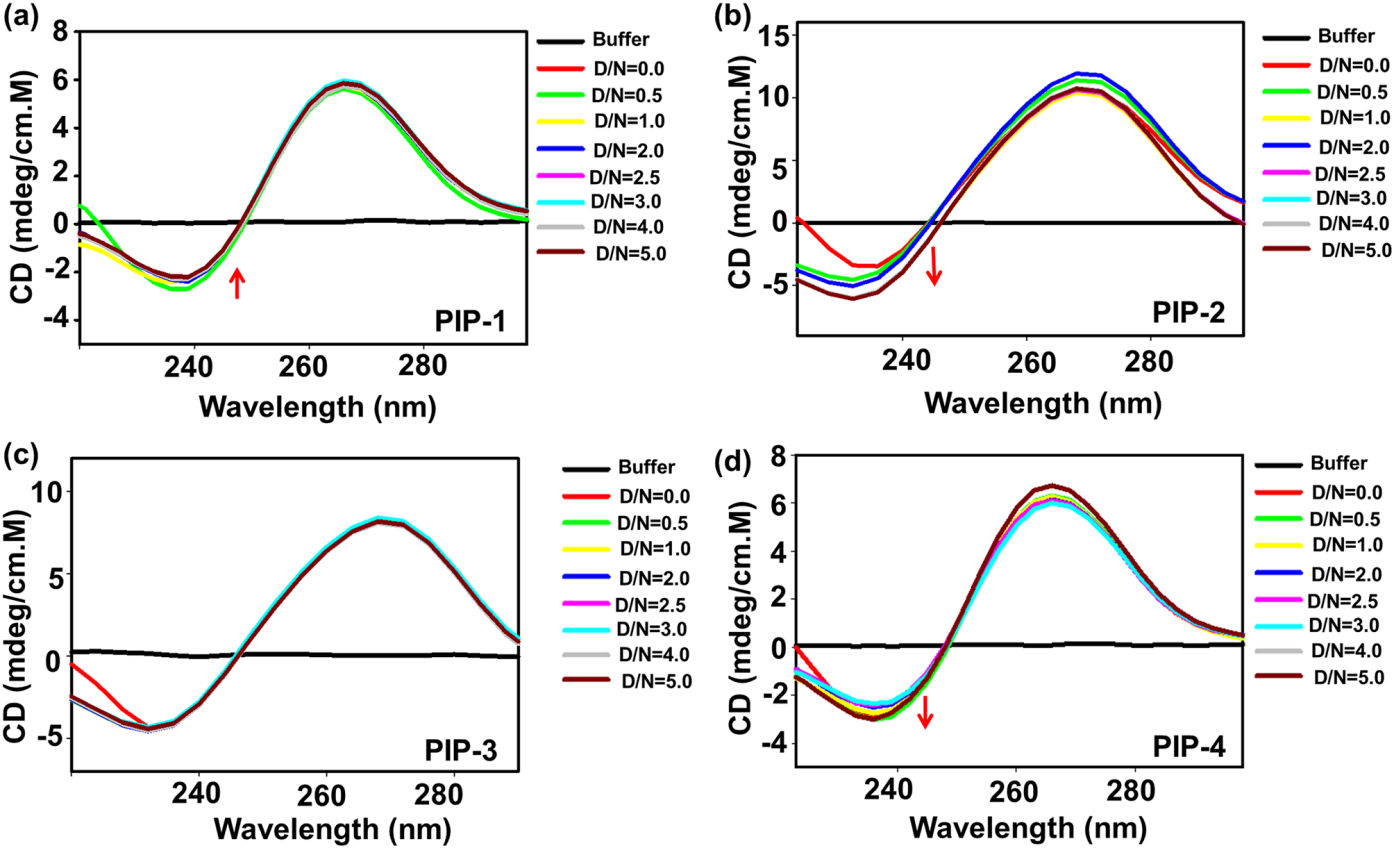
CD spectra profile of *c-myc* G4 DNA with Piperine analogs. CD spectra of free (10 µM) *c-myc* G4 DNA and its complex with successive addition of different Piperine analogs (**a**) PIP-1 (**b**) PIP-2 (**c**) PIP-3, and (**d**) PIP-4.

Circular dichroism (CD) spectroscopy provides information about the change in the conformation of nucleic acid upon ligand interaction. Therefore, we have performed CD spectroscopy to get a clear picture of interaction between lead Piperine analogs (PIP-1, PIP-2, PIP-3 and PIP-4) with *c-myc* G4 DNA. At D/N ratio = 0, the CD spectrum of *c-myc* G4 DNA formed a signature pattern of parallel topology with a negative peak at ~ 240 nm and a positive peak at ~ 265 nm^45^. The successive titration of all the investigated four lead Piperine analogs were performed in 10 µM *c-myc* G4 DNA till D/N = 5.0. The progressive addition of Piperine analogs PIP-1 in *c-myc* G4 DNA caused a decrease in the intensity of the negative peak at ~ 240 nm but did not affect the intensity of the positive peak at ~ 265 nm (Fig. 6a,c). In contrast, the incremental addition of PIP-3 neither affects the intensity of the positive peak nor the negative peak. Similarly, the progressive addition of PIP-2 and PIP-4 analogs cause an increase in the intensity of the negative peak for *c-myc* DNA in a dose-dependent manner (Fig. 6b,d).These findings indicate that Piperine analogs PIP-2 and PIP-4 interact with the *c-myc* G4 DNA structure and stabilize the parallel conformation of *c-myc* G4 DNA without disrupting its DNA structure, and the aromatic groups of these analogs can interact with the *c-myc* DNA bases via stacking mode^124,125^. On the other hand, PIP-1 and PIP-3 interact with the *c-myc* G4 DNA through other modes of binding. Hence, all the Piperine analogs sustain *c-myc* G4 DNA conformation without affecting its topology upon interaction.

### Gel retardation and PCR stop assay of Piperine analogs with *c-myc* G4 DNA

The gel retardation assay is a fast and efficient way to analyze the interaction of small molecules with DNA. Therefore, to study the binding of lead Piperine analogs with *c-myc* G4 DNA, we conducted gel retardation assay by incubating the *c-myc* DNA with different concentrations of Piperine analogs (0.00–100.00 µM) for half an hour and then resolved on 15% native PAGE. We have observed the significant retardation in the band mobility by increasing the concentration of Piperine analogs that strongly support the *c-myc*-Piperine analogs complex. On the other hand, low ligand concentration enhances the migration rate of drug-quadruplex adduct due to weak interaction. In our results, the highest migration rate was observed in the last lane with no ligand (control) (Supplementary Fig. S11 and see full gel images Fig. S20). Owing to the fact that the compounds are mostly small molecules with low molecular weight, movement is usually slower compared to observe for high molecular weight proteins^126^. Nevertheless, PIP-2 exhibited significant and maximum retardation with *c-myc* G4 DNA compared to other analogs (PIP-1, PIP-3, PIP-4), indicating the strong interaction of PIP-2 with the *c-myc* G4 DNA. Furthermore, we have also performed the gel retardation assay of PIP-2 analog with mutant *c-myc* G4 sequence that is unable to form G4 structure, but no significant band shift was observed (Supplementary Fig. S13a and see full gel images Fig. S22a).

Next, we have performed a PCR stop assay to analyze whether the interaction of Piperine analogs induced stabilization in the *c-myc* G4 structure can impede the movement of DNA polymerase or not. The *c-myc* G4 DNA and their respective primers were incubated with different concentrations of Piperine analogs (PIP-1, PIP-2, PIP-3, and PIP-4) for 30 min, and PCR reactions were performed. The resulting PCR products were analyzed on 3% agarose gel. In PCR stop assay, we observed that the increase in the concentration of Piperine analogs from 0.00 to 100.00 µM leads to a decrease in the intensity of the PCR product due to the hindrance in the *Taq* polymerase activity (Supplementary Fig. S12 and see full gel images in Supplementary Fig. S21). The highest band intensity was observed in the control lane with no ligand (control). Hence, the results indicate that Piperine analogs induced the stabilization in the *c-myc* G4 structure that paused the *Taq* polymerase amplification activity and decreased the amplification of the final PCR product.

Moreover, we have also employed the PCR stop assay of PIP-2 with *c-myc* mutant G4 DNA. There was no noticeable difference in the intensity of their PCR product (Supplementary Fig. S13b and see full gel images in Fig. S22b). Hence, these results were indicating that PIP-2 stabilizes the G-quadruplex motif present in the promoter region of the *c-myc* oncogene.

In conclusion, the biophysical analysis demonstrated that PIP-2 showed strong binding affinity, selectivity, and stabilizing potential for *c-myc* G4 DNA over other Piperine analogs. Thus, these results strengthen the notion to select PIP-2 as a lead molecule and encouraged us to perform further molecular dynamic and cellular based studies.

### Study of the atomic level interaction of *c-myc*-PIP-2 G4 DNA complex by molecular dynamics simulation

The plausible binding modes of Piperine analogs with *c-myc* G4 DNA were investigated by using computational methods. According to our fluorescence binding results, all the Piperine analogs and *c-myc* G4 DNA binding were best fitted in two-mode was 1:2; therefore, we docked the Piperine analogs with *c-myc* G4 (PDB code: 2A5R)^86^ up to 1:2 ratio. In our results, we found that the Piperine analogs interact with the *c-myc* G4 DNA through stacking or the groove mode of interaction. The PIP-1, PIP-5, PIP-6, PIP-7, PIP-8, PIP-9, PIP-10, and PIP-12 bind with the *c-myc* DNA via groove mode. In contrast, PIP-2, PIP-3, PIP-4, and PIP-11 interact through both stacking and groove mode (Supplementary Table S6 and Fig. S14**)**. In our results, we found that PIP-2 analog exhibited minimum binding energy upon interaction with *c-myc* G4 DNA (Supplementary Table S6). Next, we have also carried out the molecular docking of PIP-2 with other G-quadruplex forming DNA such as *bcl-2* (PDB code: 2F8U)^88^, *c-kit* (PDB code: 2KYP)^90^, and telomeric DNA (PDB code: 4G0F)^89^. We observed that PIP-2 interacts with *bcl-2* and *tel22* at their groove site while bind with *c-kit* through stacking and groove mode (Supplementary Fig. S15). However, we have obtained the minimum binding energy for PIP-2-*c-myc* G4 DNA complex indicates the strong interaction over other Piperine analogs and other G4 DNA (Supplementary Fig. S15). Hence, the obtained results harmonize with our previous biophysical results.

**Figure 7:**
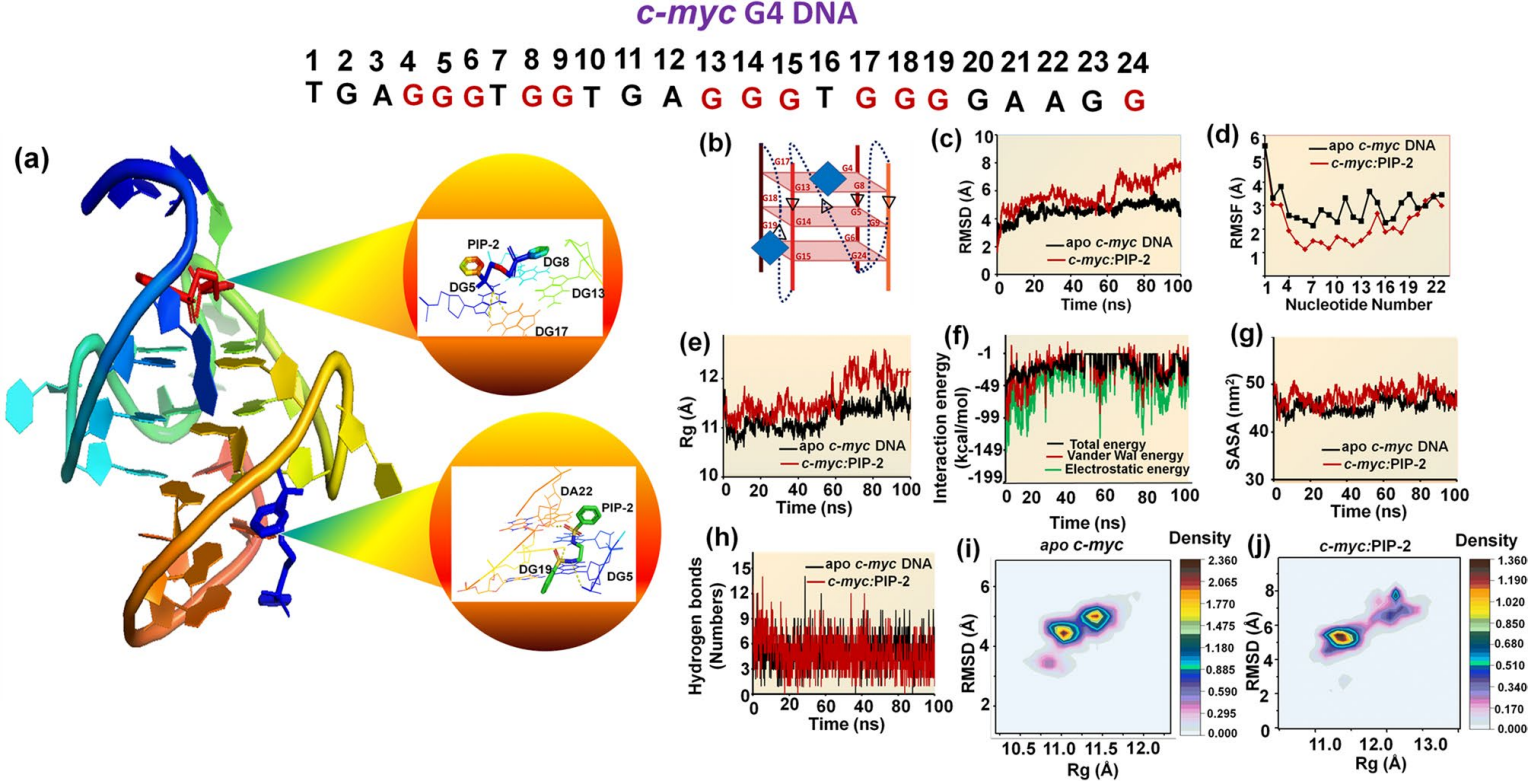
Molecular docking and simulation analysis of *c-myc* G4 DNA with Piperine analog PIP-2 (**a**–**c**). (**a**) Docked PIP-2 with the G-quadruplex structure formed in the *c-myc* promoter region. One PIP-2 molecule interacts with the *c-myc* G4 DNA by stacking on the upper tetrad of (G4–G8–G13–G17) and while the second molecule interacts with the groove region of G-quadruplex DNA. Inset shows the enlarged image of molecular interaction between the PIP-2 and the *c-myc* G4 DNA. (**b**) Schematic representation of the docking between PIP-2 (dark blue) and *c-myc* G4 DNA. (**c**) A plot of RMSD of all atoms vs. time for apo *c-myc* G4 DNA, and *c-myc*-PIP-2 DNA complex. (**d**) Root Mean square fluctuation (RMSF) (**e**) Radius of gyration (Rg) (**f**) interaction energy (**g**) Solvent accessibility surface area (SASA) (**h**) Number of hydrogen bond were evaluated for both apo *c-myc* DNA and *c-myc*-PIP-2 complex. (**i**) The graphs represent the 2D PMF free energy landscapes for the apo *c-myc* and (**j**) *c-myc*-PIP-2 complex.

The docking results for PIP-2 and *c-myc* G4 DNA up to 1:2 ratio showed that the PIP-2 molecule interacts with the *c-myc* G4 by forming stacking interaction with G13 and G4 residues of the upper tetrad (G4, G8, G13, and G17) and form hydrogen bond with G17 residue of *c-myc* DNA (Fig. 7a,b and Supplementary Fig. S16a,b). The binding energy obtained from the stacking mode of interaction was to be − 6.07 kcal/mol suggesting the formation of stable complex. Thus, the presence of the aromatic ring in the ligand can efficiently stack on the G-quadrate^127^. The second ligand molecule (PIP-2) was found to interact with the groove region of *c-myc* G4 DNA with the binding energy of − 7.24 kal/mol (Fig. 7a,b). The second docked PIP-2 forms a hydrogen bond with A22 and G20 residue of the loop region in the *c-myc* G4 structure **(**Supplementary Fig. S16c,d).

To further gain insights of the interaction between *c-myc* DNA and PIP-2, we carried out molecular dynamic simulation. Molecular dynamic simulation provides detailed information of PIP-2 and *c-myc* G4 complex stability and to gain more information related to the atomic level for the *c-myc-*PIP-2 complex. MD simulation was performed for 100 ns for apo *c-myc* and *c-myc-*PIP-2 complex. After the equilibration run of 10,000 steps, the model was minimized with the lowest potential energy of − 57,100 kcal/mol. In the RMSD plot, an initial jump was obtained for the first 5 ns for both apo *c-myc* and *c-myc-*PIP-2 complex due to the model’s relaxation compared to the starting frame. The trajectory analysis of *c-myc-*PIP-2 complex showed stability with a minimum fluctuation range from 3 to 5 Å (Fig. 7c). After the initial increase, the RMSD plot of the *c-myc-*PIP-2 complex was found to be stable throughout the simulation that shows the stabilizing effect of PIP-2 on the *c-myc* G4 structure **(**Supplementary Table S7). The Root Mean Square Fluctuation (RMSF) of the apo *c-myc* and *c-myc*-PIP-2 complex indicates that the complex was more stable during the entire simulation as less fluctuations were observed in the complex compared to the apo *c-myc* G4 DNA (Fig. 7d). The radius of gyration (Rg) provides information related to the compactness of the system and the stability of the model structure. The Rg plot of the *c-myc-*PIP-2 complex showed minimum fluctuation over apo *c-myc* G4 DNA and further affirmed the stability of the *c-myc-*PIP-2 complex throughout the simulation (Fig. 7e). We have also calculated the interaction energy of PIP-2 and *c-myc* G4 DNA complex by using NAMD VMD plug in and found in the range of − 5 to − 150 kcal/mol (Fig. 7f). Next, we have evaluated the hydrophobic core region of apo *c-myc* and *c-myc*-PIP-2 complex by calculating the solvent accessibility surface area (SASA). Average SASA values of apo *c-myc* and *c-myc-*PIP-2 complex were calculated as 45.69 nm^2^ and 46.66 nm^2^, respectively which were constant throughout the simulation process (Fig. 7g and Supplementary Table S7). Hydrogen bond formed between the ligand and the macromolecule can be used to study an interaction’s stability, specificity, and directionality^128^. To investigate the stability of *c-myc* DNA and PIP-2, we have analyzed the intramolecular hydrogen bond formed in *c-myc* DNA before and after the binding of the PIP-2 analog to evaluate the stability of the *c-myc*-PIP-2 complex during the simulation. The average intramolecular hydrogen bond within *c-myc* DNA before and after PIP-2 binding was found to be a 4 and 9.5, respectively, indicating the stability of the complex (Fig. 7h and Supplementary Table S7). Furthermore, the different conformers structure generated for apo *c-myc* and *c-myc*-PIP-2 complex was set as 1 to understand the conformational changes that occur in *c-myc* DNA at an atomic level. The 2D free energy landscape of apo *c-myc* and *c-myc*-PIP-2 complex were generated by using the Rg and RMSD in reaction coordinates and implemented the Potential Motive force (PMF). It showed the different conformation of apo *c-myc* and *c-myc*-PIP-2 complex. The conformer distribution from apo *c-myc* is positioned at RMSDs of 2–6 Å and Rgs of 10–12 Å, whereas *c-myc*-PIP-2 is found at RMSDs of 2–9 Å and Rgs of 10–13 Å, showing that *c-myc*-PIP-2 samples more flexible conformers than apo *c-myc* (Fig. 7i,j). Regarding the stability of *c-myc*-PIP-2 complex, the free energy surface landscape contour plots show that *c-myc* DNA was stable upon interaction with PIP-2 and the range of the dark brown basins was smaller compared to apo *c-myc* (Supplementary Fig. S17a,b). This shows that the *c-myc* G4 DNA structure is slightly more stable upon interaction with PIP-2 simulation and has the lowest Rg and RMSD values compared to apo *c-myc* the simulations. Additionally, in the *c-myc*-PIP-2 simulation, the deeper valley (ΔG = 0) was observed in the center around 0.5 nm and 1.14 nm, representing that the most stable conformation of the *c-myc*-PIP-2 complex was reported at the RMSD and Rg value of 0.5 and 1.14 nm, respectively (Supplementary Fig. S17a,b).

Moreover, we observed the brown and orange basins of the free energy surface landscapes graph of apo *c-myc* extended more in both Rg and RMSD axis demonstrate the higher fluctuations and confirmed the low stability compared to *c-myc*-PIP-2 complex. The apo *c-myc* DNA exhibit a different set of conformation representing the RMSD coverage area within the limit of ~ 7 kcal/mol, whereas the *c-myc-*PIP-2 complex enclosed ~ 4 kcal/mol suggesting that PIP-2 analog induced the structural restrictions (Supplementary Fig. S17a,b). These results showed that maximum conformational change *c-myc* DNA occurs in an unbound form. Therefore, the binding of PIP-2 with *c-myc* G4 DNA generates a stable complex structure. These observations agree with the biophysical results and show that PIP-2 interacts with the *c-myc* G4 DNA, preferably through the stacking mode of binding.

### Cell toxicity assay of PIP-2 in malignant and non-malignant cell lines

**Figure 8:**
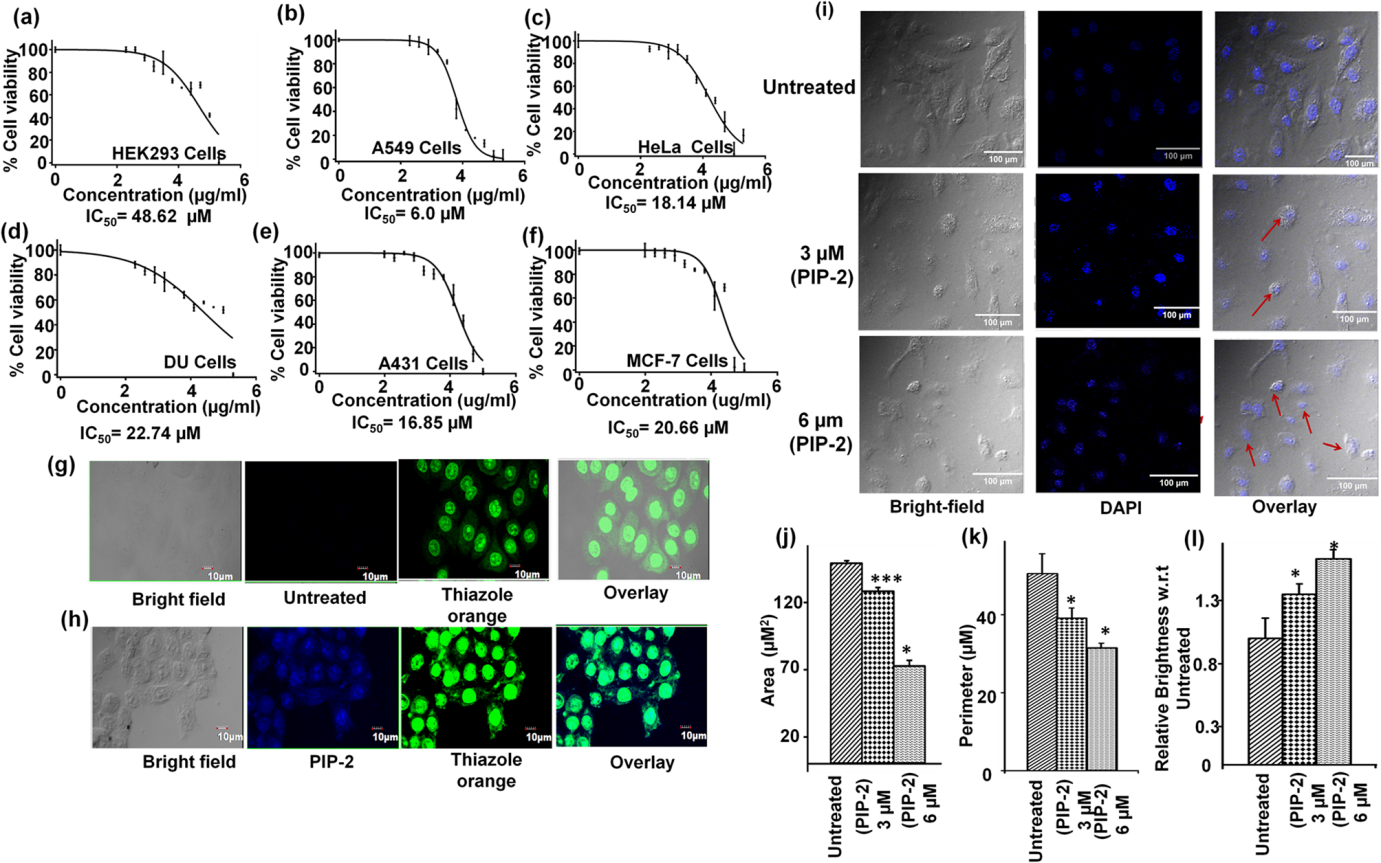
Cell viability assay of PIP-2 with HEK 293 and different cancer cells. (**a**–**f**) The plot represents the cell viability of PIP-2 with (**a**) HEK 293 (**b**) A549, (**c**) HeLa cells, and (**d**) DU cells (**e**) A431 cells (**f)** MCF-7 cells. Co-localization of PIP-2 in A549 cells. (**g**, **h**). Representative confocal images of A549 cancer cells untreated (**g**) and treated (**h**) with PIP-2 (100 µM). Scale bar 10 µm. Apoptosis mediated changes occur upon treatment with PIP-2 (**i**–**k**). (**i**)The morphological changes in the nuclei in cultured A549 cells upon dose-dependent treatment with PIP-2 for 24 h upon staining with DAPI. (**j**, **k**) The quantification analysis of different morphology parameters such as area (**j**), perimeter (**h**), and brightness (**k**). The results are the sum of three sets of experiments, with collected values expressed as means ± standard deviation. The statistical significance determined from the Student's t-test (*P < 0.05, **P < 0.01, ***P < 0.01) by comparing the asterisks indicate the control (untreated) sample. 100 µm scale bar.

The assessment of a compound’s cytotoxicity is required before it can be considered as a potential drug candidate in clinical studies. In order to examine the anti-cancerous activities of Piperine analogs (PIP-1, PIP-2, PIP-3, and PIP-4), MTT experiments were performed with non-malignant HEK 293 and various cancer cell lines such as HeLa (Cervical cancer cells), A549 (Lung adenocarcinoma cell line), DU (Prostate cancer cells), A431 (Skin cancer cells), and MCF-7 (Breast cancer cells) to determine their cytotoxicity (Fig. 8a–f). Both non-malignant and malignant cells were treated with various concentrations of Piperine analogs (with 0.00–200.00 µM) for 24 h. Their IC_50_ (refers to the concentration of the ligand that reduces the cell viability up to 50%) values were determined. The Piperine analogs showed diverse cytotoxicity against different cancer cells. These analogs showed higher toxicity (in 6–30 µM range) with the tumor cells than Piperine^45,129–131^. Interestingly, the ligand PIP-2 exhibits the lowest IC_50_ for A549 cancer cells (6.00 ± 0.12 µM) (Supplementary Table S8) over other cancer cells, indicating strong cytotoxicity towards the lung carcinoma cells. Moreover, PIP-2 exhibits the highest IC_50_ for HEK 293 cells (48.62 ± 0.42 µM) (Supplementary Table S8), thereby showing the lowest toxicity towards the non-cancer cells compared to Piperine. We have also tested the effect of PIP-2 (6 µM at IC_50_ concentration of A549 cells) on different cancer cells by using a qRT-PCR assay to study the expression of *c-myc* gene (Fig. 11d). A normalization procedure was used to determine the fold change in *c-myc* expression in comparison to a housekeeping *β-actin* gene. We observed the highest inhibition for the *c-myc* gene expression in the A549 cells over other tumor cells. Our results showed that the expression of the *c-myc* gene decrease upto ~ 56% with cultured lung cancer cells. These results indicated that PIP-2, a Piperine analog, was more selective for A549 tumor cells than normal and other tumor cells. The reason behind the preference of PIP-2 for A549 cells could be due the selective cellular absorption, which leads to toxicity compared to other tumor cell types^132,133^. These findings demonstrated that PIP-2 analog has the potential to be a potent anti-proliferative agent. As the maximum cytotoxicity was observed in A549 cells, the various cell-based experiments were performed for PIP-2 in A549 cells for further in-depth investigation.

### PIP-2 localizes in the nucleus and initiates cell apoptosis in A549

When it comes to carrying out biological functions, cellular permeabilization and localization are critical variables to consider. Therefore, we investigated the localization of PIP-2 to accumulate in the nucleus (cellular localization) in A549 cells by using confocal laser scanning microscopy. Thiazole orange is a well-known nucleic acid staining dye and stains the entire nucleus at higher concentrations (more than 1 µM). It also acts as an appealing candidate for the staining of non-canonical DNA structures^134^. The A549 cells stained with Thiazole orange without any compound treatment was used as a control (Fig. 8g). As shown in Fig. 8h, the A549 cells were treated with 100 µM concentration of PIP-2 and incubated for 5 h. Then, cells were fixed and mounted with Thiazole orange for the co-staining of the nucleus. The PIP-2 localized in the nucleus and displayed blue fluorescence. The PIP-2 was also found to be present in the cytoplasm of the cell to some extent. The merged green fluorescence (representing Thiazole dye) and blue fluorescence (representing PIP-2) channels, fluorescence microscopy indicates the accumulation of PIP-2 in the nucleus of A549 cells (Fig. 8h overlay).

In order to study the morphological changes of the apoptosis/cell death in the A549 cells treated with PIP-2, the cells for 24 h and was stained with DAPI staining. As shown in Fig. 8i, after the treatment of A549 cells with PIP-2, apoptotic characteristics were observed such as nucleus shrinkage (indicate by red arrow). The fluorescence microscopy analysis showed a decrease in the number of nuclei and a significant increase in the number of apoptotic nuclei induced by PIP-2 in a dose-dependent manner. Moreover, the size and shape of the nuclei were found to be apparently (small and round) different in PIP-2 treated cells compared to control (untreated). The previous scientific reports suggested that the size of the eukaryotic nucleus is in the range of 50–150 μm^2^ ^135,136^. Nuclear parameters such as area, perimeter, and brightness of nuclear DAPI signal were analyzed through Image J software (Fig. 8j–l). The morphology of the nucleus changed and the nucleus size reduced due to the treatment of Piperine analog PIP-2. The increase in the value of nuclear staining intensity in PIP-2 treated A549 cells compared to untreated cells represented the chromosomal DNA condensation. Thus, treatment of PIP-2 in A549 cells induces apoptosis-specific modulation in the morphology of the nucleus and exhibited anti-cancer activities.

### Apoptotic analysis by Hoechst 33342/propidium iodide (PI) and AO/EtBr staining

**Figure 9:**
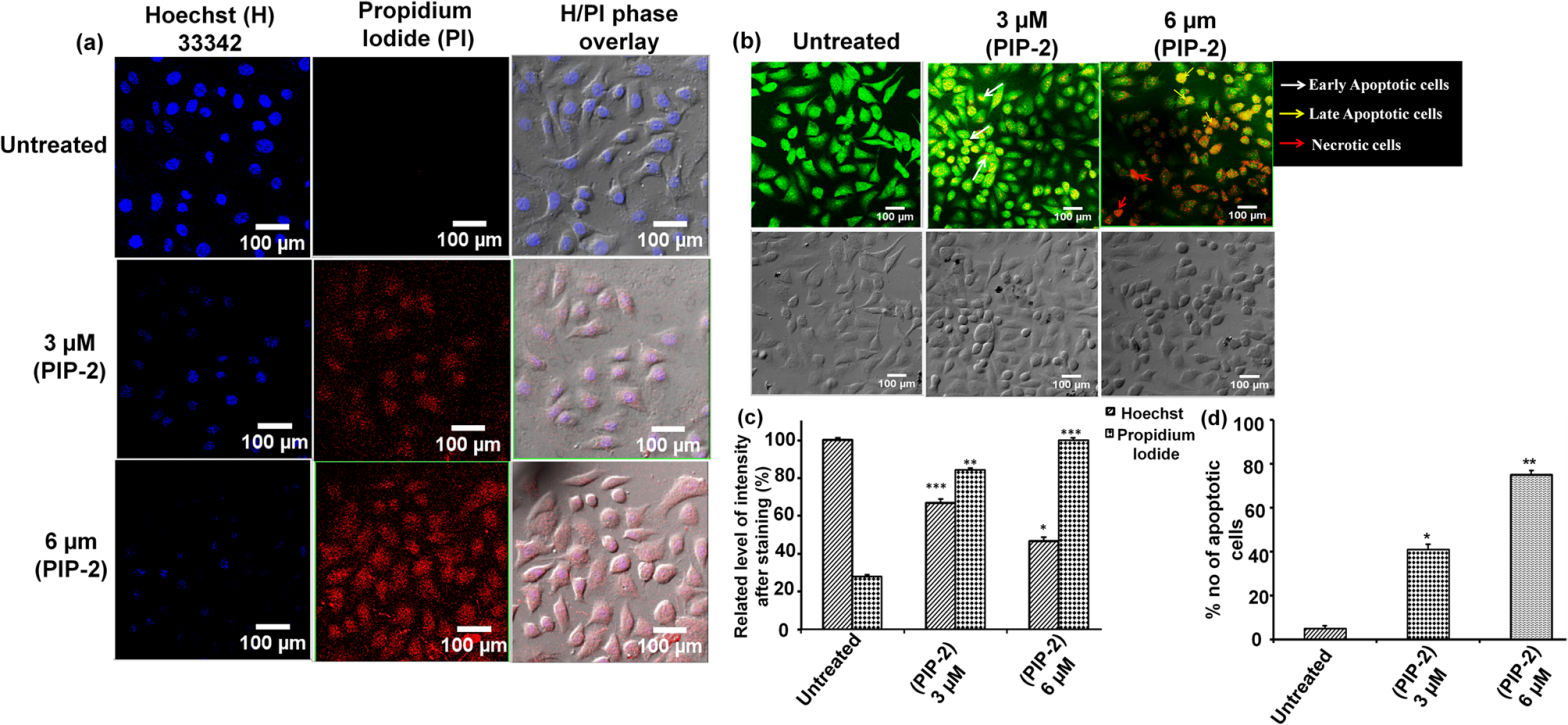
Morphological analysis of A549 cells upon treatment with PIP-2 through different DNA staining dye. (**a**) A549 cells treated with PIP-2 and dual staining was performed with Hoechst 33342/Propidium Iodide (PI). (**b**) The Composite images represent the Acridine orange/Ethidium Bromide (AO/EtBr) stained A549 cells after treatment Piperine analog PIP-2. The white, yellow, and red arrows indicate the early apoptotic, late apoptotic, and necrotic cells, respectively. (**c**, **d**) The bar graph represents the quantitative analysis of H/PI and AO/EtBr dual staining results calculated through Image J. The results are the sum of three separate sets of experiments, with collected values expressed as means ± standard deviation. The statistical significance derived from the Student's t-test (*P < 0.05, **P < 0.01, ***P < 0.001) by analyzing the control (untreated) group is indicated by the asterisks. 100 µm scale bar.

As the PIP-2 treated cells showed apoptotic characteristics in the above experiment, therefore, to further affirmed the anti-proliferative property of PIP-2 by inducing apoptosis, Hoechst 33342/Propidium Iodide **(**PI) and AO/EtBr double staining were performed. Confocal imaging was used to analyze the morphological features to differentiate apoptotic, necrotic, and healthy viable cells. In our results, we have observed that Hoechst staining in the healthy nuclei of the untreated cell had equally distributed chromatin, whereas, on treatment with PIP-2, the healthy cells decreased while the Propidium iodide stain was clearly visible, depicting the presence of apoptotic nuclei (Fig. 9a,c). Furthermore, treatment of A549 cells with a higher concentration of PIP-2 caused an increase in the number of apoptotic cells containing fragmented nuclei that are present in a later stage of apoptosis.

Furthermore, to understand the mode of cell death induced by PIP-2 AO/EtBr, double staining was performed and images were analyzed. The live and healthy untreated A549 cells uptake the Acridine orange and displayed green fluorescence with normal morphology of the nucleus and cytoplasm. On the other hand, cells treated with a low concentration of PIP-2 produced yellowish-green fluorescence, which represented early apoptosis. Moreover, the A549 cells treated with a higher concentration of PIP-2 induced the complete loss of cytoplasmic membrane integrity. As a result, the EtBr enter the cells and displayed orange/red fluorescence and represented the late apoptotic cells (Fig. 9b,d).This shows PIP-2 caused changes in the nuclear morphology, fragmentation of the nuclei, and chromatin condensation that is the characteristic feature of apoptotic cells and follows the apoptosis pathway for cell death. Hence, our results indicated that exposure to Piperine analog PIP-2 significantly triggered apoptosis in A549 cells.

### Cell Scrape and colony formation assay of PIP-2 in A549 cells

**Figure 10:**
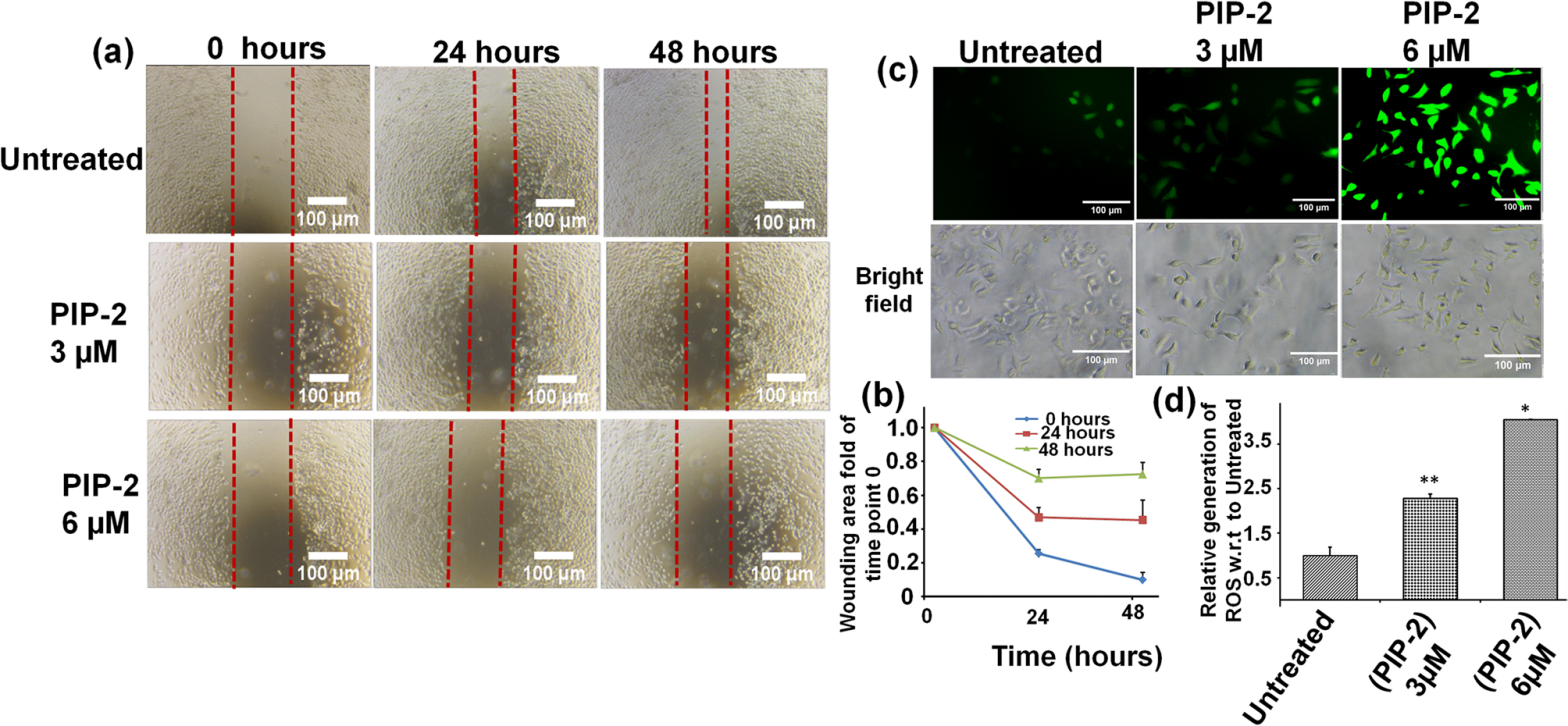
Wound healing assay showed migration speed is decreased in the PIP-2 treated A549 cells. (**a**, **b**) The bright-field images represent the migration speed of A549 cells in both PIP-2 treated and untreated cells. (**b**) The bar graph represents the wound closure area expressed as the area that remained uncovered by the cells. The wound (scratch) area at 0 h time point was set as 1. The results are the average of three different sets of experiments and collected values expressed as means ± SD. (**c**) Reactive oxygen species (ROS) generation assay in A549 cells untreated (control), 3 µM, and 6 µM PIP-2 treatment for 24 h. Images were taken using a fluorescence microscope. Scale bar 100 µm. (**d**) Quantification of ROS in the untreated and PIP-2 treated cells. The statistical significance derived from the Student's t-test (*P < 0.05, **P < 0.01, ***P < 0.001) by analyzing the control (untreated) group is indicated by the asterisks. 100 µm scale bar.

The cell scrape assay was carried out to further validate the inhibitory effect of PIP-2 on the migration of A549 cells. The wound healing mechanism is achieved through several steps such as inflammation, hemostasis, proliferation, and tissue remodeling. The proliferative step is very critical in the malignant cells, and it includes proliferation and migration within the wound^137^. As shown in Fig. 10, PIP-2 inhibited the migration of A549 cells in a dose and time-dependent manner. The wounding area is higher in the PIP-2 treated A549 cells after 24 h and 48 h incubation period due to the inhibition of cell movement.

In contrast, in untreated A549 cells, the wounding area is significantly less due to the fast migration of the cancer cells (Fig. 10a,b). Next, the anti-tumor activity of PIP-2 was also evaluated through colony formation assay to analyze whether the PIP-2 decreased the vitality and tumorigenicity of A549 cells. The formation of the colony decreased upon treatment with PIP-2 after 8 days and was completely inhibited at 6 µM concentration in A549 cells with minimal effect on HEK293 cells (Supplementary Figs. S18 and S19). Thus, these observation were consistent with the MTT results and indicated that PIP-2 could inhibit the invasion, proliferation, and metastasis in A549 tumor cells.

### Measurement of ROS generation in A549 cells upon treatment of PIP-2

It is well known that antitumor active drug generates reactive oxygen species (ROS) that may trigger the mitochondrial pathway for cell apoptosis^138^. Therefore, to investigate the efficacy of PIP-2 in inducing ROS generation, the A549 cells were treated with the PIP-2 analog close to their IC_50_ value (3 µM and 6 µM) and stained with DCFH-DA. The A549 cells exposed to PIP-2 for 24 h, induced a significant increase in ROS generation and showed green fluorescence, while very low level of ROS generation was observed in untreated/control A549 cells relative to the treated cells (Fig. 10c). The ROS generation was more significant at the IC_50_ (6 µM) value of PIP-2, and induced ~ 4 folds more ROS production than the untreated lung cancer cells (Fig. 10d). In cancerous cells, *c-myc* is known for its pivotal role in regulating cell metabolism and energy production. The decrease in the expression level of *c-myc* results in oxidative stress generation^36^. Thus, an increase in the ROS suggests that PIP-2 leads to a decrease in *c-myc* expression, which correlates with the activation of mitochondria-mediated apoptotic cell death by increasing oxidative stress. So, the available data inferred that the PIP-2 analog has the potential anti-cancer activity towards A549 cells.

### mTFP repression using PIP-2 as *c-myc* G4 DNA stabilizer

**Figure 11:**
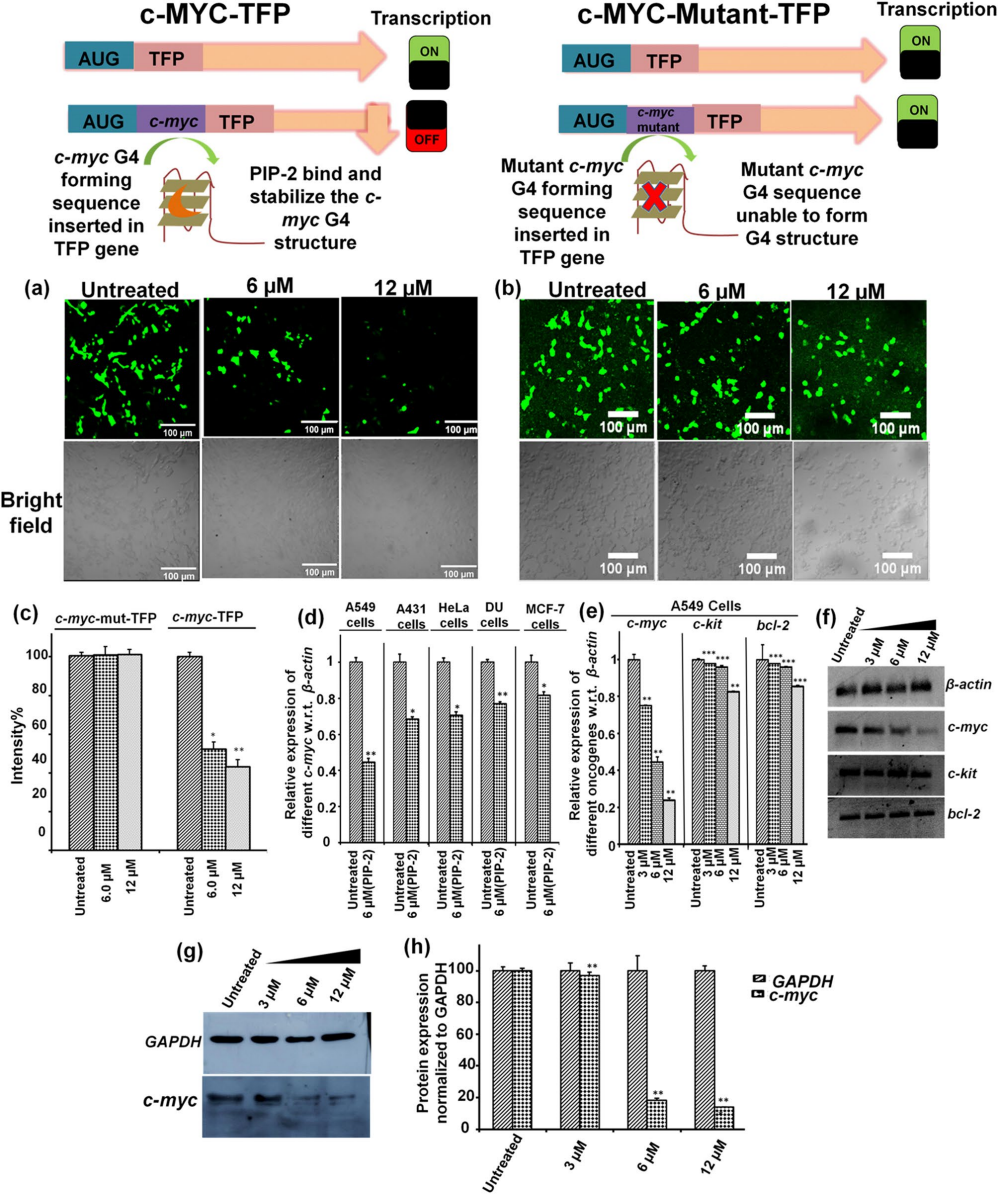
(**a**–**c**) m-TFP based reporter assay of PIP-2 in transfected HEK 293 cells. (**a**) The increase in the concentration of Piperine analog PIP-2 (6 µM and 12 µM) leads to the decrease in the expression of m-TFP in the *c-myc* G-quadruplex-pCAG transfected cells (**b**), while no change was observed in the expression of the *c-myc*-mutant construct, which is unable to form G-quadruplex motif. Scale bar 100 µm (**c**) The bar graph represents the quantitative expression of m-TFP intensity upon the treatment of PIP-2 upon transfection with wild type *c-myc* TFP and *c-myc*-mutant-TFP plasmid HEK293 cells. The untreated group (without ligand) was set as 100%, and error bars reflect the standard deviation of three different sets of experiments. qRT-PCR assay *c-myc* gene expression in different cancer cells and other oncogenes in A549 cells upon treatment of PIP-2 (**d**–**f**). (**d**) The quantitative gene expression study to evaluate the effect of PIP-2 on *c-myc* gene expression in various cancer cells by employing qRT-PCR. (**e**) Bar diagram showing the quantification of *c-myc* and different oncogenes mRNA expression level relative to control by Quantitative Real-time PCR in A549 cells. (**f**) Transcription expression profile of *c-myc*, *c-kit,* and *bcl-2* upon treatment with increasing concentration of PIP-2 resolved in a 3% agarose gel (full-length gel images are provided at Supplementary information Fig. S23a–d. Western blot assay of *c-myc* protein in PIP-2 treated A549 cells (**g**, **h**). (**g**) The western blot represents the effect of PIP-2 on *c-myc* protein in A549 cells in a dose-dependent manner (full-length blot images are provided at Supplementary information Fig. S23e,f. (**h**) The bar graph depicts the quantified expression of *c-myc* protein w.r.t. of *GAPDH* protein upon treatment with PIP-2. The value of the untreated group (without compound) was set as 100%. The results are the sum of three separate sets of experiments, with collected values expressed as means ± standard deviation. The statistical significance derived from the Student's t-test (*P < 0.05, **P < 0.01, ***P < 0.001) by analyzing the control (untreated) group is indicated by the asterisks. 100 µm scale bar.

According to previous scientific reports, formation of a secondary structure on m-RNA affects the translation activity of the gene^139^. We employed mTFP based reporter experiment in which the G-quadruplex forming sequence having 24 base pairs were inserted at the upstream of mTFP gene. The PIP-2 treated cells transfected with *c-myc* G4 plasmid construct showed a decrease in mTFP expression in the dose-dependent manner, while no significant change was observed in the *c-myc*-G4-mutant plasmid construct (Fig. 11a–c). This strengthens the fact that the Piperine analog PIP-2 interacts with the G-quadruplex structure established in the *c-myc* G4 pCAG construct and inhibits downstream mTFP protein expression by obstructing the transcription machinery. Our previous findings reporting the stabilizing impact on *c-myc* and inhibitory effects of PIP-2 in cancer cells were also confirmed by these mTFP reporter-based results.

### Analysis of the effect of PIP-2 on the transcription of *c-myc* and other oncogenes as well as the expression of *c-myc* protein in a dose-dependent manner

Similar to mTFP protein expression, the stabilization of the G4 motif at the *c-myc* promoter region also influence its expression as we discussed earlier (in “Cell toxicity assay of PIP-2 in malignant and non-malignant cell lines”). We have found the maximum decrease in *c-myc* gene expression in A549 cells upon treatment with PIP-2 analog compared to other cancer cells (Fig. 11d). Therefore, we have explored the effect of PIP-2 in different dose-dependent concentrations ½ IC_50_ (3.0 µM) and 2IC_50_ (12.0 µM) in human lung carcinoma cells (A549) to measure the mRNA level of *c-myc* gene after PIP-2 treatment by performing quantitative real-time PCR analysis. The dose-dependent concentration of anti-cancer drugs is used to understand its pharmacokinetics property. Anti-cancer medicines are often given in a broad range of dosages during the first phase of human studies. The understanding of the potential of dose-dependent pharmacokinetics is crucial in the clinical pharmaceutical assessment of novel medicines, and it may even be necessary for the formulation of successful treatment regimens in certain situations^140^. Several anti-cancer medicines have shown dose-dependent pharmacokinetic characteristics^141,142^. Therefore, we studied the different dose-dependent concentration effect of PIP-2 by using the *β-actin* gene to normalize the data obtained from different PIP-2 treated (3.0 µM, 6.0 µM, and 12.0 µM) samples. As mentioned above, our results showed that upon treatment of PIP-2 with different concentrations, a significant reduction in the expression level of the *c-myc* gene (~ 56% relative to control at respective IC_50_ concentration) was observed relative to the housekeeping *β-actin* gene (Fig. 11e,f and see full gel image in Supplementary Fig. S23a,b). Furthermore, an increase in the dose up to 12 µM caused a ~ 77% reduction in the mRNA expression relative to control. Hence, our data depicted the potential negative effect of the PIP-2 on the expression of the *c-myc* oncogene. Moreover, this anti-proliferative property of PIP-2 prompted us to investigate its effects on other oncogenes with G4 motifs, such as *c-kit* and *bcl-2* (Fig. 11e,f see full gel image in Supplementary Fig. S23c,d**)**. However, we did not observe any significant change in the expression of *c-kit* and *bcl-2* oncogenes which suggested that PIP-2 selectively downregulates the expression of *c-myc* gene. Hence, the integration of mTFP reporter and qRT-PCR assay suggested that PIP-2 inhibits the *c-myc* gene's transcription via interfering with the transcription process by stabilizing the *c-myc* G4 motif and preventing RNA polymerase or other transcription factors from being incorporated into the promoter region.

MYC proteins are the transcription factors that regulate the expression of a myriad of proliferative genes. The overexpression of the *c-myc* protein is associated with B cell malignancies and histone transformation^143,144^. In parallel, western blot analysis was also performed, using anti-*c-myc*-antibody to confirm that the reduction in the expression of mRNA levels lead to a decrease in *c-myc* protein. These experiment results illustrated that PIP-2 treatment decreased *c-myc* protein expression in A549 cancer cells compared to untreated cells (Fig. 11g,h and full blot image Fig. S23e,f**)**. Moreover, the expression of the housekeeping *GAPDH* protein was not affected by the treatment of PIP-2. Hence, the overall evaluation of m-TFP reporter assay, qRT-PCR, and western blot suggested that the PIP-2 could specifically interact and stabilize the *c-myc* G4 motif and downregulates the transcription and translation of the *c-myc* gene in lung cancer cells.

## Conclusion

The presence of G4 structure-forming sequences in the promoter site of various oncogenes makes them attractive drug targets against anti-cancer therapeutics. We used Piperine analogs to evaluate their binding affinity with *c-myc* G-quadruplex DNA by using different spectroscopic and biological approaches. This report suggests that Piperine analog PIP-2 exhibited significant selectivity towards *c-myc* G4 DNA over other G-quadruplexes (*bcl-2*, *tel22,* and *c-kit21*) and duplex DNA. The m-TFP reporter assay, RT-PCR, and western blot analysis revealed that PIP-2 downregulates the transcription and translation machinery of the *c-myc* gene. It is noteworthy to mention that in vitro assay results imply that its anti-tumor activity is related to the accumulation of PIP-2 in the nucleus. Overall, our study will help the scientific community to discover a modulable new Piperine scaffold, which showed a higher binding affinity against G4 structures and act as an interesting anti-cancer agent.

## Supplementary Information

Supplementary Information.
