# Supplementary material for: Piperine analogs arrest c-myc gene leading to downregulation of transcription for targeting cancer
Source: Sci Rep. 2021 Nov 25;11:22909. doi: 10.1038/s41598-021-01529-3 (PMC8617303; doi:10.1038/s41598-021-01529-3)
Supplement: Supplementary file 1 — Supplementary Information. [file 41598_2021_1529_MOESM1_ESM.docx]

**Supplementary Information**

**Piperine analogs arrest *c-myc* gene leading to downregulation of transcription for targeting Cancer**

Nirali Pandya^a^, Amit Kumar^a*^

a.Department of Biosciences and Biomedical Engineering, Indian Institute of Technology Indore, Simrol, Indore 453552, India.

*Correspondence: Amit Kumar, Associate Professor, Department of Biosciences and Biomedical Engineering, Indian Institute of Technology Indore, Simrol, Indore 453552, India Tel: +91-731-2438-771; Fax: +91-731-2438-721; Email: [amitk@iiti.ac.in](mailto:amitk@iiti.ac.in)

| **Table of Contents** | | |
| --- | --- | --- |
| **SI. Supplementary Tables** | | S8-S17 |
| **Supplementary Table S1**. | Primers and oligos used in the study. | S8 |
| **Supplementary Table S2**. | Structure of Piperine and its analogs. | S9-S11 |
| **Supplementary Table S3.** | Excitation and emission wavelength of Piperine and its different Piperine analogs. | S12 |
| **Supplementary Table S4a.** | The dissociation constant [K_d_,(µM)] values of Piperine analogs with *c-myc* G4 DNA. | S13 |
| **Supplementary Table S4b.** | The dissociation constant [K_d_,(µM)] values of Piperine analogs with other G-quadruplex DNA. | S13 |
| **Supplementary Table S4c.** | The dissociation constant [K_d_,(µM)] values of Piperine analogs with CT duplex, *c-myc* duplex, and *c-myc* mutant DNA. | S14 |
| **Supplementary Table S5a.** | The association constant values and thermodynamic parameters of Piperine and its analogs with *c-myc* DNA. | S14 |
| S**upplementary Table S5b**. | The association constant values and thermodynamic parameters of Piperine analog PIP-2 with CT-DNA, *c-myc* duplex and *c-myc* G4 mutant DNA. | S15 |
| **Supplementary Table S6.** | The binding energy was obtained from the interaction between Piperine analogs and *c-myc* G4 DNA via molecular docking study. | S16 |
| **Supplementary Table S7.** | The calculated parameters of apo *c-myc* and *c-myc-*PIP-2 complex were obtained from the trajectory analysis of 100 ns MD simulation run. | S17 |
| **Supplementary Table S8.** | IC_50_ values of Piperine and its analogs with normal HEK 293 cells and different cancer cells. | S18 |
| **SII. Supplementary Figures** | | S19-S41 |
| **Supplementary Figure S1.** | Fluorescence titration curve of lead Piperine analogs with *c-myc* G4 DNA. | S19 |
| **Supplementary Figure S2.** | Fluorescence titration curve of lead Piperine analogs with CT duplex DNA. | S20 |
| **Supplementary Figure S3.** | Fluorescence titration curve of lead Piperine analogs with *c-myc* duplex and mutant DNA. | S21 |
| **Supplementary Figure S4.** | Fluorescence titration curve of Piperine analogs with other G4 DNA (*bcl-2*, *tel22,* and *c-kit-21*) DNA. | S22 |
| **Supplementary Figure S5.** | Fluorescence titration curve of other Piperine analogs with other *c-myc* G4 DNA. | S23 |
| **Supplementary Figure S6.** | The bar diagram of fluorescence binding assay of lead Piperine analogs with *c-myc* G4 mutant and *c-myc* duplex DNA. | S24 |
| **Supplementary Figure S7.** | ITC Binding constant graph of PIP-2 with *c-myc* duplex and *c-myc* G4 mutant DNA. | S25 |
| **Supplementary Figure S8.** | Fluorescence emission spectra of PIP-2 with *c-myc* DNA G4 DNA. | S26 |
| **Supplementary Figure S9.** | CD melting temperature values of *c-myc* G4 DNA in the absence and presence of Piperine analogs. | S27 |
| **Supplementary Figure S10.** | CD melting spectra of lead molecule Piperine analogs with different G-quadruplex DNA (*bcl-2, tel22*, *c-kit21)*. | S28 |
| **Supplementary Figure 11.** | Gel retardation assay of Piperine analogs with *c-myc* G4 DNA. | S29 |
| **Supplementary Figure S12**. | PCR stop assay of Piperine analogs with *c-myc* G4 DNA. | S30 |
| **Supplementary Figure S13**. | Gel Retardation and PCR stop assay of lead molecule PIP-2 with *c-myc* G4 mutant DNA. | S31 |
| **Supplementary Figure S14**. | The binding behaviour of Piperine analogs with *c-myc* G4 DNA was analyzed through a docking study. | S32 |
| **Supplementary Figure S15**. | The binding behaviour of Piperine analogs with *c-kit*, *bcl-2,* and *tel22* DNA was analyzed through a docking study. | S33 |
| **Supplementary Figure S16.** | Interaction analysis of PIP-2 and *c-myc* G4 DNA through docking study represented through hydrogen bonding and hydrophobic interaction. | S34 |
| **Supplementary Figure S17.** | The contour graphs represent the 2D free energy surface landscape on the basis of RMSD and Rg values of (a) apo *c-myc* and (b) *c-myc*-PIP-2 complex. | S35 |
| **Supplementary Figure S18.** | Colony formation assay of PIP-2 with HEK 293 cells. | S36 |
| **Supplementary Figure S19**. | Colony formation assay of PIP-2 with A549 cells (lung cancer cells). | S37 |
| **Supplementary Figure S20.** | Full gel retardation images of lead Piperine analogs with *c-myc* DNA | S38 |
| **Supplementary Figure S21.** | Full PCR stop assay images of lead Piperine analogs with *c-myc* DNA | S39 |
| **Supplementary Figure S22.** | Complete gel retardation and PCR stop image of Piperine analog PIP-2 with *c-myc* G4 mutant DNA. | S40 |
| **Supplementary Figure S23.** | Complete RT-PCR transcript gel and western blot images. | S41 |

**
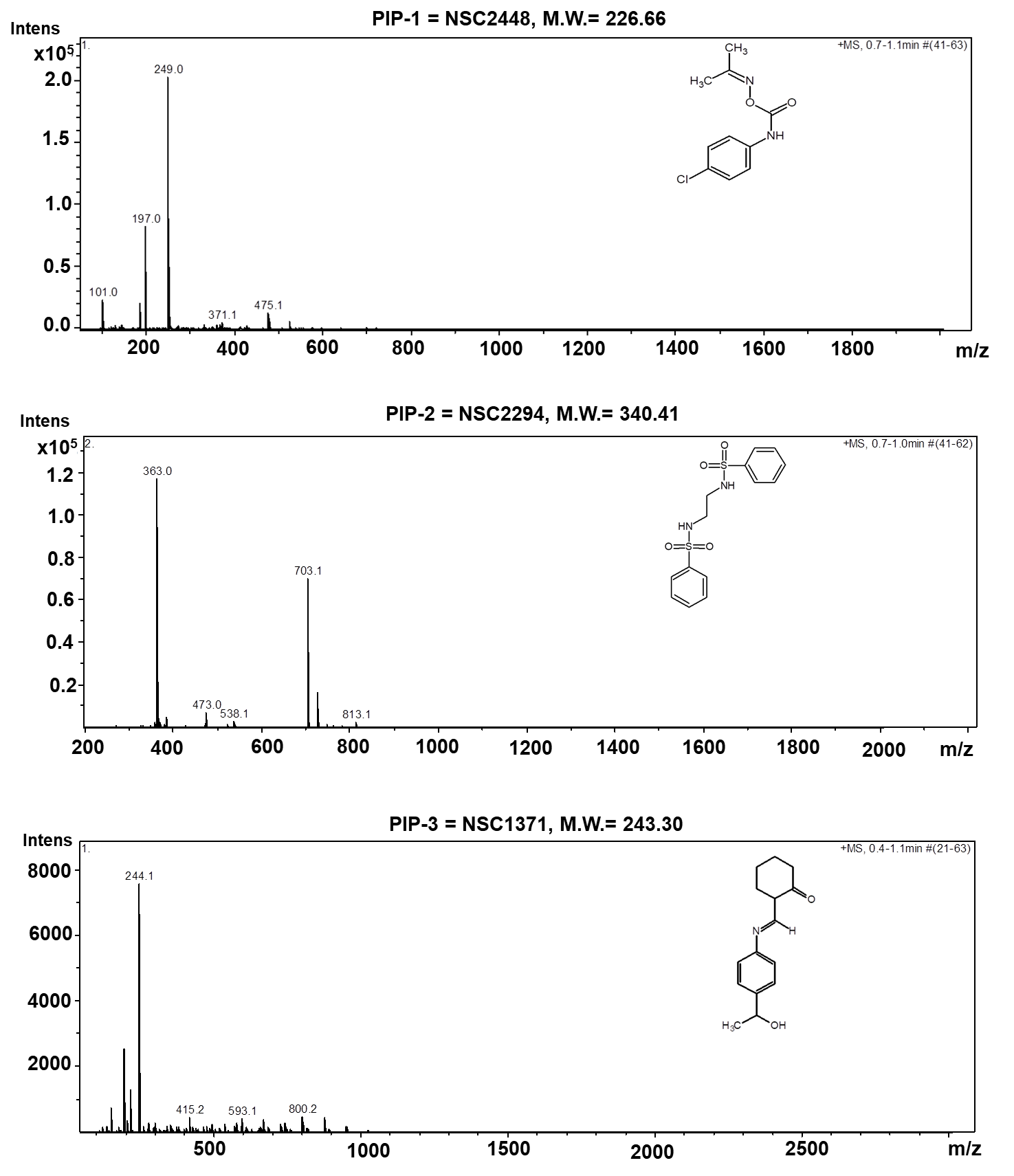
**

**
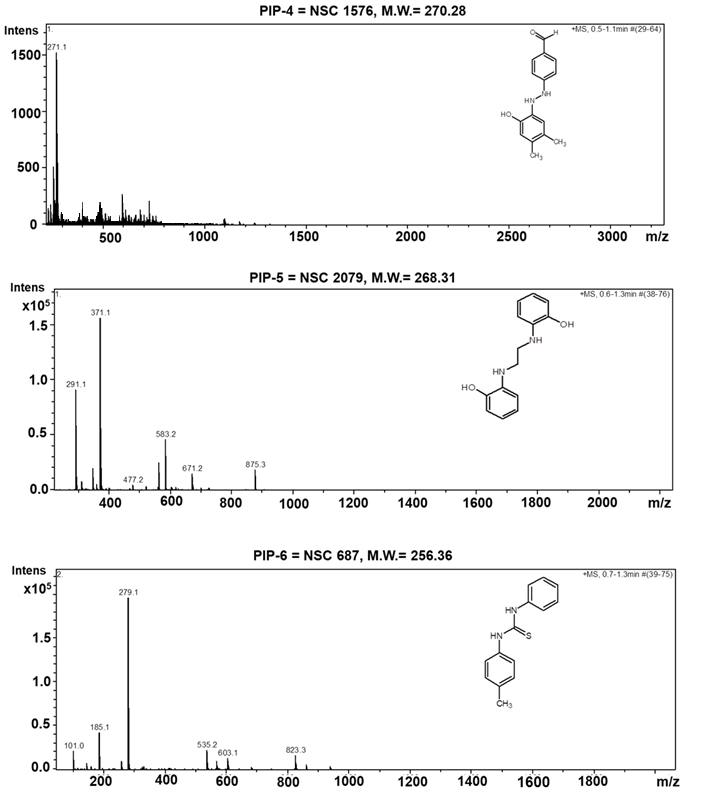
**

**
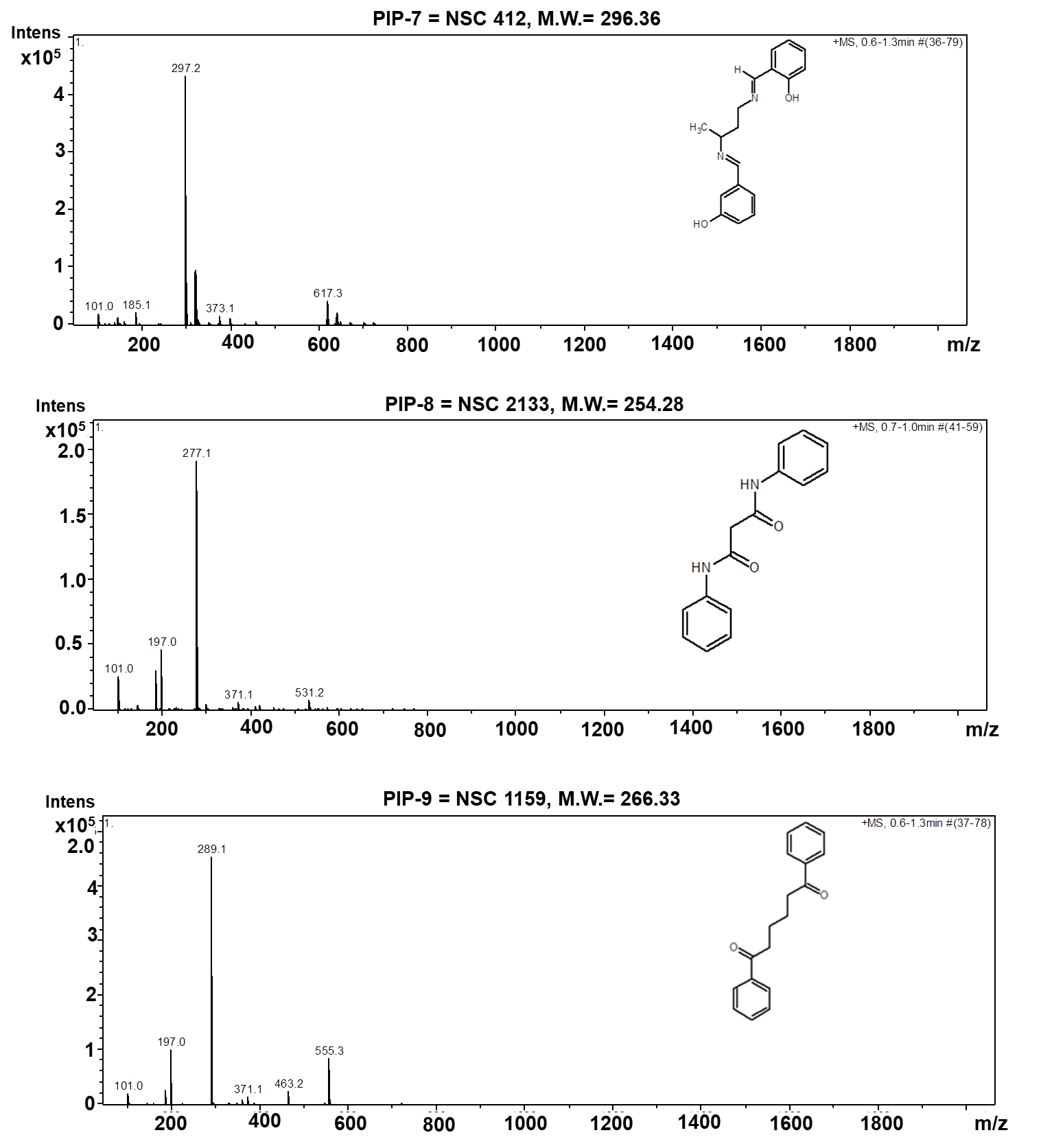
**

**
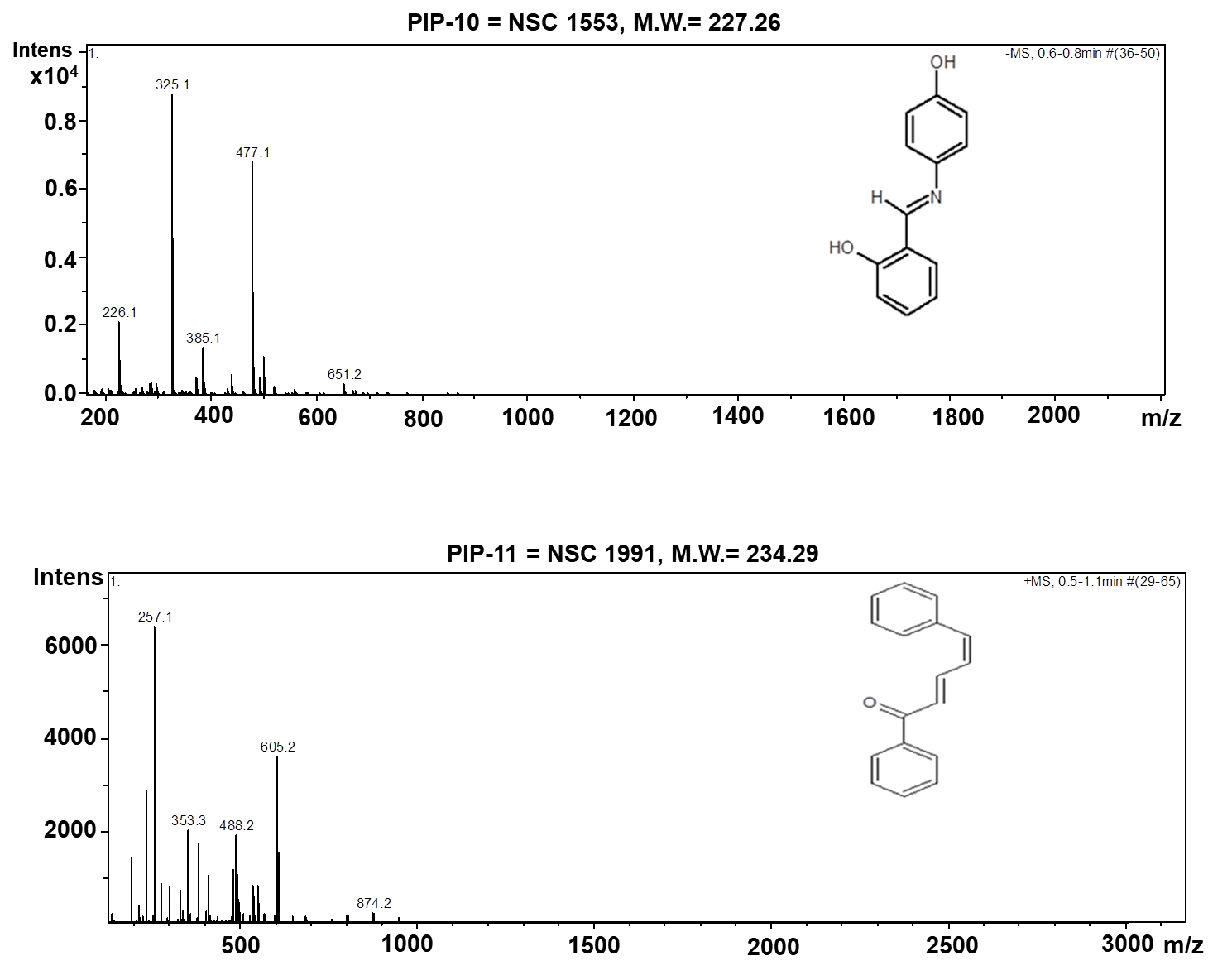
**

**
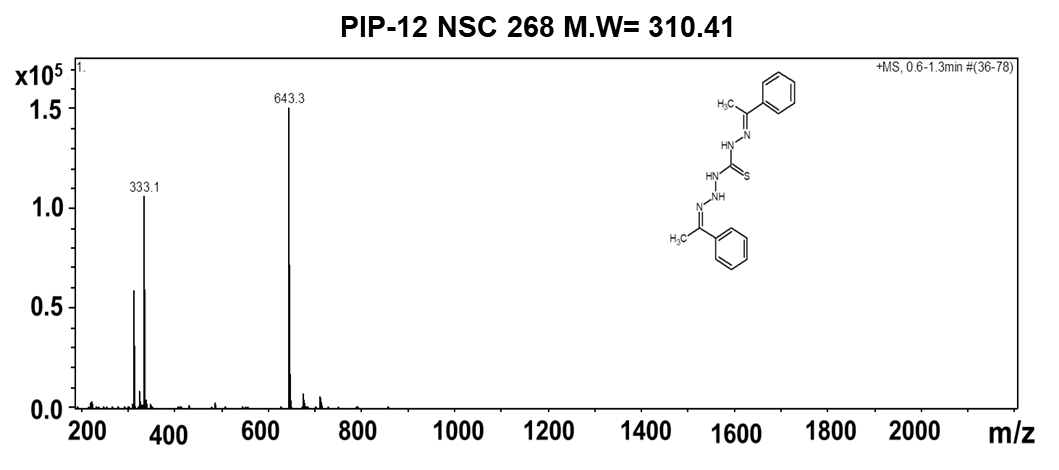
**

**Table S1:** Oligomers andPrimers used in this study

| **S.No** | **Name** | **Sequences (from 5' to 3' )** |
| --- | --- | --- |
| **1** | *c-myc* G-quadruplex | TGAGGGTGGTGAGGGTGGGGAAGG |
| **2** | *tel22* G-quadruplex | AGGGTTAGGGTTAGGGTTAGGG |
| **3** | *c-kit21* G-quadruplex | CGGGCGGGGCGCGAGGAAAA |
| **4** | *bcl-2* G-quadruplex | AGGGGCGGGCGCGGGAGGAAGGGGGCGGGAGCGGGGCTG |
| 6 | *c-myc* (Reverse sequence for duplex) | CCTTCCCCACCCTCACCACCCTCA |
|  | **Primers** | **Sequences (from 5' to 3' )** |
| **7** | *c-myc*-TFP Forward | TGAGGGTGGTGAGGGTGGGGAAGG |
| **8** | *c-myc*-TFP Reverse | CCTTCCCCACCCTCACCACCCTCA |
| **9** | *c-myc*-mut-TFP Forward | TGAAAATAATGAAAATAAAAAAGA |
| **10** | *c-myc*-mut-TFP Reverse | TCTTTTTTATTTTCATTATTTTCA |
| **11** | β-actin (forward): | ATCGTGCGCCCCAGGCAC |
| **12** | β-actin (reverse): | CTCCTTAAT GTC ACG CACGATTTC |
| **13** | *c-myc* Forward | AAGCTGAGGCACACAAAGA |
| **14** | *c-myc* Reverse | GCTTGGACAGGTTAGGAGTAAA |
| **15** | *bcl-2* Forward | CATCAGGAAGGCTAGAGTTACC |
| **16** | *bcl-2*  Reverse | CAGACATTCGGAGACCACAC |
| **17** | *c-kit*  Forward | CGTGGAAAAGAGAAAACAGTCA |
| **18** | *c-kit* Reverse | CACCGTGATGCCAGCTATTA |

**Table S2**.The structure and chemical name of Piperine and its analogs.

| **S. No** | **Structure** |  | **Code** | **Chemical Name** |
| --- | --- | --- | --- | --- |
| **1** |  | **Piperine** | Piperine | 5-(1,3-benzodioxol-5-yl)-1-(1-piperidinyl)-1-penta-2,4-dienone |
| **2** | **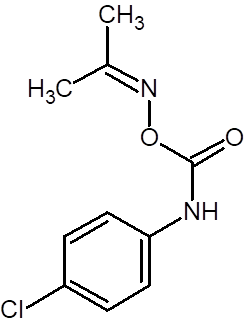** | **PIP-1** | 2448 | 1-chloro-4-(((((1-methylethylidene)amino)oxy)carbonyl)amino)benzene |
| **3** | **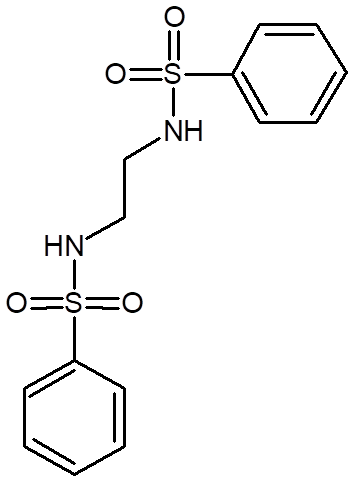** | **PIP-2** | 2294 | N-(2-((phenylsulfonyl)amino)ethyl)benzenesulfonamide |
| **4** |  | **PIP-3** | 1371 | 2-(((4-acetylphenyl)imino)methyl)cyclohexanone |
| **5** | 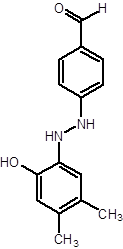 | **PIP-4** | 1576 | 4-((2-hydroxy-4,5-dimethyl)diazenyl)benzoic acid |
| 6 | 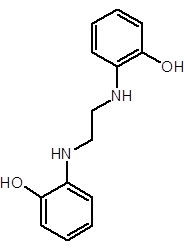 | **PIP-5** | 2079 | 2-(((2-((2-hydroxybenzylidene)amino)ethyl)imino)methyl)phenol |
| **7** | 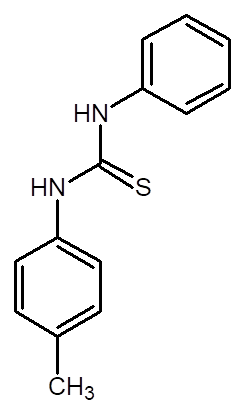 | **PIP-6** | 687 | N,N'-bis(4-methylphenyl)thiourea |
| **8** | **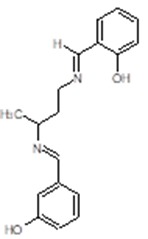** | **PIP-7** | 412 | 2-(((3-((2-hydroxybenzylidene)amino)-1-methylpropyl)imino)methyl)phenol |
| **9** | **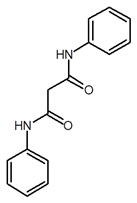** | **PIP-8** | 2133 | N^1^,N^3^-diphenylmalonamide |
| **10** | **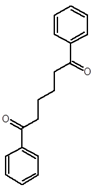** | **PIP-9** | 1159 | 1,6-diphenyl-1,6-hexanedione |
| **11** | **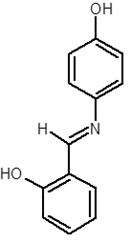** | **PIP-10** | 1553 | 4-((2methoxybenzylidene)amino)phenol |
| **12** | **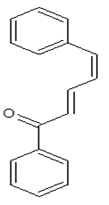** | **PIP-11** | 1991 | 1,5-diphenyl-2,4-pentadien-1-one |
| **13** | 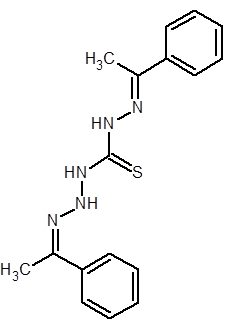 | **PIP-12** | 268 | N'',N'''-bis(1-phenylethylidene)thiocarbonohydrazide |

**Table S3**.The excitation and emission wavelength of Piperine and its analogs.

| **Piperine analogs** | **Excitation (nm)** | **Emission (nm)** |
| --- | --- | --- |
| Piperine | 341 | 486 |
| PIP-1 | 315 | 419 |
| PIP-2 | 347 | 437 |
| PIP-3 | 374 | 437 |
| PIP-4 | 338 | 437 |
| PIP-5 | 345 | 437 |
| PIP-6 | 277 | 437 |
| PIP-7 | 317 | 437 |
| PIP-8 | 347 | 437 |
| PIP-9 | 276 | 523 |
| PIP-10 | 356 | 437 |
| PIP-11 | 347 | 437 |
| PIP-12 | 316 | 437 |

**Table S4a**. The dissociation constant [K_d_,(µM)] values of Piperine analogs with *c-myc* G4 DNA.

| **Compounds** | ***c-myc* G-quadruplex DNA** | |
| --- | --- | --- |
|  | **K_d_^1^ (µM)** | **K_d_^2^ (µM)** |
| **PIP-1** | 0.010±0.003 | 1.61±0.45 |
| **PIP-2** | 0.002±0.001 | 3.82±0.04 |
| **PIP-3** | 0.08±0.007 | 2.65±0.012 |
| **PIP-4** | 0.016±0.005 | 2.07±0.04 |
| **PIP-5** | 2.56±0.02 | 4.32±0.06 |
| **PIP-6** | 1.34±0.13 | 3.41±0.73 |
| **PIP-7** | 3.76±0.03 | 5.61±0.03 |
| **PIP-8** | 4.61±0.05 | 7.23±0.93 |
| **PIP-9** | 2.44±0.02 | 14.30±0.12 |
| **PIP-10** | 1.43±0.06 | 2.78±0.05 |
| **PIP-11** | 1.08±0.01 | ND |
| **PIP-12** | 2.86±0.03 | 3.93±0.23 |

**Table S4b.** The dissociation constant [K_d_, (µM)] values of Piperine analogs with other G-quadruplex DNA.

| **Compound** | ***bcl-2 DNA*** | | ***tel22 DNA*** | | ***c-kit21 DNA*** | |
| --- | --- | --- | --- | --- | --- | --- |
|  | K_d_^1^(µM) | K_d_^2^(µM) | K_d_^1^(µM) | K_d_^2^(µM) | K_d_^1^(µM) | K_d_^2^(µM) |
| **PIP-1** | 0.761±0.01 | 1.21±0.05 | 3.47±0.04 | 2.89±0.03 | 1.71±0.005 | 1.87±0.08 |
| **PIP-2** | 0.024±0.009 | 0.60±0.007 | 0.19±0.02 | 0.21±0.05 | 4.86±0.02 | 5.02±0.03 |
| **PIP-3** | 0.52±0.006 | 0.61±0.005 | 0.28±0.06 | 1.74±0.07 | 1.218±0.05 | 3.36±0.04 |
| **PIP-4** | 1.23±0.002 | 1.74±0.08 | 0.146±0.03 | 5.80±0.021 | 5.34±0.01 | 3.12±0.01 |

**Table S4c.** The dissociation constant [K_d_,(μM)] values of Piperine analogs with CT duplex DNA.

| **Compound** | **CT-DNA** | | ***c-myc* mutant DNA** | | ***c-myc* duplex** | |
| --- | --- | --- | --- | --- | --- | --- |
|  | **K_d_^1^ (µM)** | **K_d_^2^ (µM)** | **K_d_^1^ (µM)** | **K_d_^2^ (µM)** | **K_d_^1^ (µM)** | **K_d_^2^ (µM)** |
| **PIP-1** | 4.47±0.73 | -- | 0.023±0.03 | 0.093±0.42 | 0.559±0.03 | 7.49±0.67 |
| **PIP-2** | 2.02±0.34 | -- | 0.291±0.01 | 0.195±0.04 | 1.81±0.09 | 1.92±0.45 |
| **PIP-3** | 1.17±0.09 | 1.67±0.011 | 0.233±0.04 | 0.135±0.08 | 1.22±0.09 | 1.28±0.05 |
| **PIP-4** | 0.48±0.021 | 0.94±0.07 | 0.187±0.03 | 25.12±0.24 | 1.24±0.10 | 1.36±0.02 |

**Table S5a.**The association constant [K_a_^1^(M^-1^)]values and thermodynamic parameters of Piperine and its analogs with *c-myc* G4 DNA via ITC.

| **Compound** | **K_a_^1^(M^-1^)** | **K_a_^2^(M^-1^)** | **ᐃH_1_ cal/mol** | **ᐃH_2_ cal/mol** | **ᐃS_1_ cal/mol/deg** | **ᐃS_2_ cal/mol/**  **deg** |
| --- | --- | --- | --- | --- | --- | --- |
| **Piperine** | 4.56X10^5^ ±0.03M^-1^ | 4.34X10^5^±0.8M^-1^ | -1.06X10^6^±0.06 | -1.19X10^5^±0.3 | -2.63 X10^3^ | 4.27±0.9 |
| **PIP-1** | 3.34 X10^5^±0.4M^-1^ | 3.12 X10^4^ ±0.1 M^-1^ | -2.67X10^5^±0.08 | -1.14X10^5^±0.5 | 8.70±0.03 | 4.11±0.02 |
| **PIP-2** | 3.81 X10^7^ ±0.01M^-1^ | 6.73 X10^5^±0.03 M^-1^ | -1.38X10^4^±0.02 | -1.16X10^4^±0.06 | -13.9 ±0.5 | -12.2±0.07 |
| **PIP-3** | 1.10X10^5^ ±0.01 M^-1^ | 1.08 X10^5^±0.3 M^-1^ | -1.25X10^5^±0.3 | -- | 23.4±0.2 | 8.45±0.07 |
| **PIP-4** | 2.46 X10^5^±0.2M^-1^ | 2.17 X10^5^ ±0.4 M^-1^ | -1.98X10^5^±0.2 | -1.61X10^5^±0.07 | 6.89±0.05 | 5.19±0.31 |

| **DNA** | **Stoichiometry (Ligand/DNA) N_1_, N_2_** | **ᐃG_1_ (kcal.mol^-1^)** | **ᐃG_2_ (kcal.mol^-1^)** |
| --- | --- | --- | --- |
| **Piperine** | 0.97±0.03, 2.67±0.08 | -0.89±0.02 | -0.86±0.04 |
| **PIP-1** | 2.02±0.12,4.4±0.08 | -0.71±0.06 | -0.81±0.12 |
| **PIP-2** | 1.83±0.06,3.57±0.23 | -0.92±1.34 | -0.24±0.87 |
| **PIP-3** | 2.7±0.05,1.42±0.03 | -0.05±0.01 | -0.041±0.002 |
| **PIP-4** | 2.86±0.02,3.78±0.04 | -0.58±0.05 | -0.45±0.03 |

**Table S5b.**The association constant [K_a_^1^(M^-1^)] values and thermodynamic parameters of lead Piperine analog PIP-2 with duplex DNA.

| **Compound** | **K_a_^1^(M^-1^)** | **K_a_^2^(M^-1^)** | **ᐃH_1_ cal/mol** | **ᐃH_2_ cal/mol** | **ᐃS_1_**  **cal/mol/**  **deg** | **ᐃS_2_**  **cal/mol/**  **deg** |
| --- | --- | --- | --- | --- | --- | --- |
| **CT duplex DNA** | 1.36 X10^3^±0.02M^-1^ | 1.07X10^3^±0.12M^-1^ | -1.89X10^5^±0.05 | -2.43X10^4^±0.17 | 6.50±0.26 | 63.2 |
| ***c-myc* duplex** | 5.34 X10^4^±0.06 M^-1^ | 5.18 X10^4^±0.07 M^-1^ | -5.32±0.03 | -4.86 X10^7^±0.11 | -6.45±0.177 | 1.31 X10^5^ |
| ***c-myc* mutant**  **DNA** | 7.65 X10^3^±0.04 M^-1^ | ND | -4.56X10^4^±0.03 | 2.38X10^4^±3.4 | 7.05±0.12 | 3.42 X10^3^ |

| **DNA** | **Stoichiometry (Ligand/DNA) N_1_, N_2_** | **ᐃG_1_ (kcal.mol^-1^)** | **ᐃG_2_ (kcal.mol^-1^)** |
| --- | --- | --- | --- |
| **CT duplex DNA** | 0.13±0.01, 3.97 ±0.02 | -0.18±0.06 | -0.04±0.005 |
| ***c-myc* duplex** | 1.00±0.01, 2.30 ±0.04 | -0.98±0.05 | -0.91±0.03 |
| ***c-myc* mutant** | 2.31 ±0.02, 1.32 ±0.02 | -1.2±0.01 | - |

**Table S6.** The binding energy value obtained by performing molecular docking study between Piperine analogs with *c-myc* G4 DNA

| **Sr. No** | **Piperine analogs** | **Binding energy (kcal/mol)** |
| --- | --- | --- |
| 1. | PIP-1 | -4.63 |
| 2. | PIP-2 | -7.24 |
| 3. | PIP-3 | 4.73 |
| 4. | PIP-4 | -6.4 |
| 5. | PIP-5 | -3.9 |
| 6. | PIP-6 | -4.19 |
| 7. | PIP-7 | -1.37 |
| 8. | PIP-8 | -1.01 |
| 9. | PIP-9 | -3.67 |
| 10 | PIP-10 | -4.2 |
| 11 | PIP-11 | -4.12 |
| 12 | PIP-12 | -4.15 |

**Table S7.** The calculated parameters of apo *c-myc* and *c-myc-*PIP-2 complex were obtained from the trajectory analysis of 100 ns MD simulation run.

| **Sr. No.** | **Name** | **Average**  **RMSD(Å )** | **Average**  **RMSF(Å )** | **Radius of gyration (Å)** | **Average**  **SASA (nm^2^)** | **Average**  **no. of HB** |
| --- | --- | --- | --- | --- | --- | --- |
| **1.** | ***c-myc* DNA** | 4.5 | 2.9 | 11.21 | 45.69 | 4 |
| **2.** | ***c-myc-*PIP-2 complex** | 5.1 | 2.2 | 11.63 | 46.66 | 9.5 |

| **Cell lines** | **Piperine** | **PIP-1** | **PIP-2** | **PIP-3** | **PIP-4** |
| --- | --- | --- | --- | --- | --- |
| **A549 Cells** | 22.78±0.45 | 27.56±0.34 | 6.00±0.12 | 29.87±1.03 | 27.95±0.89 |
| **HeLa Cells** | 26.56±0.83 | 25.78±0.56 | 18.14±0.39 | 23.98±0.23 | 23.45±0.45 |
| **DU Cells** | 28.33±0.45 | 30.67±0.43 | 22.74±0.64 | 26.45±1.34 | 25.93±0.56 |
| **A431 Cells** | 22.44±0.13 | 18.56±1.6 | 16.85±0.9 | 23.32±1.7 | 21.56±1.41 |
| **MCF-7 Cells** | 23.66±2.1 | 21.64±1.40 | 20.66±1.5 | 24.78±1.2 | 20.98±2.42 |
| **HEK293** | 32.67±0.71 | 42.34±0.78 | 48.62±0.42 | 30.65±2.87 | 27.45±0.67 |

**Table S8.** IC_50_ values for the effect of Piperine analog PIP-2 with different cancerous cells such as A549 ((human lung carcinoma), HeLa (cervical cancer cells), DU (human prostate cancer cell), and normal HEK293 (human embryonic kidney cells) by using MTT assay. Three individual sets of experiments were performed, and collected values were expressed as means ± SD.


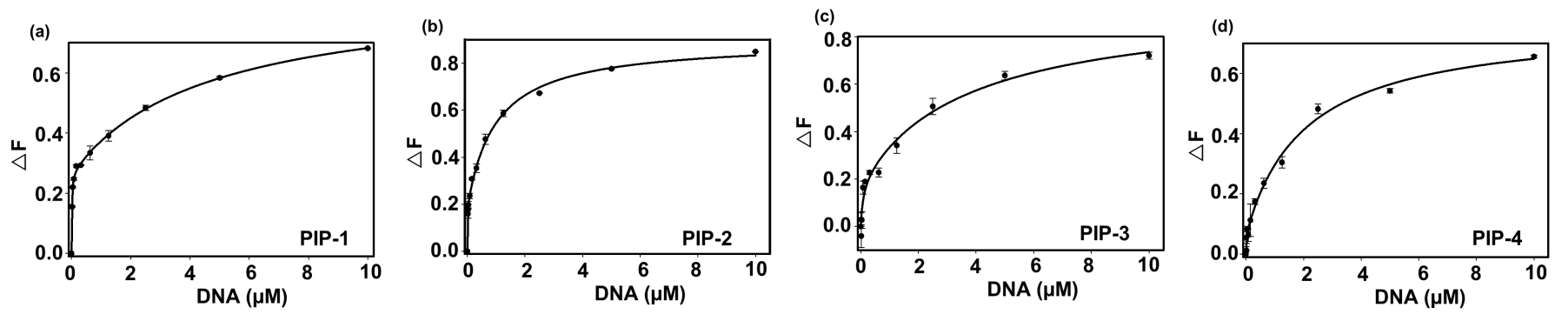


**Figure S1.** Fluorescence titration graph of Piperine analogs (a) PIP-1 (b) PIP-2 (c) PIP-3 and (d) PIP-4 with *c-myc* G4 DNA.The solid black line represents the fitting of the data on two-mode binding saturation mode.

**
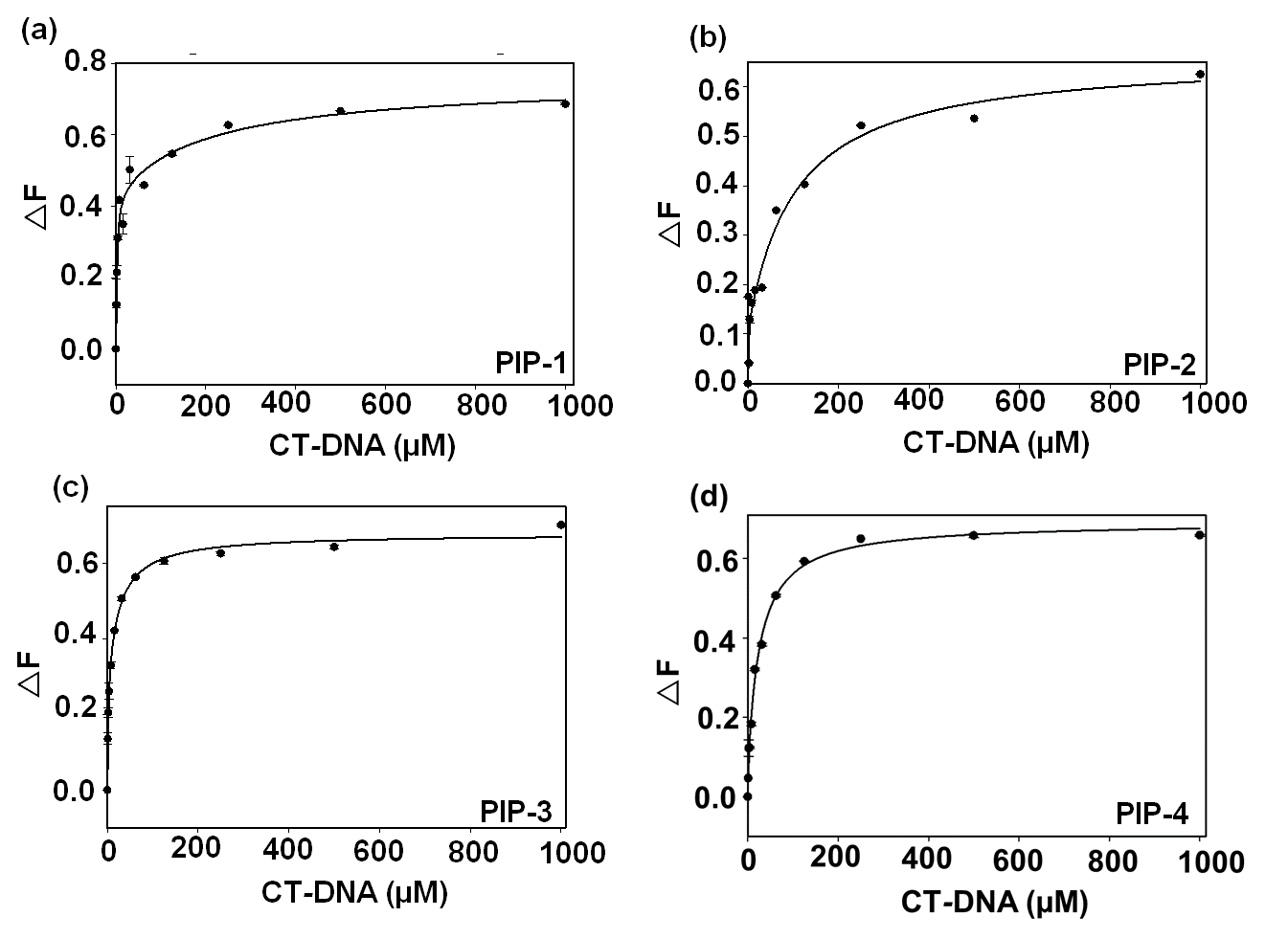
**

**Figure S2.** Fluorescence titration graph of Piperine analogs **(a)** PIP-1 **(b)** PIP-2 **(c)** PIP-3 and **(d)** PIP-4 with CT duplex DNA. The solid black line represents the fitting of the data on two-mode binding saturation mode.


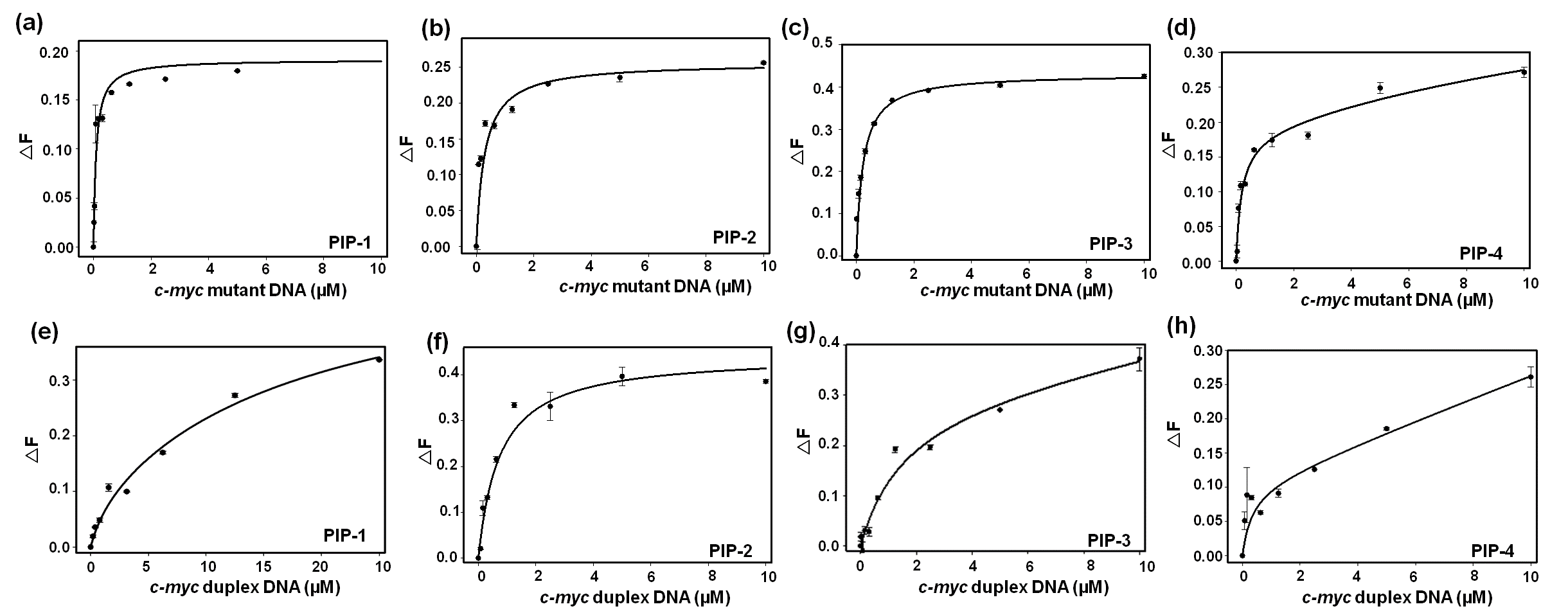


**Figure S3.** Fluorescence titration graph of lead Piperine analogs with *c-myc* G4 mutant and *c-myc* duplex DNA. The solid black line represents the fitting of the data on two-mode binding saturation mode.


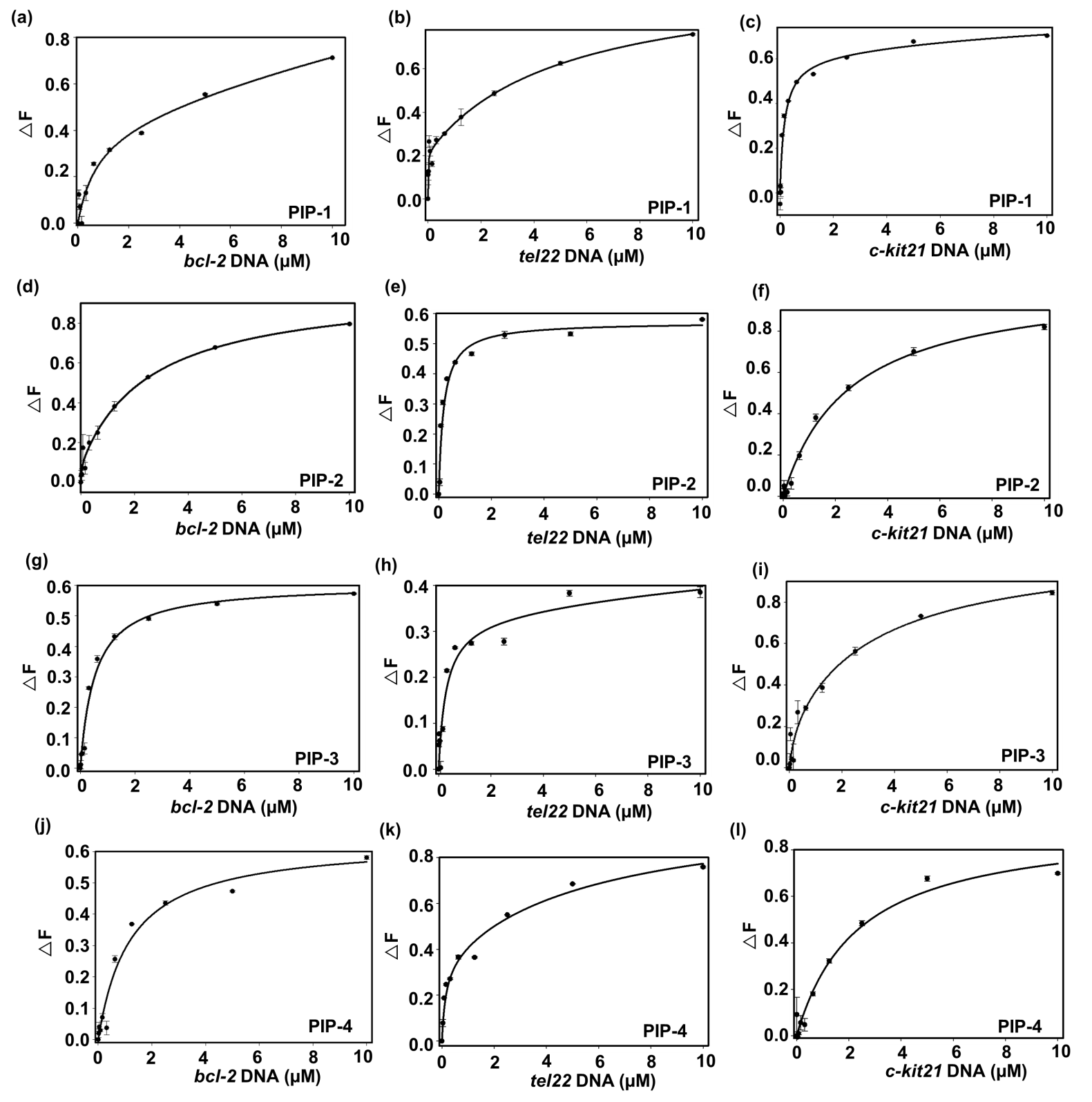


**Figure S4.** Fluorescence titration graph of Piperine analogs **(a)** PIP-1 **(b)** PIP-2 **(c)** PIP-3 and **(d)** PIP-4 with different G-quadruplex DNA (*tel22*, *bcl-2* and *c-kit21* DNA). The solid black line represents fitting the data on two-mode binding saturation mode.


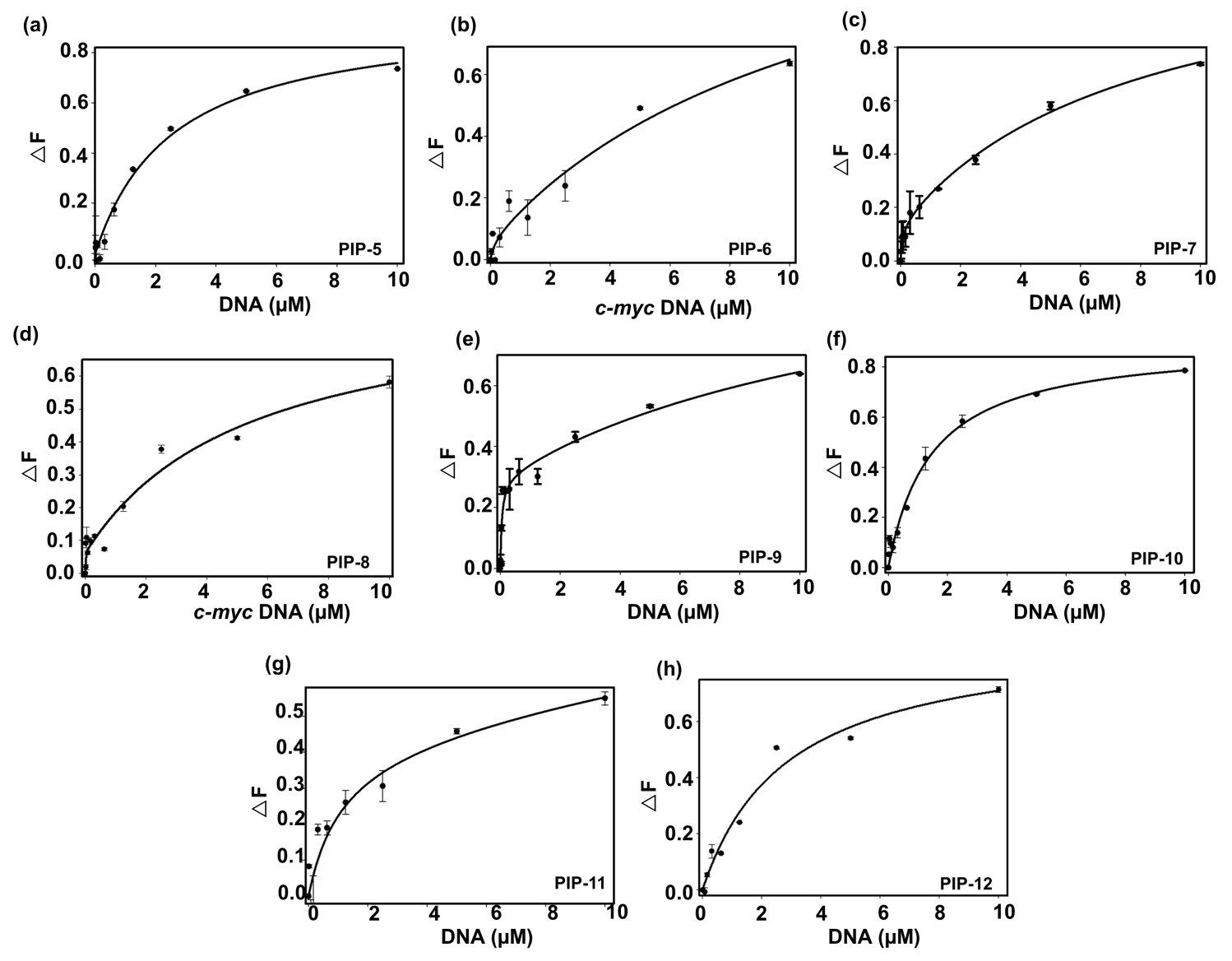


**Figure S5.** Fluorescence titration graph of other Piperine analogs with *c-myc* G4 DNA (PIP-5 to PIP-12). The solid black line represents the fitting of the data on two-mode binding saturation mode.


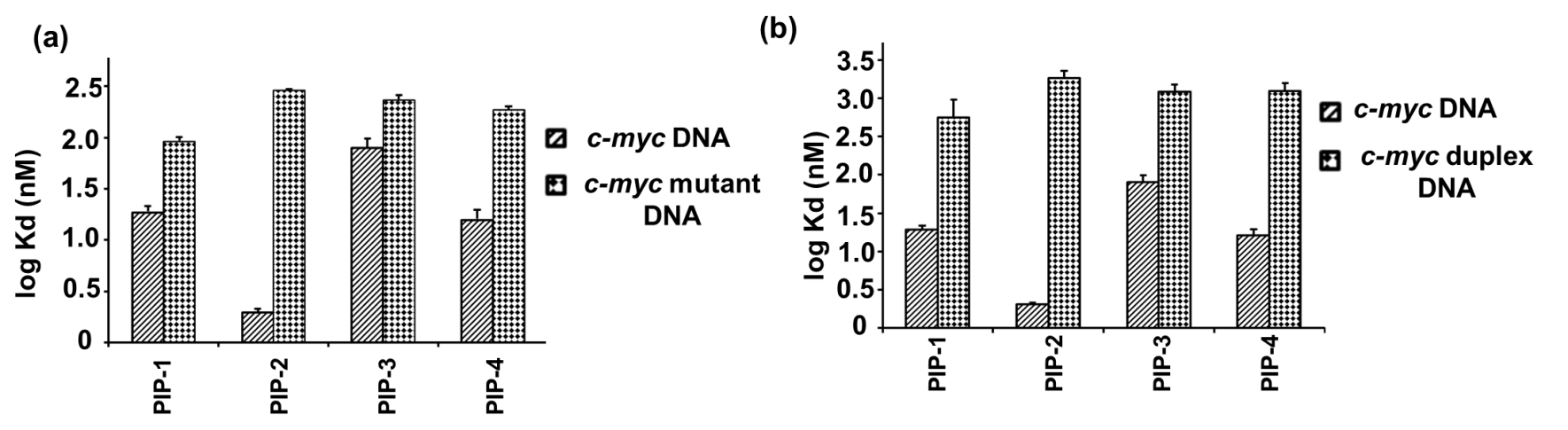
\

**Figure S6.** The comparative bar diagram of fluorescence binding assay of lead Piperine analogs- *c-myc* DNA with(a) *c-myc* mutant DNA, as well as (b) *c-myc* duplex DNA, represents mean±SE from three independent sets of experiments (for each experiment, duplicate wells were used)


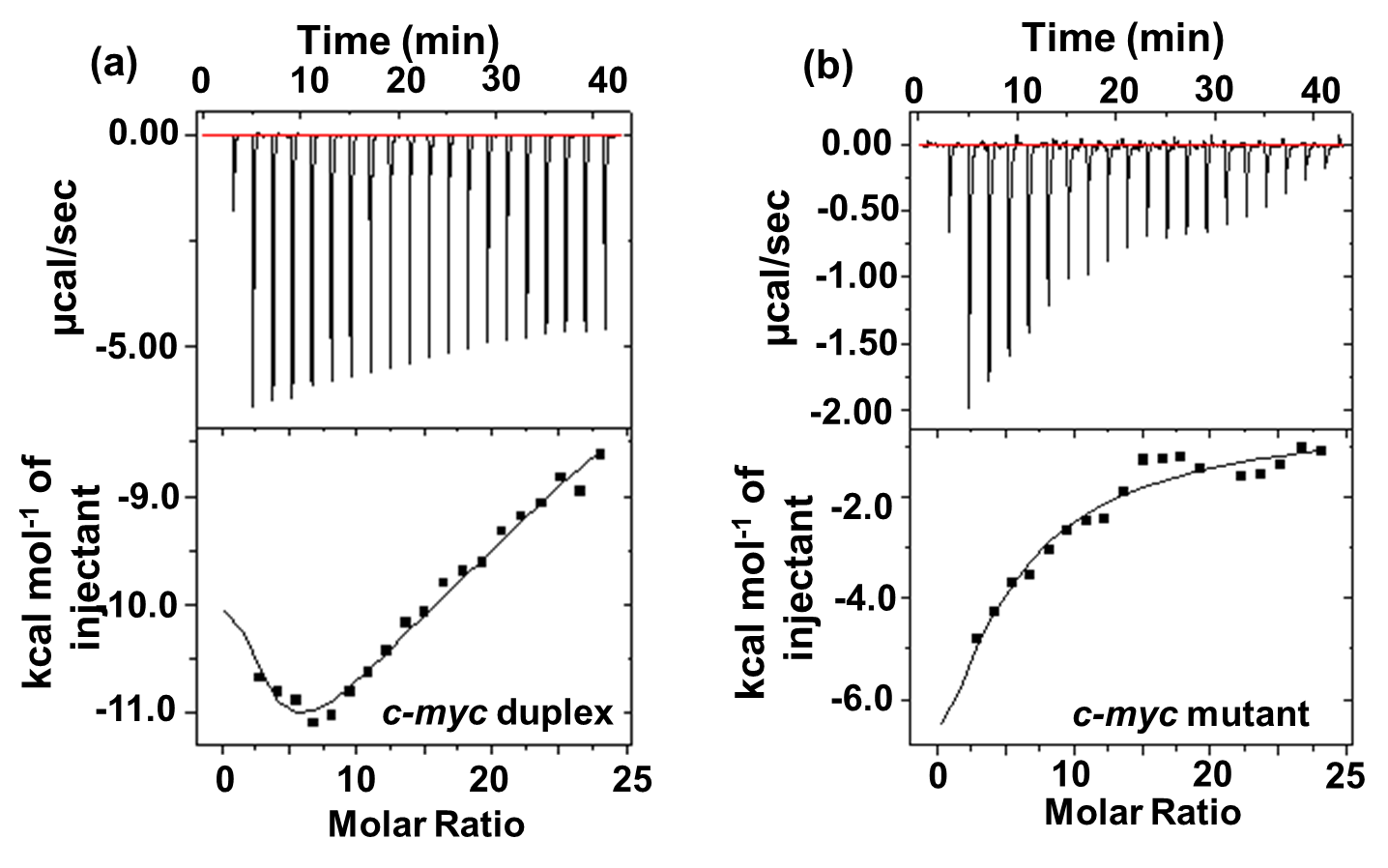


**Figure S7.** Isothermogram of lead Piperine analog PIP-2 with *c-myc* duplex and *c-myc* G4 mutant DNA obtained by Isothermal titration calorimetric analysis.


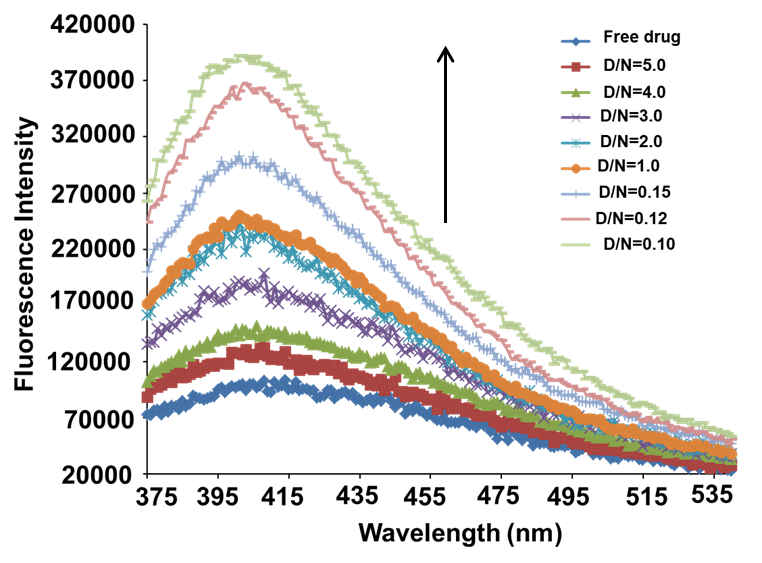


**Figure S8.** Fluorescence emission spectra of lead Piperine analog PIP-2 (5µM) with *c-myc* G4 DNA up to 0.10-5.0 in 10 mM phosphate buffer containing 50 mM KCl at 25 ֯C.


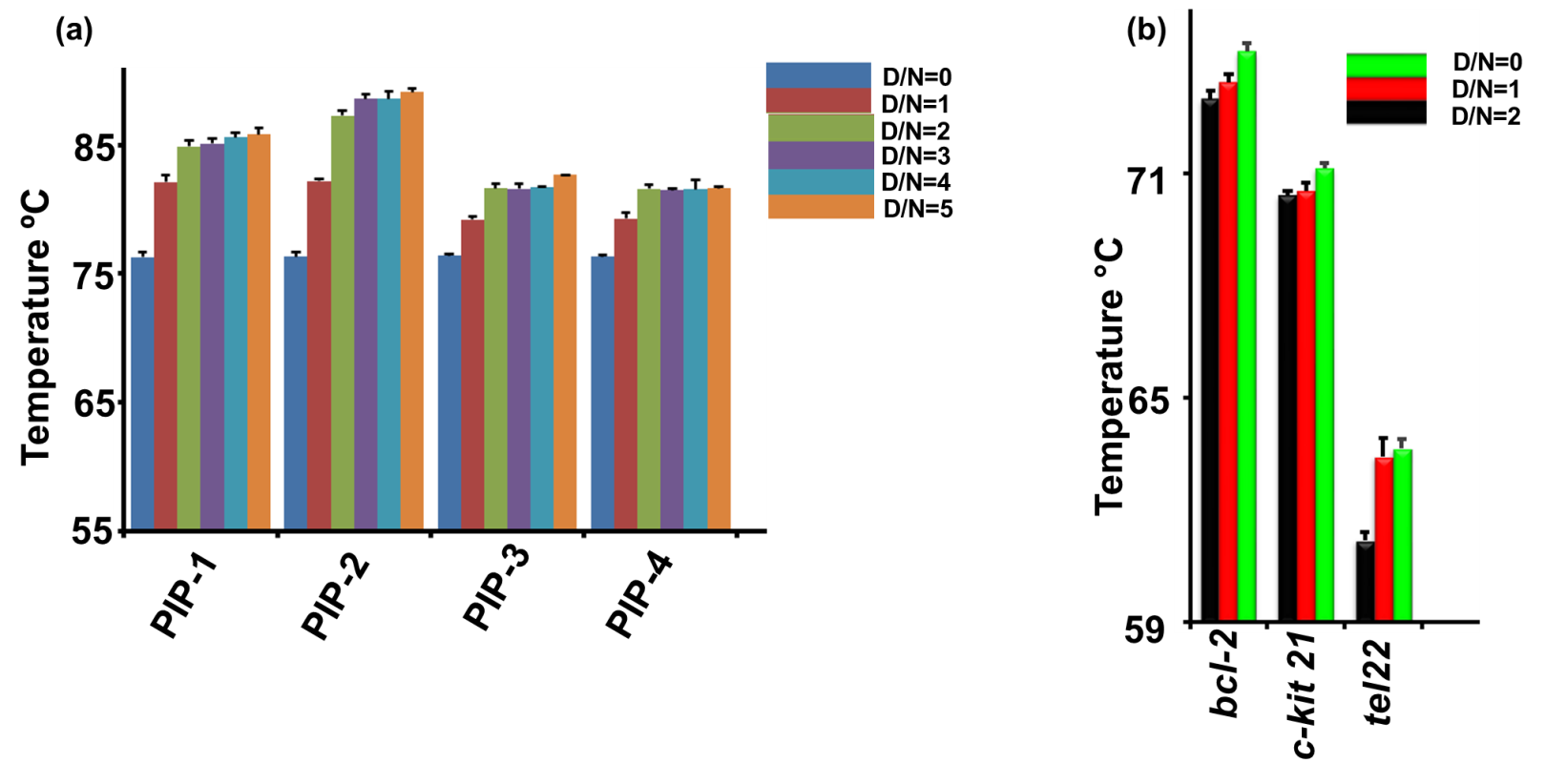


| **D/N ratio** | **PIP-1** | **PIP-2** | **PIP-3** | **PIP-4** |
| --- | --- | --- | --- | --- |
| **D/N=0** | **76.3±30** | **76.32±0.34** | **76.31±0.11** | **76.33±0.16** |
| **D/N=1** | **82.1±0.5** | **83.3±0.14** | **79.4±0.20** | **79.6±0.20** |
| **D/N=2** | **84.9±0.47** | **86.2±0.4** | **81±0.37** | **81.4±0.35** |
| **D/N=3** | **85.2±0.42** | **89.5±0.35** | **82.6±0.35** | **81.6±0.11** |
| **D/N=4** | **85.6±0.34** | **89.7±0.56** | **82.7±0.06** | **81.8±0.7** |
| **D/N=5** | **85.7±0.50** | **89.8±0.3** | **83.3±0.7** | **81.9±0.4** |

**Figure S9**. CD melting temperature values and graph lead Piperine analogs (PIP-1, PIP-2, PIP-3, and PIP-4) with *c-myc* G4 DNA and different G4 DNA.


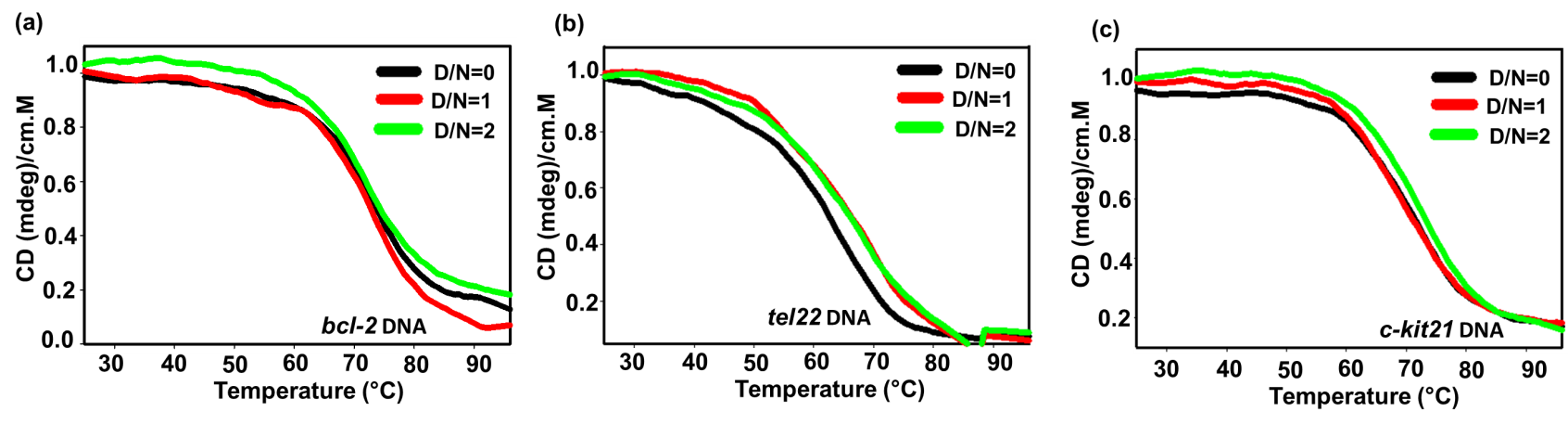


| **D/N ratio** | ***bcl-2*** | ***tel22*** | ***c-kit21*** |
| --- | --- | --- | --- |
| **D/N=0** | **73±0.20** | **61±0.48** | **70±0.44** |
| **D/N=1** | **73±0.28** | **63±0.49** | **70±0.4** |
| **D/N=2** | **74±0.23** | **63±0.22** | **71±0.51** |

**Figure S10.** CD melting spectra of lead molecule Piperine analog PIP-2 with different G-quadruplex DNAs (*bcl-2, tel22,* and *c-kit21)*.


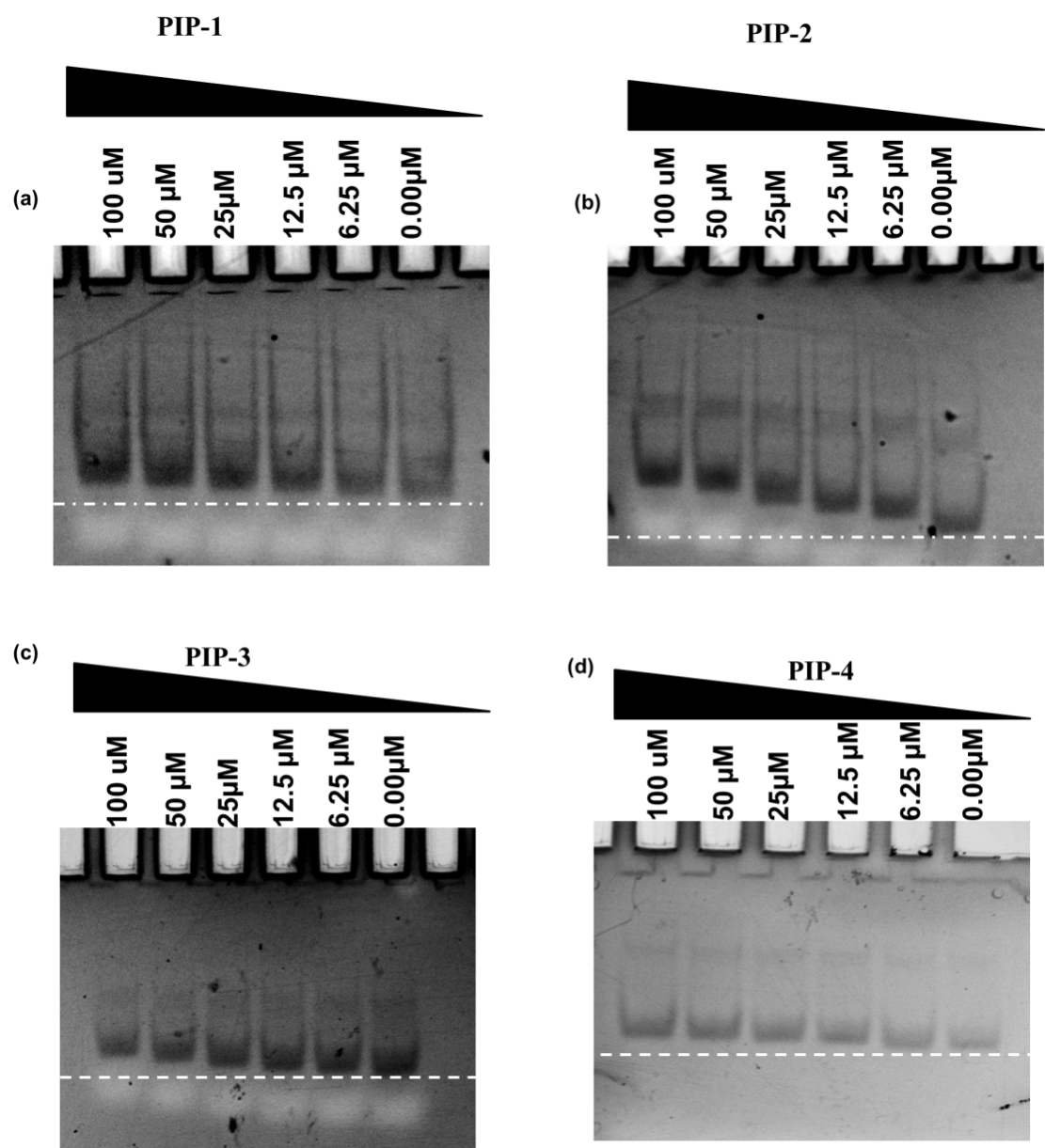


**Figure S11.** Gel retardation assay of Piperine analogs (0.00-100 µM) with *c-myc*G4 DNA. The white dash line represents the band shift upon migration of ligand DNA complex. (full-length gel images are provided in Fig. S17 a-d).


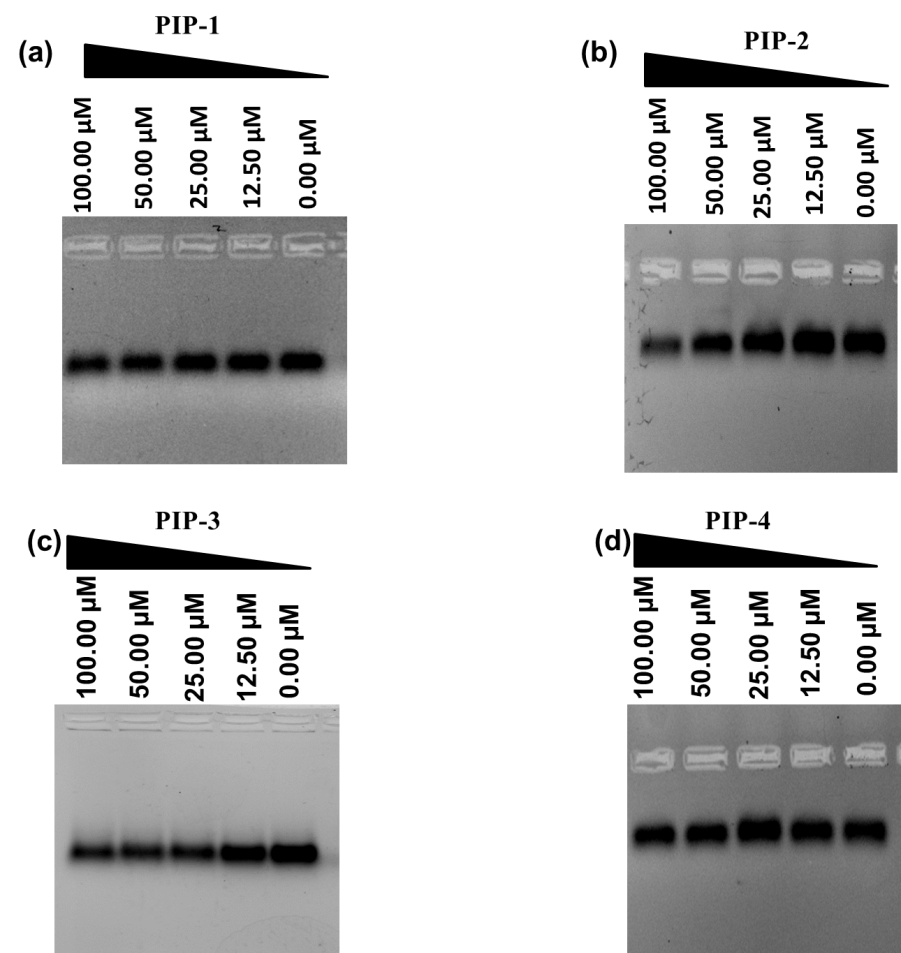


**Figure S12.** *Taq* Polymerase stops assay of Piperine analogs **(a)** PIP-1, **(b)** PIP-2 **(c)** PIP-3 **(d)** PIP-4. (0.00-100 µM) with *c-myc* G4 DNA (full-length gel images are provided in Fig. S18 a-d).


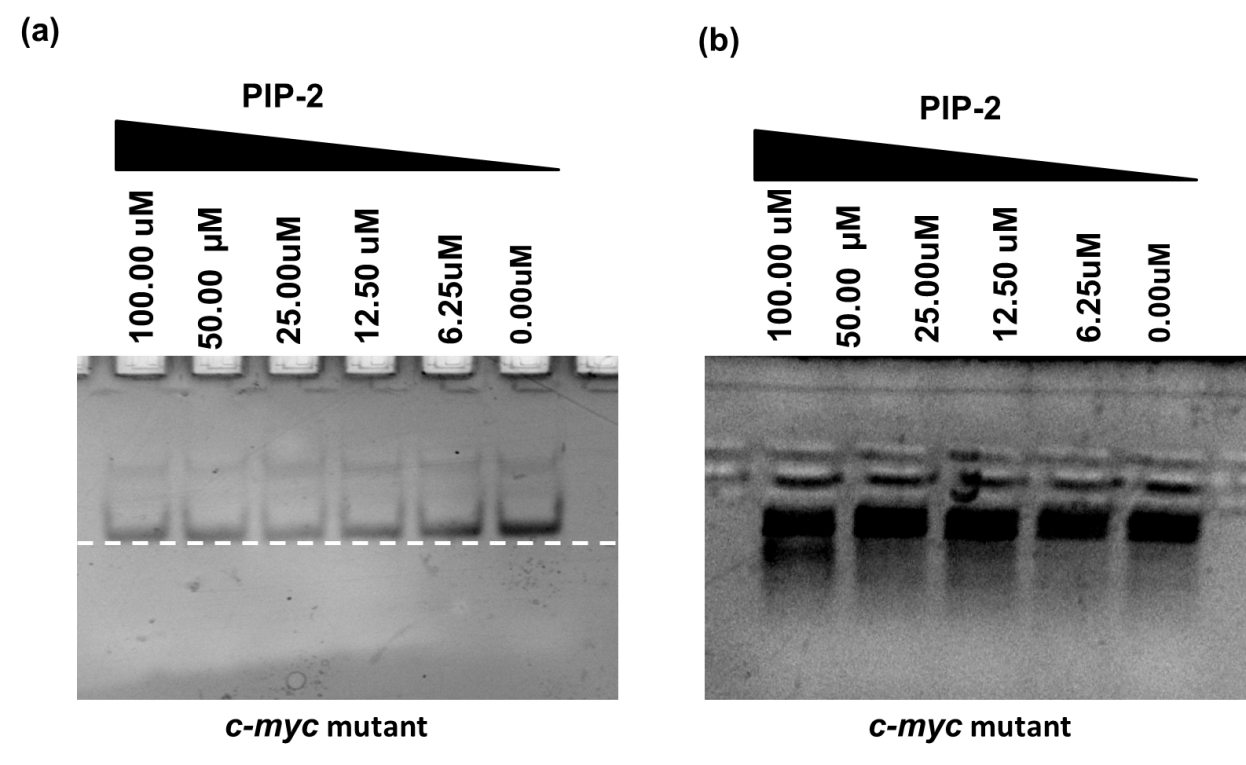


**Figure S13. (a)** Gel retardation and (b) PCR stop assay of lead molecule PIP-2 with *c-myc* G4 mutant DNA (full-length gel images are provided in Fig. S19 (a-b).


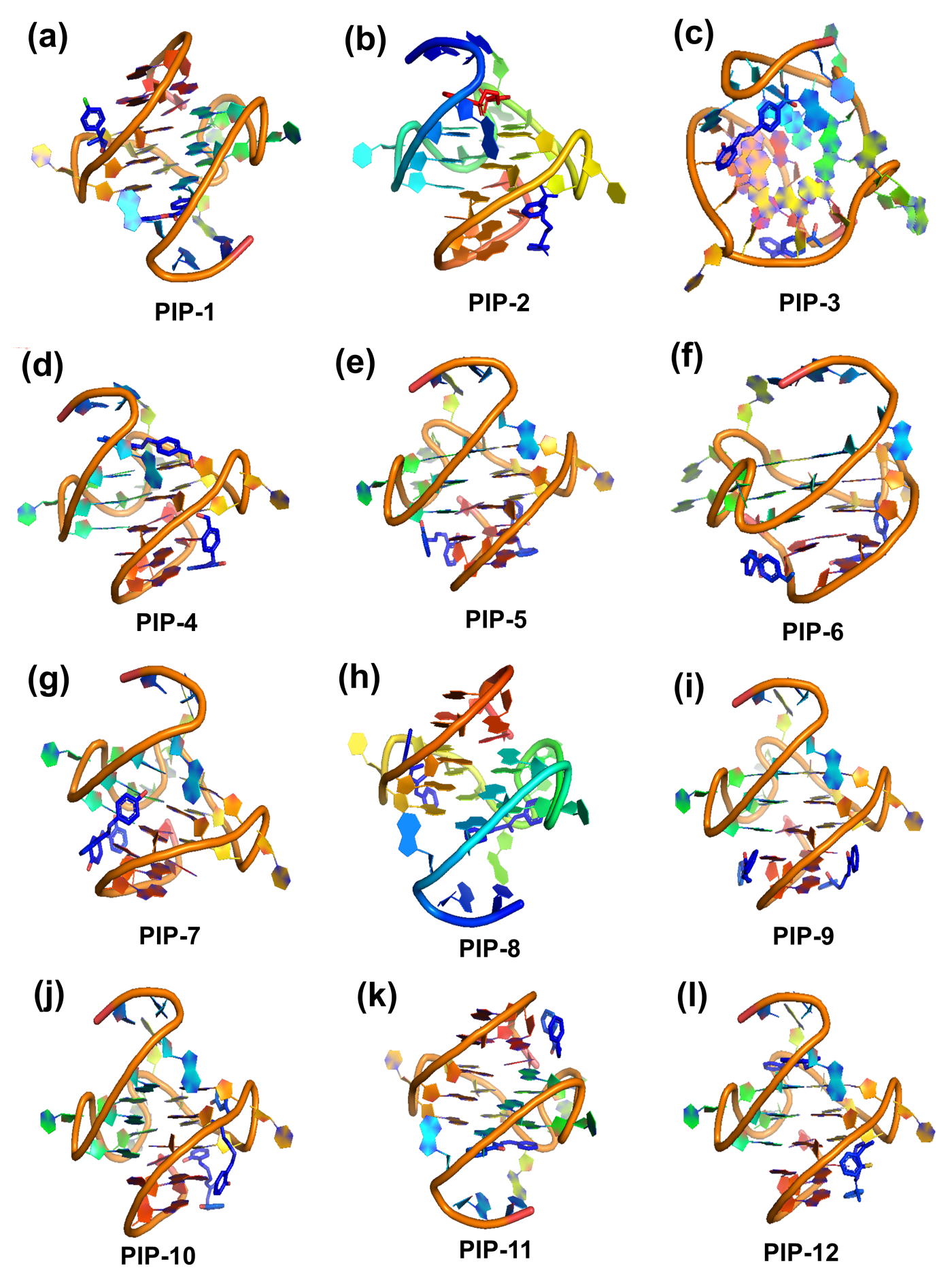


**Figure S14.** The images represent the interaction of different Piperine analogs (PIP-1 to PIP-12) with *c-myc* G4 DNA through molecular docking study


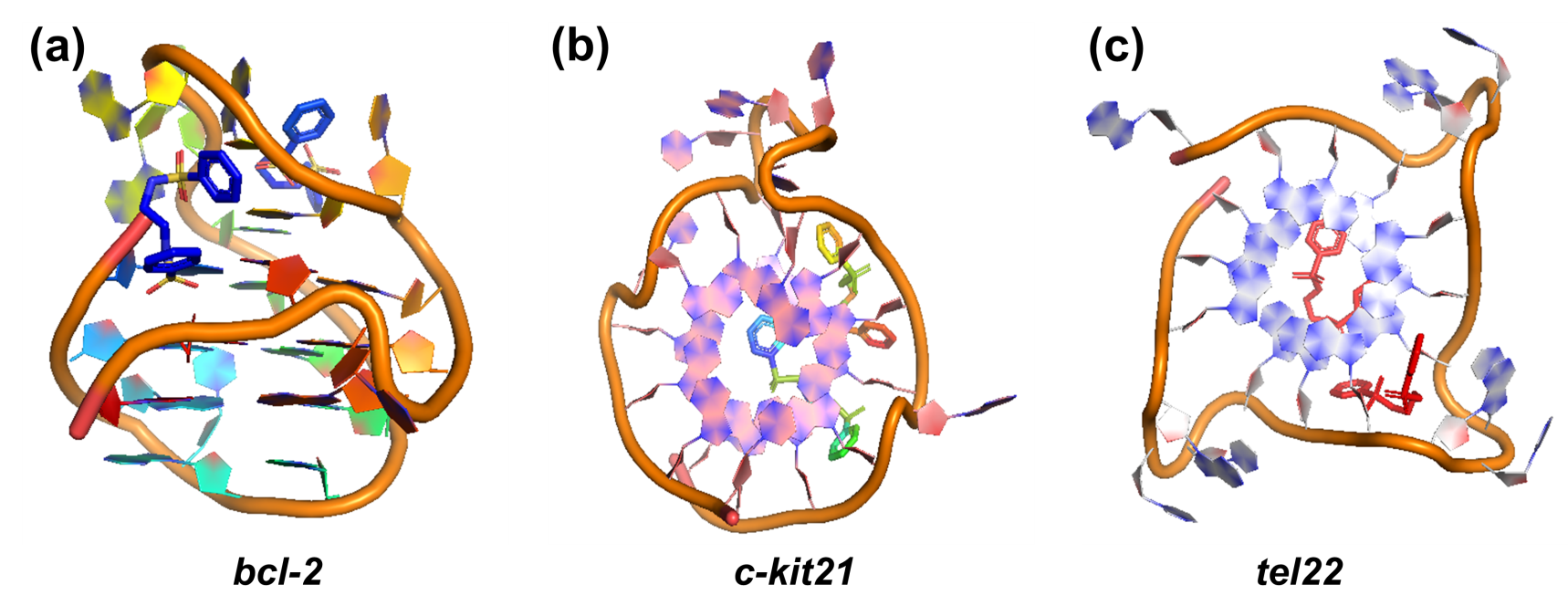


| **S.No.** | **DNA** | **Binding energy (kcal/mol)** |
| --- | --- | --- |
| 1. | *bcl-2* | -1.29 |
| 2. | *c-kit21* | -5.5 |
| 3. | *tel22* | -4.0 |

**Figure S15.** The images represent the interaction of Piperine analog PIP-2 with *bcl-2, c-kit21* and *tel22* G4 DNA through molecular docking study.


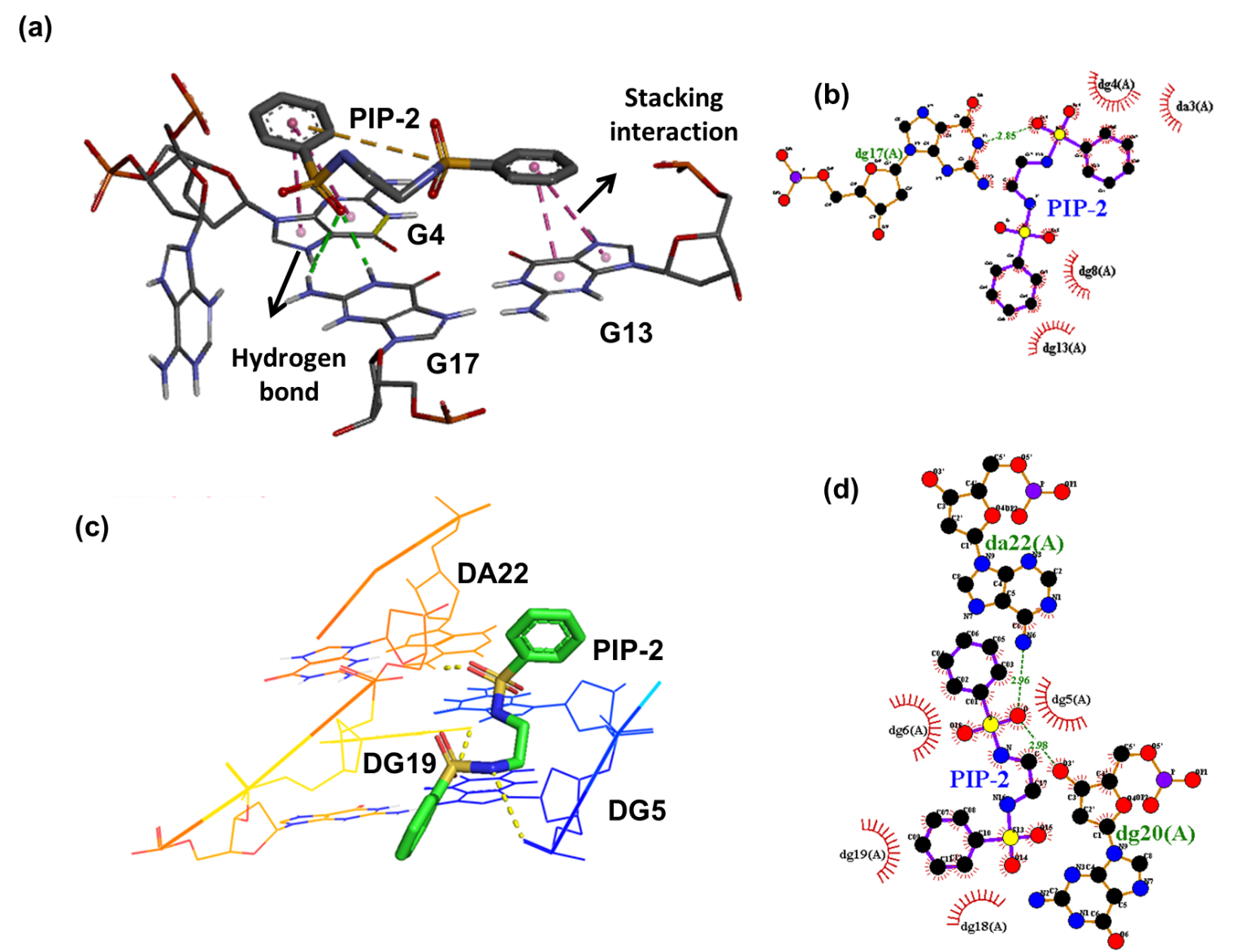


**Figure S16. (a)** Interaction of Piperine analog PIP-2 on the upper tetrad of *c-myc* G-quadruplex DNA. The green dotted line represents the formation of a hydrogen bond and the purple dotted line showing that PIP-2 form stacking interaction with the G13 and G4 residue of *c-myc* G4 DNA. **(b)** The image represents the interaction between PIP-2 and DNA via Ligplot+ and green dotted line showing the hydrogen bond between dG17 and PIP-2 ligand. The arc represents the nucleotide residues involved in the hydrophobic interaction with docked PIP-2 ligand. **(c)** The second PIP-2 molecule interacts with the *c-myc* G-quadruplex DNA through groove mode at the lower tetrad, and the yellow dotted line represents the hydrogen bond. **(d)** The image represents the interaction of the second molecule of PIP-2 with lower tetrad through Ligplot+ and green dotted line showing the hydrogen bond between dG20 and dA22 and PIP-2 ligand. The red arc represents the nucleotide residues involved in the hydrophobic interaction with docked PIP-2 ligand.


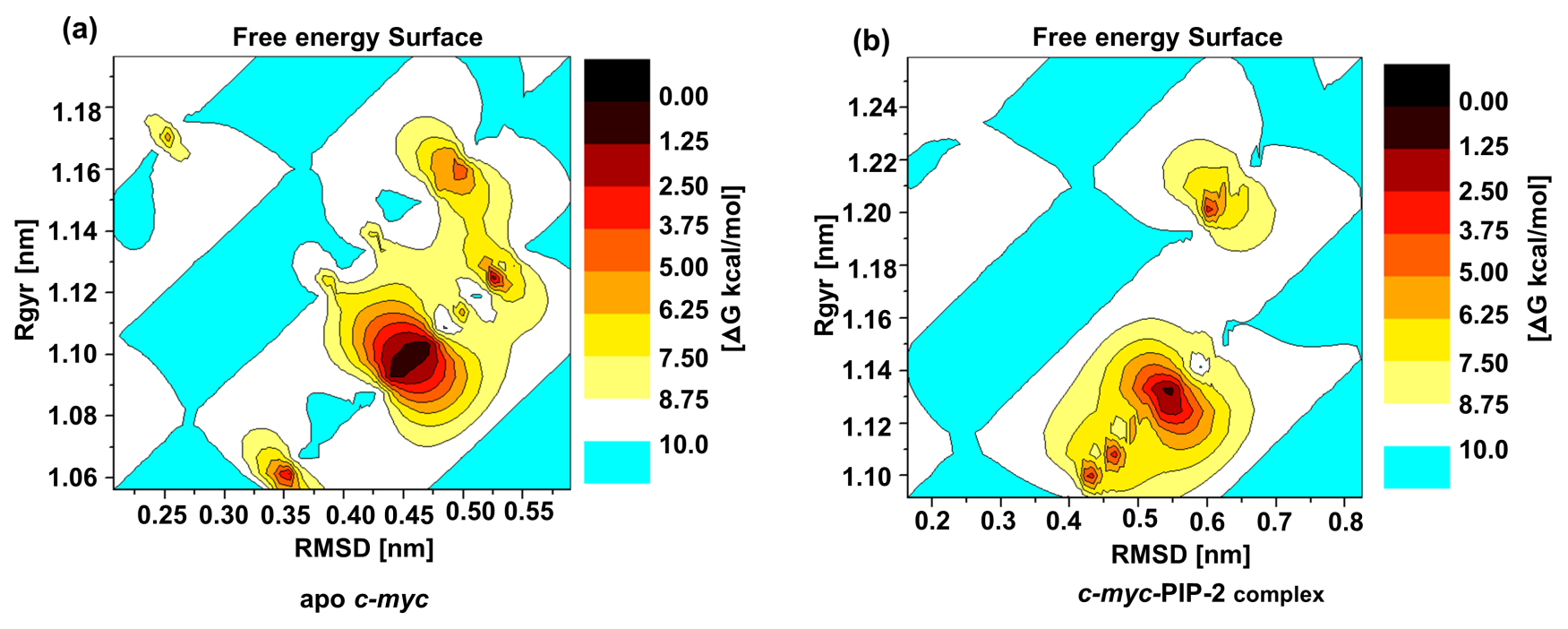


**Figure S17.**The contour graph represent the 2D free energy surface landscape on the basis of RMSD and Rg (Rgyr) of (a) apo *c-myc* and (b) *c-myc*-PIP-2 complex.


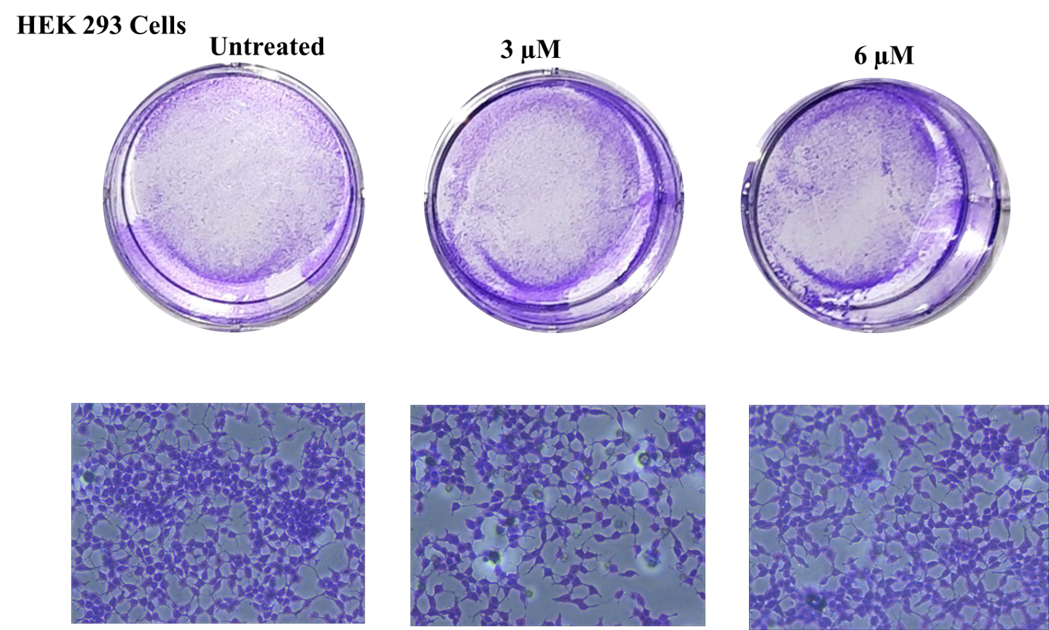


**Figure S18.Colony formation assay.** The inhibition effects of cell proliferation in HEK293 Cells after treatment with PIP-2 by using colony formation assay.


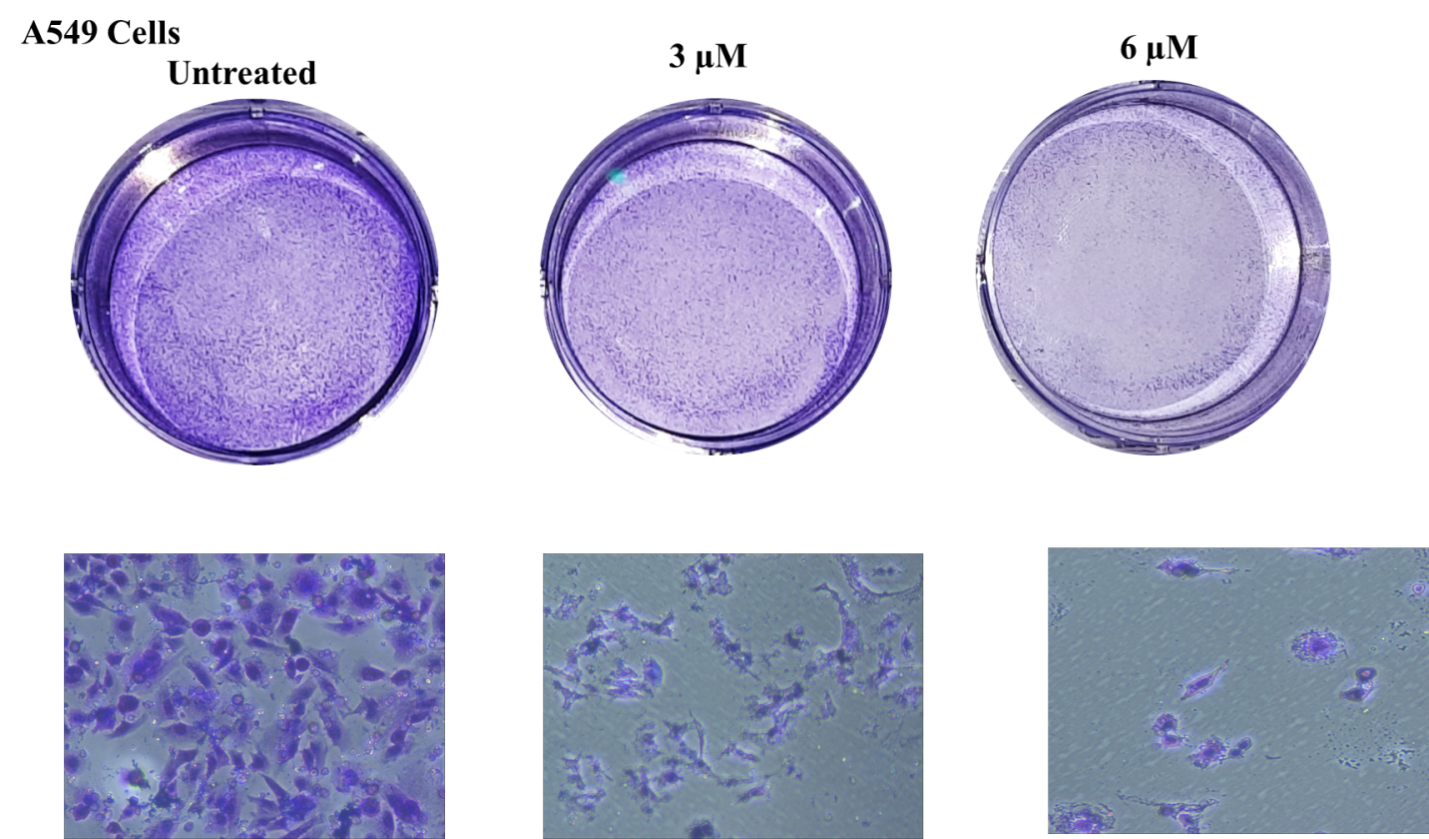


**Figure S19. Colony formation assay.** The inhibition effects of cell proliferation in A549 cells after treatment with PIP-2 by using colony formation assay.


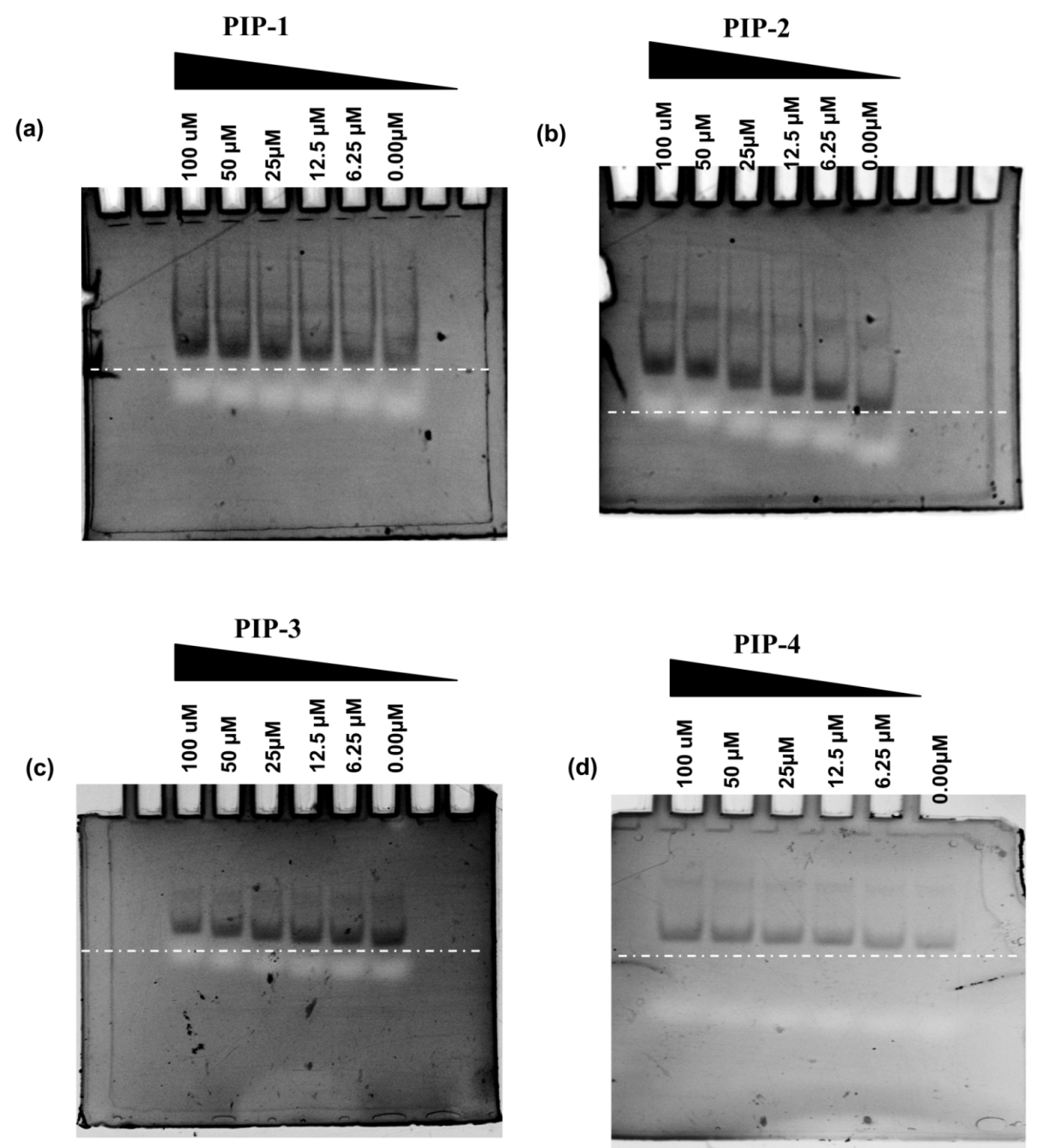


**Figure S20.** Full gel retardation images of Piperine analogs (PIP-1, PIP-2, PIP-3, and PIP-4) with *c-myc* G4 DNA. The white dash line represents the band shift due to the formation of the DNA-ligand complex.


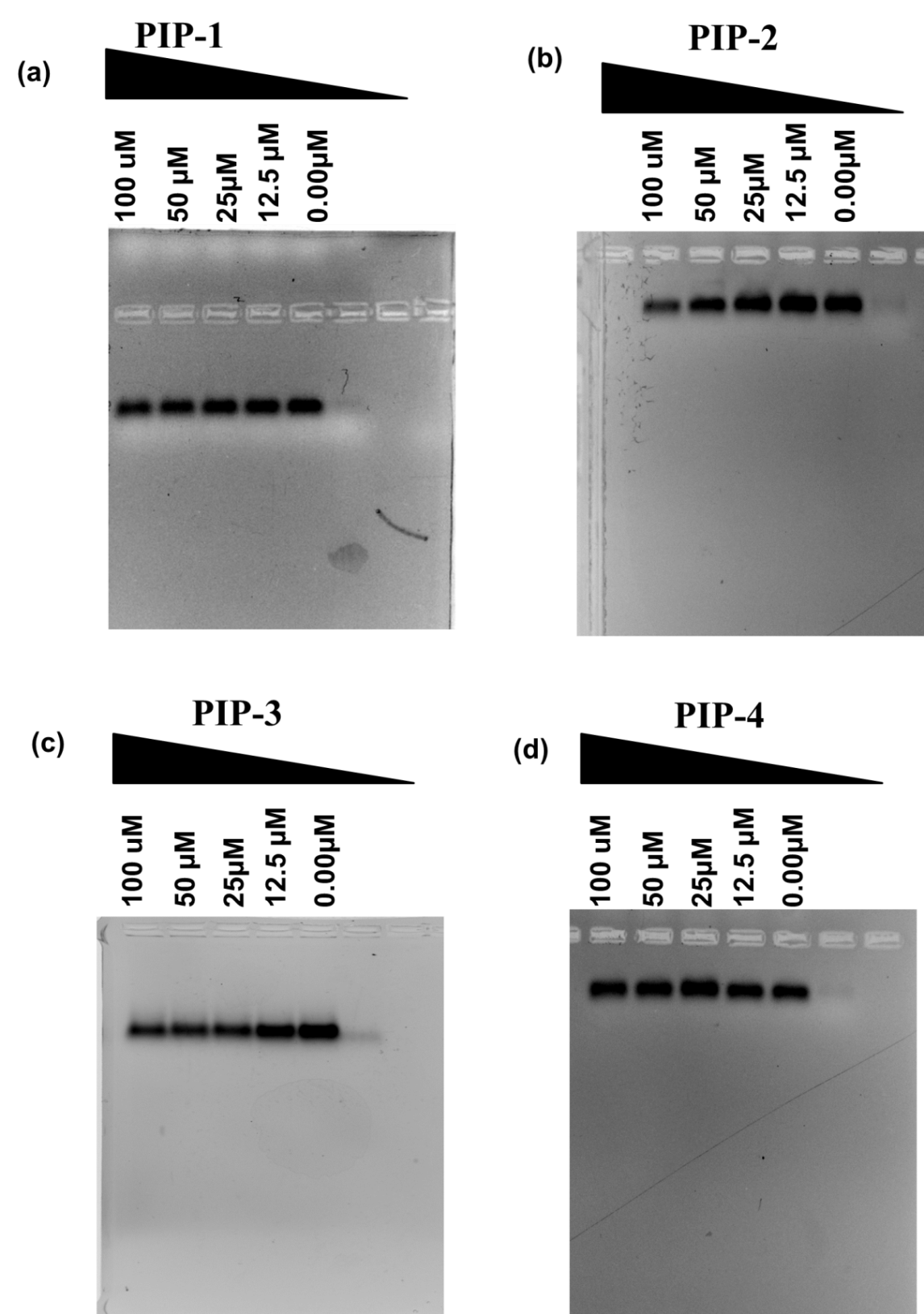


**Figure S21.** Full gel images of PCR stop assay Piperine analogs with *c-myc* G4 DNA.


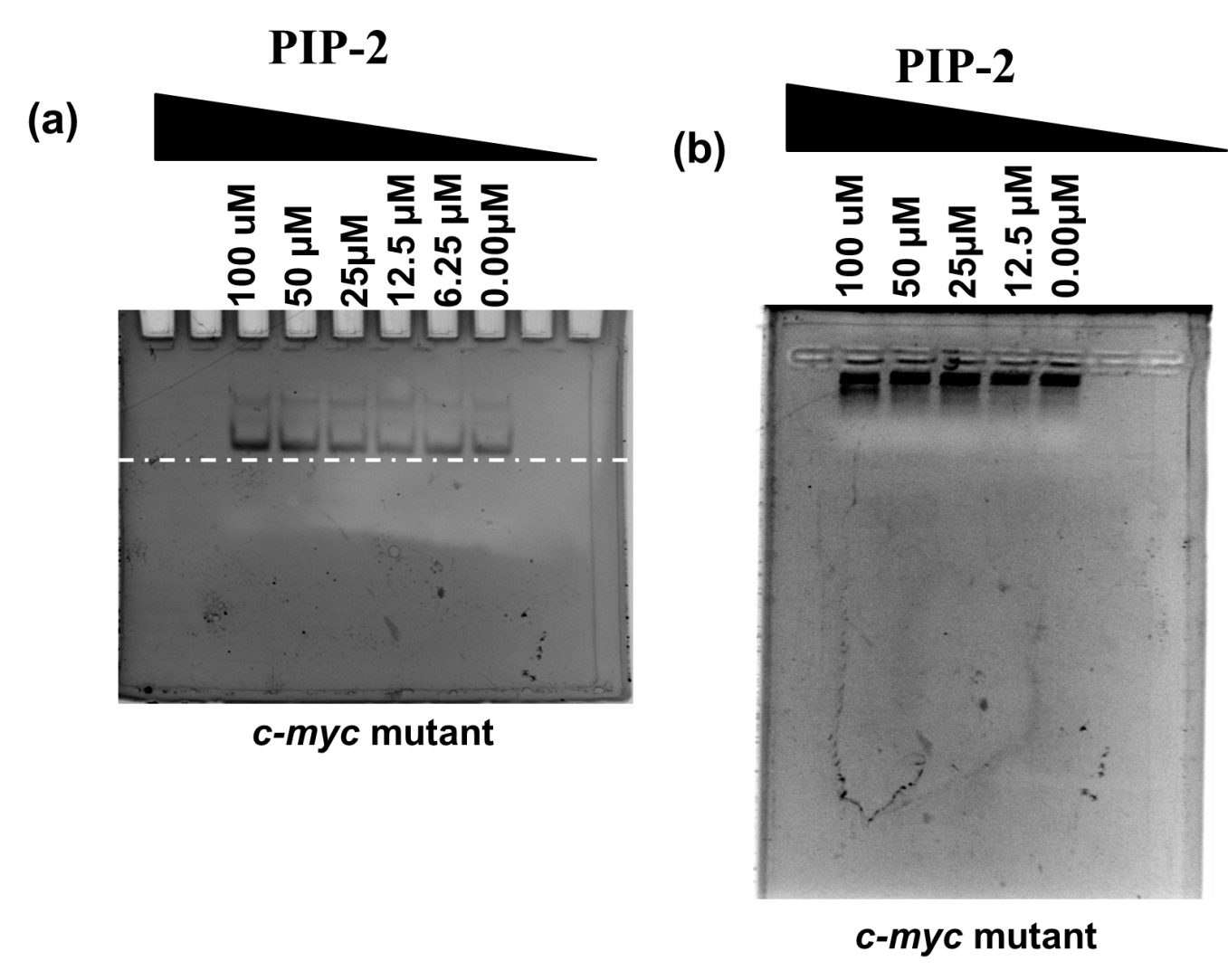


**Figure S22.** Full gel images of Gel retardation and PCR stop assay of Piperine analog PIP-2 with *c-myc* G4 mutant DNA. The white dash line represents the band shift due to the formation of DNA-ligand complex.


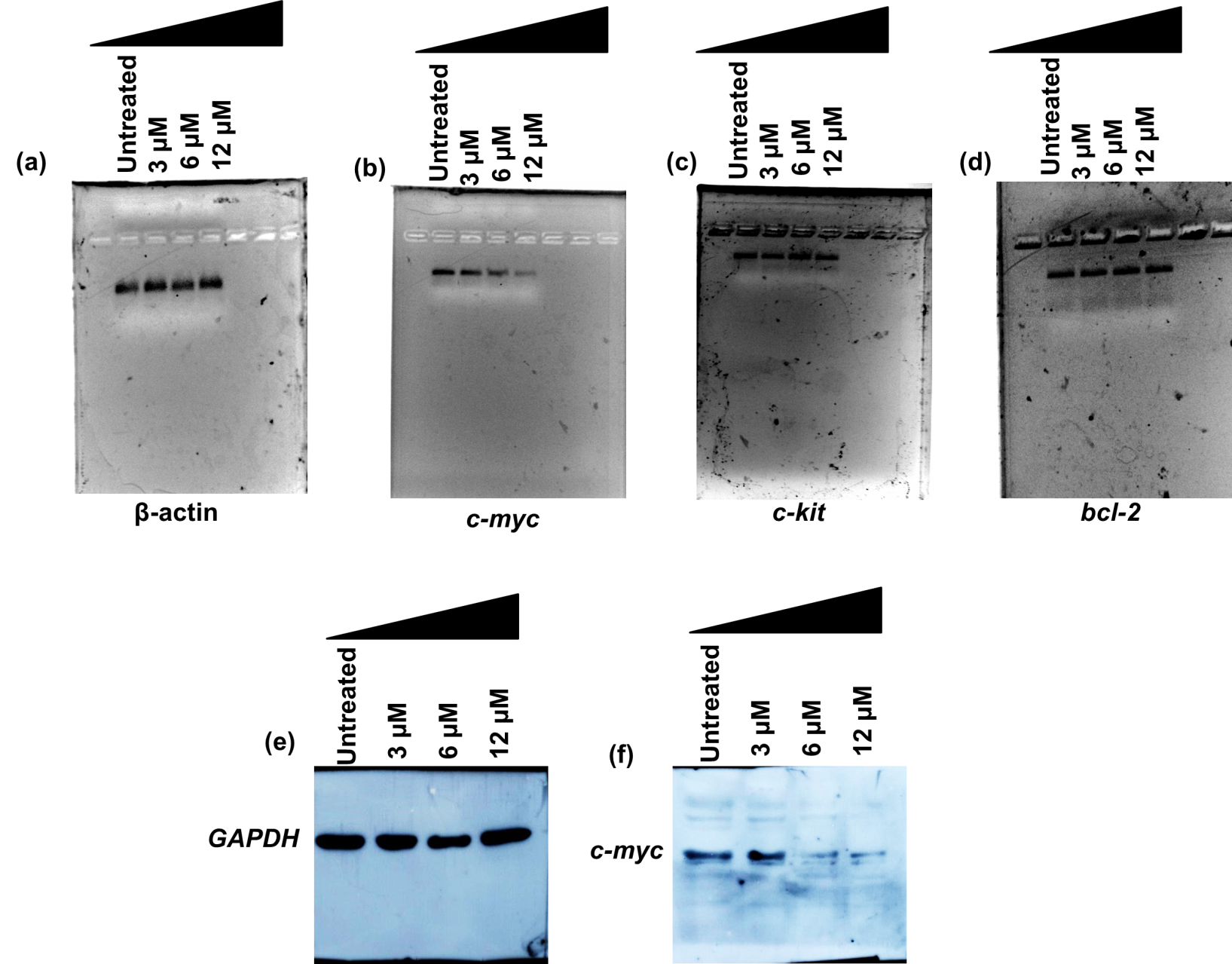


**Figure S23. (a-d)** Full gel images of RT-PCR gel images of PIP-2 treated A549 cells and showing the transcript level of *β-actin* and different oncogenes (*c-myc*, *c-kit*, *and bcl-2*). **(e-f)** Complete western blot image of PIP-2 with housekeeping gene *GAPDH* and *c-myc* oncogene.
